# Lysine Oxidation by LOXL2 in Single Strands Versus Triple Helices: Implications for Collagen‐Related Diseases

**DOI:** 10.1002/anie.1329257

**Published:** 2026-08-28

**Authors:** Laura M. Poller, Tomas Fiala, Helma Wennemers

**Affiliations:** ^1^ Laboratory of Organic Chemistry ETH Zürich Zürich Switzerland; ^2^ Department of Chemistry, Faculty of Science Masaryk University Brno Czech Republic

**Keywords:** collagen, extracellular matrix, lysine, lysyl oxidase, PPII helix

## Abstract

Collagen cross‐linking is essential for the structural integrity of the extracellular matrix. In several pathological conditions, the triple‐helical core of collagen is disrupted, and proline‐rich single strands occur. Lysyl oxidases (LOXs), enzymes that initiate cross‐linking by catalyzing the oxidative deamination of lysine to allysine residues, are upregulated in disease. Surprisingly little is known about the selectivity of LOXs. LOX‐initiated cross‐linking is believed to occur exclusively in the unstructured terminal domains of collagen, the telopeptides. But is this really the case? Herein, we show, with a combination of tailored chemical probes, enzymatic assays, CD and NMR spectroscopic analyses, that LOXL2 oxidizes Lys in proline‐rich single strands but not in triple helices. A comparison of Lys‐containing collagen model peptides (CMPs) with Lys‐containing telopeptides revealed comparable or higher oxidation rates in proline‐rich segments. The data suggest that LOXL2‐initiated cross‐linking occurs in pathologically disrupted triple‐helical domains, offering insights into aberrant extracellular matrix stiffening.

## Graphical abstract

The oxidation of Lys residues in proline‐rich single‐stranded collagen by LOXL2, a key enzyme for tissue maturation and linked to diseases, was revealed. Oxidation occurs at rates comparable to or higher than those of Lys in unstructured telopeptides, the well‐known native sites of Lysyl oxidase (LOX) activity. The findings suggest that LOX‐mediated cross‐linking of pathologically disrupted collagen contributes to matrix stiffening and connective tissue diseases.

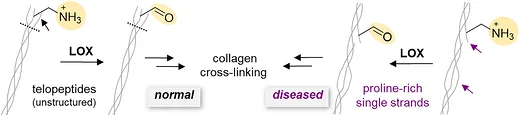

## 1 Introduction

Cross‐linking of collagen, the main component of the extracellular matrix (ECM) in mammals, is key to the strength and stability of any connective tissue [1, 2, 3, 4]. This process is initiated by lysyl oxidases (LOXs), enzymes that catalyze the oxidative deamination of lysine (Lys, K) to allysine residues (Figure 1a) [5, 6, 7, 8]. Subsequently, the allysine residues react spontaneously with other lysine, hydroxylysine, or allysine residues to establish intra‐ and intermolecular cross‐links between collagen strands. This LOX‐initiated cross‐linking is vital for normal tissue growth, tissue homeostasis, and wound healing [8, 9, 10]. Aberrant LOX expression is, however, associated with fibrosis and many types of cancer [10, 11, 12, 13, 14, 15]. Among the five isoforms, LOX and LOX‐like enzymes (LOXL) 1‐4, in particular LOXL2, have been associated with disease states [16, 17, 18]. Thus, a detailed understanding of LOXL2 and its substrate selectivity is important. In early studies, Kagan shed light on the reaction mechanism of bovine aorta LOX [5, 6, 7], evaluated sequence preferences [19, 20], and developed a peroxidase‐coupled assay to monitor LOX activity [21]. Surprisingly little, however, is known about the structural selectivity of LOX enzymes.

**FIGURE 1:**
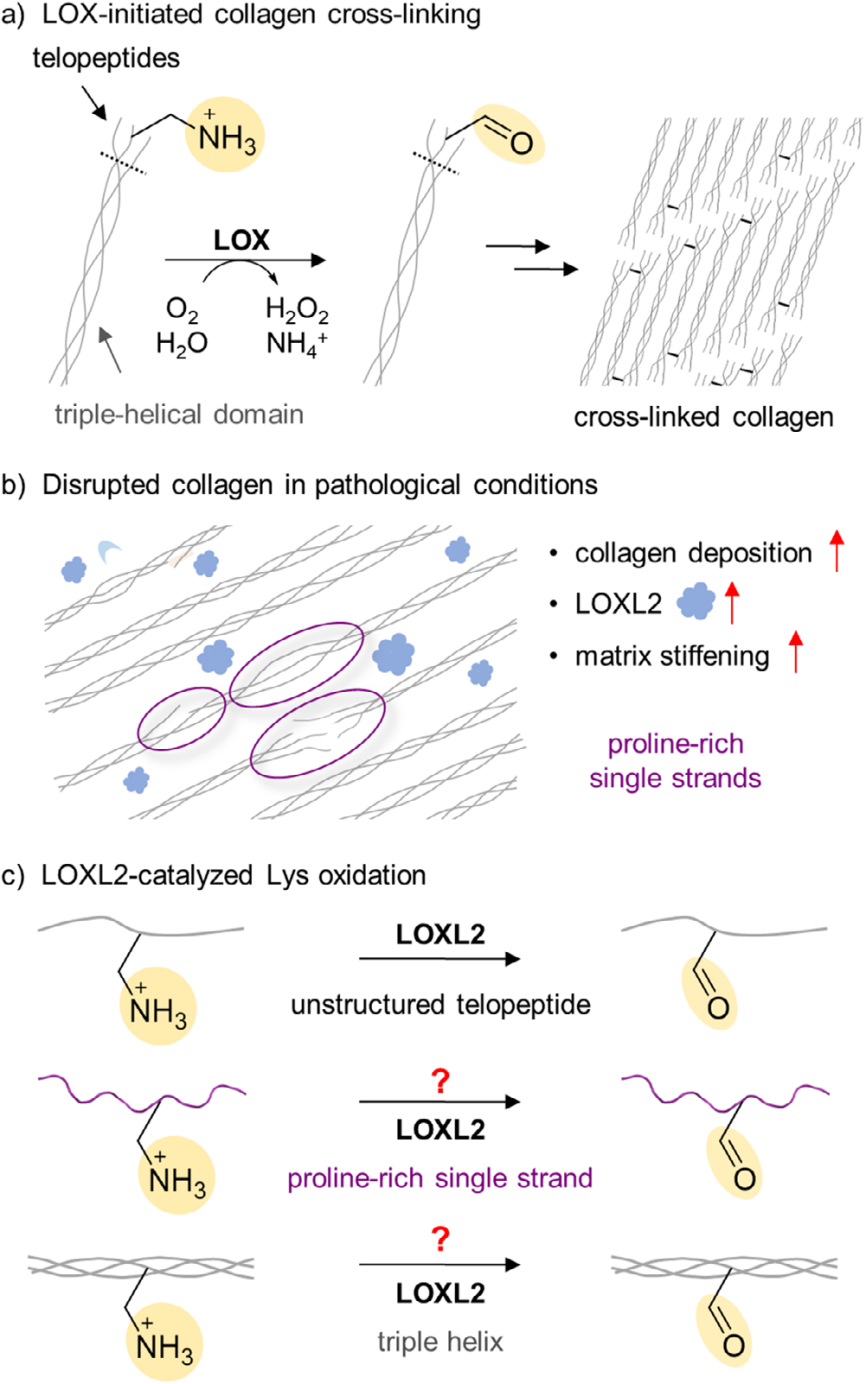
(a) LOXs catalyze the oxidative deamination of Lys to allysine residues in the unstructured collagen telopeptides, thereby initiating collagen cross‐linking. (b) Disrupted collagen triple helices in disease states. (c) Does Lys oxidation occur in proline‐rich single‐stranded collagen?

A hallmark of fibrous collagens is a triple‐helical domain with short, unstructured telopeptide domains at the termini (Figure 1a) [1, 2]. Each strand within the triple helix is composed of Xaa‐Yaa‐Gly repeating units, in which (2*S*)‐proline (Pro, P) and (2*S*,4*R*)‐4‐hydroxyproline (Hyp, O) are most frequent at the Xaa and Yaa positions, respectively [1, 22]. The Pro and Hyp residues preorganize each strand into left‐handed polyproline II (PPII)‐like helices that then intertwine into right‐handed triple helices [1].

LOX‐initiated allysine formation is generally believed to occur exclusively in the unstructured telopeptides (Figure 1a) [4, 22, 23]. In numerous connective tissue pathologies, including scars, cancer, osteogenesis imperfecta, and Alport syndrome, alterations disrupt the triple‐helical structure of collagen but maintain PPII‐helical single strands (Figure 1b) [13, 24, 25, 26, 27, 28]. In addition, under fibrotic conditions, LOX activity is upregulated [12, 13, 14]. The oxidation of Lys residues in disrupted triple helices might therefore be important in disease states but has been overlooked so far. These considerations led us to interrogate whether LOXL2 can oxidize Lys residues when embedded in a Pro‐rich, single‐stranded peptide, and possibly even within a triple helix (Figure 1c). This question is difficult to address in the complex ECM of natural tissue, which is why we used chemically defined structures.

Herein, we show that LOXL2 oxidation of Lys residues is not limited to telopeptide domains. A combination of enzymatic assays, CD, and NMR spectroscopic studies revealed that LOXL2 oxidizes Lys in Pro‐rich single‐stranded peptides, typical for disease states, with comparable or even higher activity than in unstructured telopeptide model compounds.

## 2 Results and Discussion

### 2.1 Representative Telopeptides and Collagen Model Peptides as Mimics of Single‐Stranded and Triple‐Helical Collagen

Collagen I is the most abundant natural collagen, and collagen III is highly expressed in the early stages of tissue formation and wound healing [10]. We therefore used sequences from these two collagen types, Ac‐QE**K**AH‐NH_2_ (telo I) and Ac‐GE**K**AG‐NH_2_ (telo III), as representative examples of unstructured telopeptides, in which Lys oxidation by LOXL2 occurs in nature [22, 29, 30, 31].

To interrogate whether LOXL2 can oxidize Lys residues in PPII‐helical single‐stranded and possibly even triple‐helical peptides, we designed synthetic collagen model peptides (CMPs). CMPs are valuable mimics of collagen and have provided key insights, for example, into the factors contributing to the stability of collagen triple helices [1, 32, 33, 34, 35, 36, 37]. These studies also established design principles for synthetic collagens with different levels of stability, and thus, different equilibrium constants between non‐assembled single‐stranded and triple‐helical collagen peptides [1]. Building on these design principles [36, 37], we used N‐ and C‐terminally capped (Gly‐Pro‐Hyp)_x_ repeats of different lengths to vary the triple helix/single strand equilibrium. In the helical domain of fibrillar collagen, Lys residues occur at both the Xaa and Yaa positions [1, 30, 31]. A Lys residue was therefore embedded either at the Xaa or the Yaa position in the middle of the CMPs (Table 1). We prepared a series of 18‐mers (**18‐X** and **18‐Y**) and 24‐mers (**24‐X** and **24‐Y**), anticipating that triple helix stability and therefore the triple helix/single strand ratio would increase from the 18‐mers to the 24‐mers (Table 1).

**TABLE 1:** CMPs of different lengths with Lys residues either at the Xaa (green) or Yaa position (blue) and *T*_m_ values of the respective triple helices.

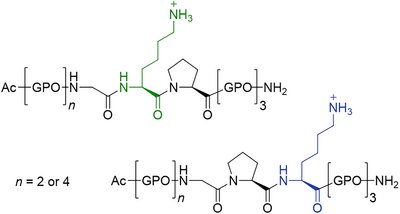		
Code	Sequence	*T*_m_ (°C)
**18‐X**	Ac‐[GPO]_2_‐G**K**P‐[GPO]_3‐_NH_2_	21.7
**18‐Y**	Ac‐[GPO]_2_‐GP**K**‐[GPO]_3_‐NH_2_	24.7
**24‐X**	Ac‐[GPO]_4_‐G**K**P‐[GPO]_3_‐NH_2_	50.0
**24‐Y**	Ac‐[GPO]_4_‐GP**K**‐[GPO]_3_‐NH_2_	51.9

*T*_m_ values were determined by thermal denaturation with CD spectroscopic monitoring at 225 nm using CMP solutions (200 µM) in phosphate‐buffered saline (PBS), pH 7.4, at a heating rate of 1°C/114 s. *n* = 2 independent experiments; standard deviation (SD) ≤ 0.9°C.

Circular dichroism (CD) spectra of all four CMPs corroborated their expected PPII conformation, with a characteristic maximum at 225 nm and a minimum close to 200 nm (Figure S1). Thermal denaturation experiments monitored by CD spectroscopy at 225 nm provided sigmoidal melting curves with sharp transitions, typical for the unfolding of collagen triple helices (Figure S2). Their inflection points, the melting temperatures (*T*_m_), reflect the relative thermal stability of the collagen triple helices (Figure S2). The triple helices formed by the 18‐mers have *T*_m_ values of 21.7°C (**18‐X**) and 24.7°C (**18‐Y**) (Tables 1 and S1). As expected, the 24‐mer CMPs form significantly more stable triple helices with *T*_m_ values of 50.0°C (**24‐X**) and 51.9°C (**24‐Y**) [38]. Across all CMPs, triple helices with Lys at the Yaa position are slightly more stable (∆*T*_m_ = 2°C – 3°C) compared to those with Lys at Xaa.

### 2.2 LOXL2 Oxidizes Lys Residues in Pro‐Rich Single Strands With Similar or Higher Activity as in Unstructured Telopeptides

The enzymatic activity of LOXs can conveniently be measured by the so‐called Amplex Red assay [21, 39, 40]. This assay monitors LOX activity by measuring the rate of H_2_O_2_ formation, which is generated alongside the allysine residue through a horseradish peroxidase (HRP)‐coupled assay (Figure 2a). Sodium borate buffer at pH 8.5 containing 0.1% bovine serum albumin (BSA) provides optimal conditions for the Amplex Red assay [40, 41]. Reassuringly, in this buffer, thermal denaturation studies with the triple‐helical CMPs **24‐X** and **24‐Y** revealed *T*_m_ values comparable to those in PBS buffer (Tables 1, S1, and S2 and Figure S3). Based on the *T*_m_ values, the Amplex Red assays were performed at 29°C. At this temperature, the 18‐mer CMPs should be mainly single‐stranded and the 24‐mer CMPs triple‐helical.

**FIGURE 2:**
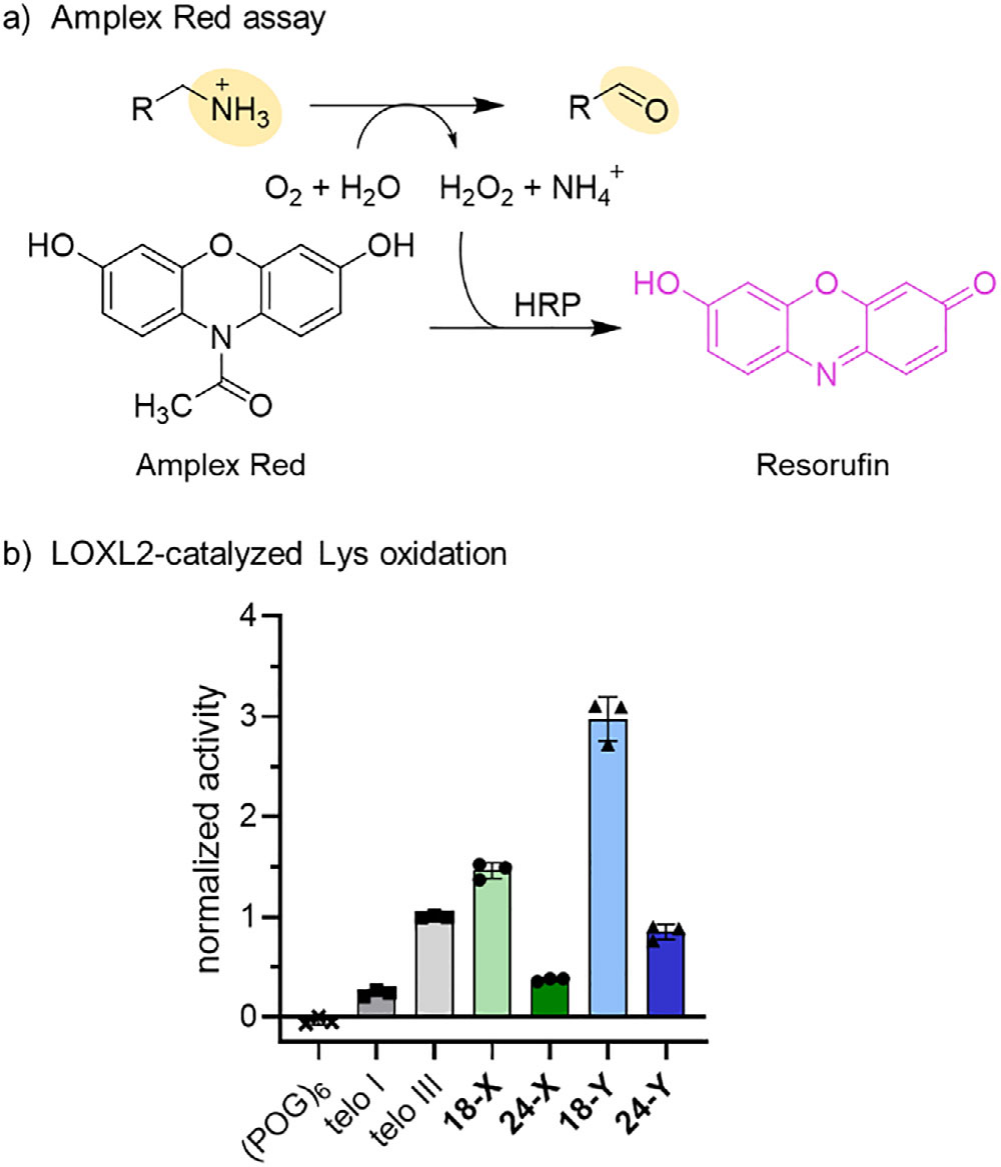
(a) The Amplex Red assay for monitoring LOXL2 activity. (b) LOXL2‐catalyzed oxidation of Lys at the Xaa (green) or Yaa position (blue) in CMPs of varying lengths, as well as telopeptides Ac‐QE**K**AH‐NH_2_ (telo I) and Ac‐GE**K**AG‐NH_2_ (telo III). Representative oxidation rates were normalized to telo III and are presented as mean ± SD; *n* = 2–3 independent experiments, each performed in technical triplicate. Concentrations: *c*(LOXL2) = 50 nM = 4.3 µg/mL, *c*(peptide) = 4 mM.

Prior to assessing the oxidation rate of LOXL2, each CMP was annealed overnight to establish the equilibrium between single strands and triple helices. To mimic pathological states, in which LOXL2 and collagen deposition are elevated [8, 16, 17, 18], we used comparatively high concentrations of LOXL2 (50 nM = 4.3 µg/mL) and peptides (4 mM). Ac‐(POG)_6_‐NH_2_ ((POG)_6_), a single‐stranded CMP that lacks a Lys residue, was used as a negative control. Telo III was used as a reference for normalizing the observed rates.

The Amplex Red assay revealed that LOXL2 oxidizes Lys in each of the examined CMPs (Figure 2b, green and blue, S4, and Table S3). As expected, LOXL2 activity was not detected with the negative control (POG)_6_ that lacks a Lys residue but with the reference telopeptides (Figure 2b, gray). Notably, the oxidation rate of telo III was comparable to that of the 24‐mer CMPs **24‐X** and **24‐Y** (Figure 2b, dark green and blue), while that of telo I was lower. CMPs **18‐X** and **18‐Y** were even oxidized faster than telo III (Figure 2b, light green and blue). These findings show that LOXL2 activity is not limited to Lys residues in unstructured peptides but is compatible with Pro‐rich collagen peptides that have a high tendency to form a PPII‐helical conformation.

Among the CMPs, LOXL2 oxidized the 18‐mer with Lys at the Yaa position (**18‐Y**) fastest, followed by the 18‐mer with Lys at the Xaa position (**18‐X**; Figure 2b, light blue and light green). The same relative trend was observed for the 24‐mers. **24‐Y** was oxidized at a slightly higher rate than **24‐X** (Figure 2b, dark blue and dark green). This result implies that a Lys residue at a Yaa position is more accessible to LOXL2 compared to a Lys at Xaa. Notably, the higher oxidation rate coincides with a higher frequency of Lys residues at Yaa compared to Xaa positions in natural collagen types I and III [30, 31]. Comparison of the 18‐ and 24‐mers shows that LOXL2 oxidizes Lys in the shorter, mainly single‐stranded, CMPs significantly faster than in the longer, mainly triple‐helical, CMPs.

High‐resolution mass spectrometry (HRMS) detected the formation of the aldehyde product, corroborating the validity of the Amplex Red assay for detecting LOXL2 activity (Figure S5).

### 2.3 LOXL2 Oxidizes Lys in Single‐Stranded but Not Triple‐Helical Collagen

Next, we examined whether LOXL2 oxidizes Lys even in triple‐helical collagen. Collagen triple‐helix assembly is an equilibrium process (Figure 3a) [1]. Regardless of their stability, triple helices are in equilibrium with the respective single strands. We, therefore, interrogated whether the observed activity of LOXL2 toward the 24‐mers **24‐X** and **24‐Y** (Figure 2b) arises from oxidation of triple helices or rather single strands. To quantify the proportions of triple helices and single strands under the assay conditions, we prepared analogs of CMPs **18‐Y** and **24‐Y** with a ^13^C‐labeled Gly residue (Ac‐(GPO)_2_GP**K**GPO**^13^G**POGPO‐NH_2_ and Ac‐(GPO)_4_GP**K**GPO**^13^G**POGPO‐NH_2_), and analyzed the triple helix/single strand ratio by quantitative ^13^C nuclear magnetic resonance (NMR) spectroscopy. Prior to NMR analysis, these labeled CMPs were annealed using the same procedure as in the enzymatic assays.

**FIGURE 3:**
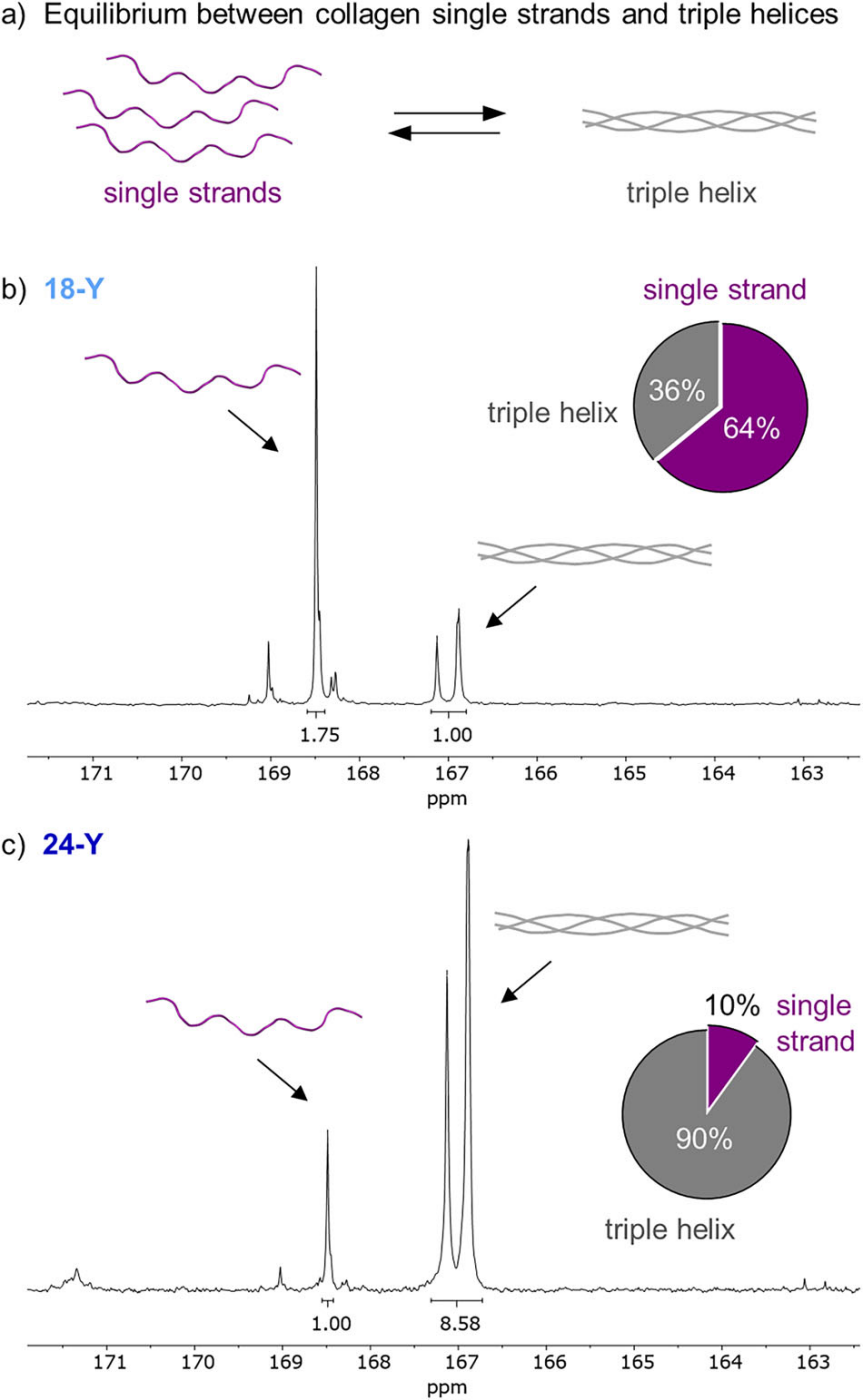
(a) Equilibrium between collagen single strands and triple helices. ^13^C NMR spectra of ^13^C‐labeled **18‐Y** (b) and **24‐Y** (c) show the ratio of single‐stranded (*δ* = 168.5 ppm) and triple‐helical (*δ* = 167.0 ppm) CMPs. The spectra of the CMPs (4 mM) were recorded in assay buffer (50 mM sodium borate containing 0.1% BSA, pH 8.5) at 29°C after annealing overnight.

The ^13^C NMR spectrum of a 4 mM solution of ^13^C‐labeled **18‐Y** revealed a ratio between triple helices and single strands of 36:64 (Figure 3b). A solution of ^13^C‐labeled **24‐Y** at the same concentration contained only ∼10% single strands, confirming that this CMP forms predominantly triple‐helical assemblies (Figure 3c). These different propensities for triple helix formation of CMPs **18‐Y** and **24‐Y** are consistent with their significantly different thermal stabilities (*T*_m_ = 24.7°C (**18‐Y**) vs. 51.9°C (**24‐Y**); Table 1).

The coexistence of single strands and triple helices in the solutions of **18‐Y** and **24‐Y** implies that the observed LOXL2 activity (Figure 2b) cannot be unambiguously attributed to either species. To distinguish between Lys oxidation in Pro‐rich single‐stranded versus triple‐helical CMPs, we compared LOXL2 activity in samples of **18‐Y** and **24‐Y** with equal concentrations of single‐stranded CMPs. The appropriate concentrations of **18‐Y** and **24‐Y** were calculated based on the triple helix/single strand ratio observed in the quantitative ^13^C NMR spectra (Figure 3b,c and Calculation S1). For equal concentrations of single‐stranded CMPs (0.42 mM) in both samples, a 0.43 mM solution of **18‐Y** was compared with a 4 mM solution of **24‐Y** (Figure 4). The ^13^C NMR spectrum of a 0.43 mM solution of ^13^C‐labeled **18‐Y** confirmed the calculated near‐exclusive presence of single‐stranded CMP (99%) at this concentration (Figure S6).

**FIGURE 4:**
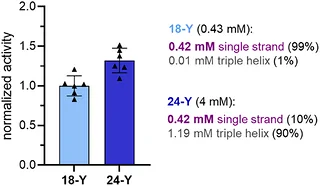
LOXL2‐catalyzed oxidation of equal concentrations of single‐stranded CMPs **18‐Y** and **24‐Y**. Oxidation rates of *n* = 6 measurements were normalized to **18‐Y** and are presented as mean ± SD; mean normalized activity of **24‐Y** = 1.32. Concentrations: *c*(LOXL2) = 50 nM = 4.3 µg/mL, *c*(**24‐Y**) = 4 mM, *c*(**18‐Y**) = 0.43 mM.

Amplex Red assays with these samples revealed comparable LOXL2 activity toward **18‐Y** and **24‐Y** (Figures 4 and S7 and Table S4). The slightly higher activity seen with **24‐Y** aligns with the dynamic equilibrium in which the triple‐helical pool (90%) continuously replenishes Lys‐containing single‐stranded CMPs. This finding implies that LOXL2 oxidizes Lys residues in single‐stranded but not triple‐helical CMPs. LOXL2‐catalyzed allysine formation is, therefore, unlikely in healthy triple‐helical collagen but feasible in Pro‐rich single strands of frayed collagen triple helices.

## 3 Conclusions

Herein, we revealed that human LOXL2, an enzyme important for ECM maturation and associated with disease states, oxidizes Lys residues in Pro‐rich single‐stranded collagen peptides. The examined CMPs have a high propensity to adopt PPII helices, but kinks are possible. This finding shows that LOXL2 activity is not limited to Lys residues in unstructured terminal telopeptides of natural collagens. Our studies also suggest that LOXL2 cannot oxidize Lys residues in triple‐helical collagen. The results imply that LOXL2 does not act on the triple‐helical collagen in healthy tissue, but on single‐stranded collagen in frayed regions of diseased tissue. The extension of LOXL2 activity from telopeptides to Pro‐rich peptides underscores the relevance of LOXL2 in collagen‐related diseases. Our data suggest that Lys oxidation and cross‐linking of pathologically disrupted collagen may play a greater role than so far appreciated. Combined with concomitantly generated H_2_O_2_, LOXL2‐induced Lys oxidation in disrupted collagen strands could, for example, contribute to excessive cross‐linking and matrix stiffening, a typical phenotype of many fibrotic states. These fundamental insights deepen our understanding of pathological collagen cross‐linking and raise intriguing perspectives for the design of LOXL2‐targeting tools for the diagnosis and therapy of connective tissue diseases [42, 43, 44].

## Author Contributions

**Laura M. Poller**: investigation, writing – original draft, conceptualization, methodology, validation, visualization, writing – review and editing, formal analysis, data curation. **Tomas Fiala**: writing – review and editing, formal analysis, validation. **Helma Wennemers**: conceptualization, funding acquisition, writing – review and editing, visualization, methodology, project administration, supervision, resources.

## Conflicts of Interest

The authors declare no conflicts of interest.

## Supporting information




**Supporting File**: The authors have cited additional references within the Supporting Information [45, 46].

## Data Availability

The data that support the findings of this study are available in the Supporting Information of this article.
