# Supplementary material for: Lysine Oxidation by LOXL2 in Single Strands Versus Triple Helices: Implications for Collagen‐Related Diseases
Source: Angew Chem Int Ed Engl. 2026 Aug 28;65(43):e1329257. doi: 10.1002/anie.1329257 (PMC13676411; doi:10.1002/anie.1329257)
Supplement: Supplementary file 1 [file ANIE-65-e1329257-s001.pdf]

Supporting Information

Lysine Oxidation by LOXL2 in Single Strands versus Triple Helices: Implications for Collagen-Related Diseases

Laura M. Poller,[a] Tomas Fiala,[a,b] Helma Wennemers[a]*

[a] Laboratory of Organic Chemistry, ETH Zurich, D-CHAB, Vladimir-Prelog-Weg 3, 8093 Zurich, Switzerland

[b] Department of Chemistry, Faculty of Science, Masaryk University, Kamenice 5, 625 00 Brno, Czech Republic

Email: helma.wennemers@org.chem.ethz.ch

Table of Contents

1. Figures and tables referenced in the main text S3
2. Synthesis and analytical data of peptides and building blocks S10
 2.1 General methods and materials S10
 2.2 Synthesis of Fmoc-^{13}C-1-Gly-OH S12
 2.3 General strategy for the synthesis of peptides S12
 2.4 Synthesis and analytical data of peptides S15
  Fmoc-GPO(tBu)-OH S15
  Ac-$(GPO)_2$GKP$(GPO)_3$-NH_2 (**18-X**) S16
  Ac-$(GPO)_3$GKP$(GPO)_3$-NH_2 (**21-X**) S17
  Ac-$(GPO)_4$GKP$(GPO)_3$-NH_2 (**24-X**) S18
  Ac-$(GPO)_2$GPK$(GPO)_3$-NH_2 (**18-Y**) S19
  Ac-$(GPO)_3$GPK$(GPO)_3$-NH_2 (**21-Y**) S20
  Ac-$(GPO)_4$GPK$(GPO)_3$-NH_2 (**24-Y**) S21
  Ac-$(GPO)_2$GPKGPO^{13}GPOGPO-NH_2 (**18-Y**; ^{13}C-label on Gly-13) S22
  Ac-$(GPO)_4$GPKGPO^{13}GPOGPO-NH_2 (**24-Y**; ^{13}C-label on Gly-19) S23
  Ac-$(POG)_6$-NH_2 ($(POG)_6$) S25
3. CD spectroscopy S26
 3.1 General procedure of CD measurements S26
 3.2 CD spectra in PBS, pH 7.4 ..... S26
 3.3 CD spectra in 50 mM sodium borate containing 0.1% BSA, pH 8.5 ..... S27
 3.4 General procedure of thermal denaturation studies S28
 3.5 Thermal denaturation studies in PBS, pH 7.4 ..... S28
 3.6 Thermal denaturation studies in 50 mM sodium borate containing 0.1% BSA, pH 8.5 29
4. NMR spectroscopy S30
 4.1 General procedure of NMR measurements S30
 4.2 Fmoc-^{13}C-1-Gly S30
 4.3 Ac-$(GPO)_2$GPKGPO^{13}GPOGPO-NH_2 (**18-Y**; ^{13}C-label on Gly-13) S31
  18-Y (4 mM) S31
  18-Y (0.43 mM) S33
 4.4 Ac-$(GPO)_4$GPKGPO^{13}GPOGPO-NH_2 (**24-Y**; ^{13}C-label on Gly-19) S33
5. HRMS spectrum of the aldehyde product of **18-Y** S34
6. References S35

1. Figures and tables referenced in the main text

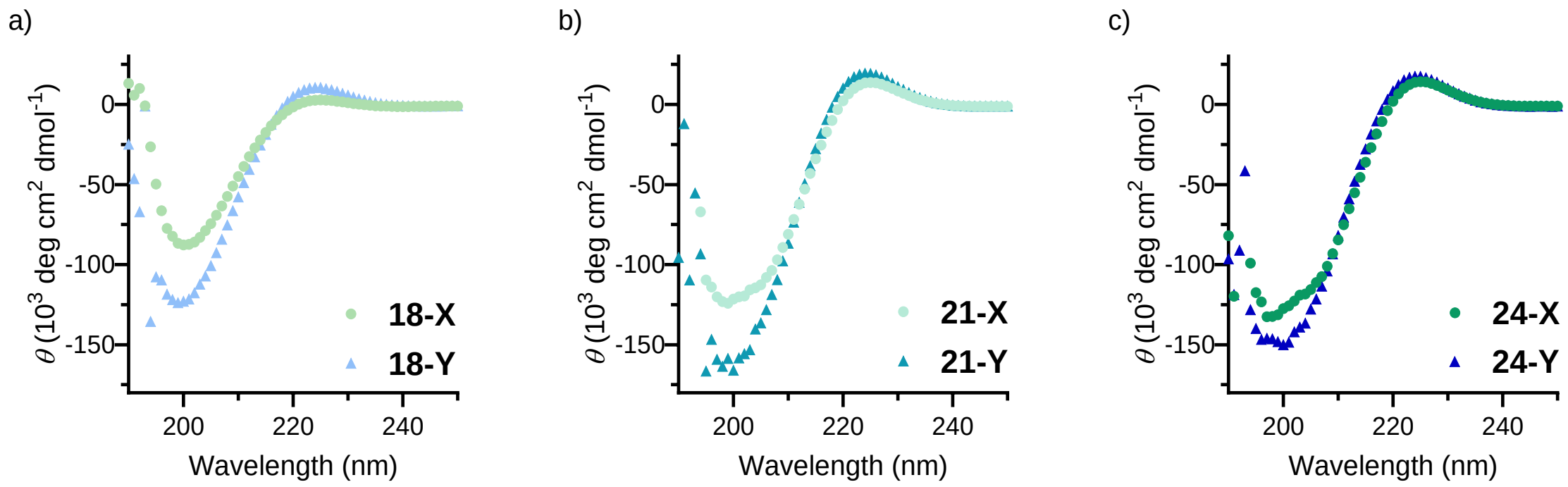

Figure S1 Representative circular dichroism (CD) spectra of a) 18-mer, b) 21-mer, and c) 24-mer CMPs with Lys either at the Xaa or Yaa position. The characteristic maxima at 225 nm and minima close to 200 nm indicate a polyproline II (PPII)-helical structure. CD spectra were recorded using solutions of the CMPs (200 µM) in phosphate-buffered saline (PBS), pH 7.4, at 7 °C; *n* = 2 independent experiments.

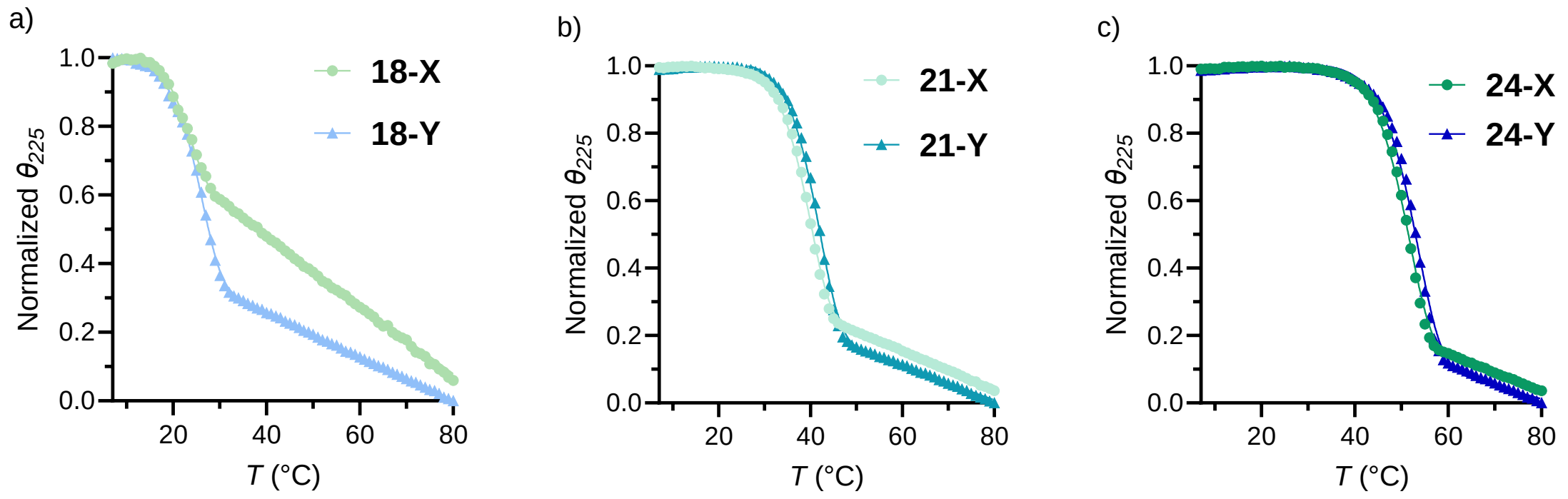

Figure S2 Triple helix stability of a) 18-mer, b) 21-mer, and c) 24-mer CMPs with Lys either at the Xaa or Yaa position was determined by thermal denaturation measurements at 225 nm. Spectra were recorded using solutions of the CMPs (200 µM) in PBS, pH 7.4, at a heating rate of 1 °C/114 s; n = 2 independent experiments.

Table S1 CMPs of different lengths with Lys either at the Xaa or Yaa position and T_m values of the respective triple helices in PBS, pH 7.4.

**Code**	**Sequence**	**T_m (°C)**
**(POG)₆**	Ac-(POG)$_6$-NH$_2$	28.3 ± 0.3
**18-X**	Ac-[GPO]$_2$-G**K**P-[GPO]$_3$-NH$_2$	21.7 ± 0.9
**18-Y**	Ac-[GPO]$_2$-GP**K**-[GPO]$_3$-NH$_2$	24.7 ± 0.1
**21-X**	Ac-[GPO]$_3$-G**K**P-[GPO]$_3$-NH$_2$	38.3 ± 0.2
**21-Y**	Ac-[GPO]$_3$-GP**K**-[GPO]$_3$-NH$_2$	40.3 ± 0.1
**24-X**	Ac-[GPO]$_4$-G**K**P-[GPO]$_3$-NH$_2$	50.0 ± 0.2
**24-Y**	Ac-[GPO]$_4$-GP**K**-[GPO]$_3$-NH$_2$	51.9 ± 0.1

T_m values of CMP solutions (200 µM) were determined by thermal denaturation with CD spectroscopic monitoring at 225 nm and a heating rate of 1 °C/114 s; n = 2 independent experiments; values are presented as mean ± standard deviation (SD).

LOX activity was measured by the Amplex Red assay in sodium borate buffer containing 0.1% bovine serum albumin (BSA) at pH 8.5. Under these buffer conditions, the triple helices formed by CMPs **24-X** and **24-Y** have T_m values comparable to those in PBS.

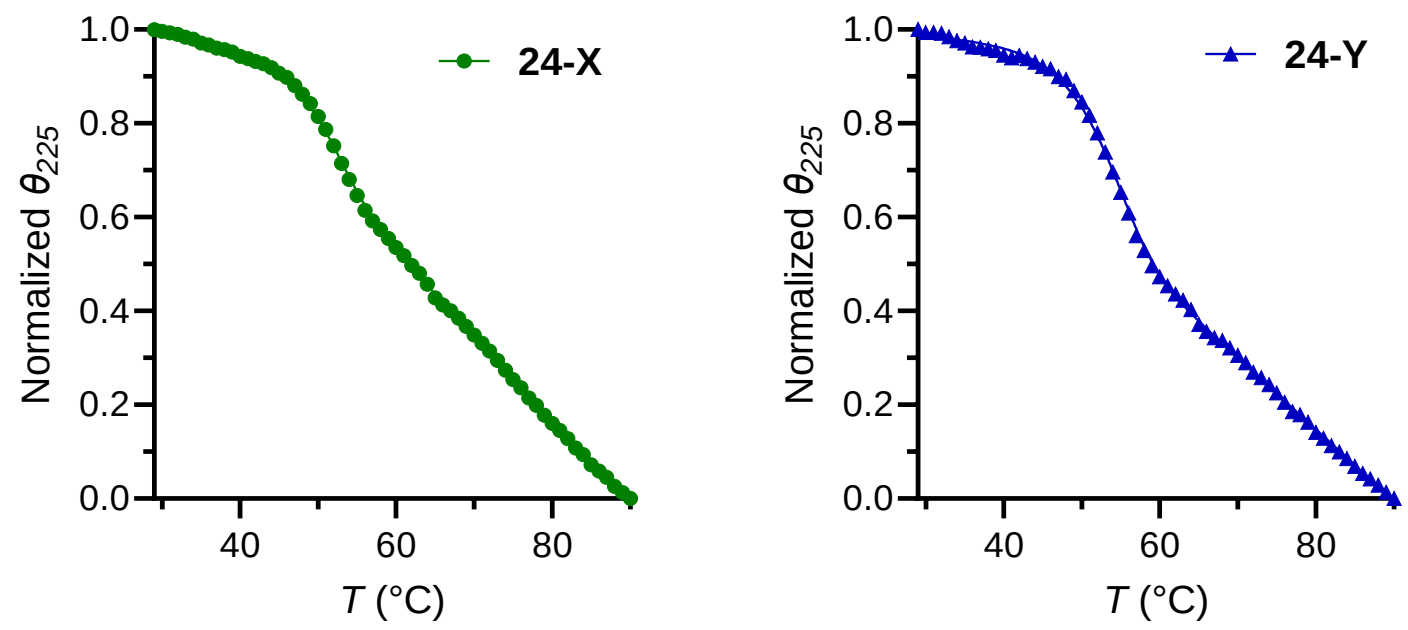

Figure S3 Triple helix stability of **24-X** and **24-Y** was determined by thermal denaturation measurements at 225 nm. Spectra were recorded using solutions of the CMPs (200 μM) in sodium borate containing 0.1% BSA, pH 8.5, at a heating rate of 1 °C/114 s; n = 2 independent experiments.

Table S2 T_m values of **24-X** and **24-Y** triple helices in 50 mM sodium borate containing 0.1% BSA, pH 8.5.

Code	CMP	T_m (°C)
**24-X**	Ac-[GPO]$_4$-G**K**P-[GPO]$_3$-NH$_2$	49.8 ± 4.9
**24-Y**	Ac-[GPO]$_4$-GP**K**-[GPO]$_3$-NH$_2$	52.4 ± 6.0

T_m values of CMP solutions (200 μM) were determined by thermal denaturation with CD spectroscopic monitoring at 225 nm and a heating rate of 1 °C/114 s; n = 2 independent experiments; values are presented as mean ± SD.

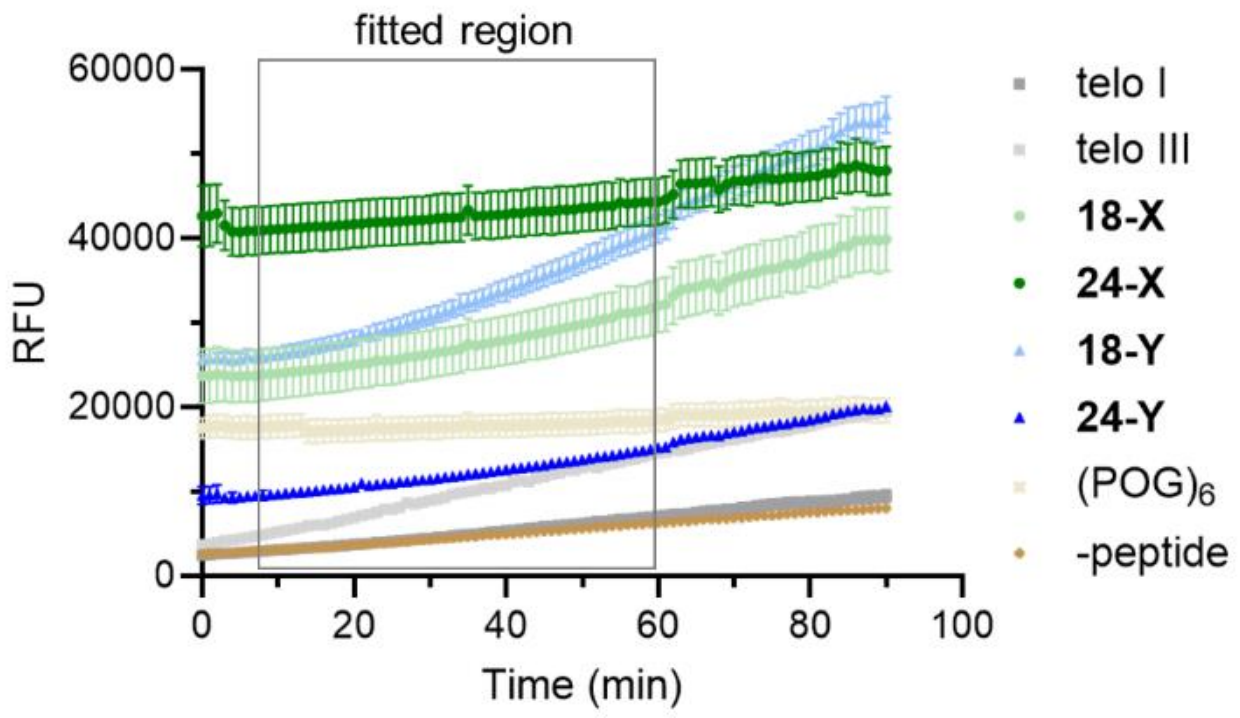

Figure S4 Kinetic measurements show LOXL2 activity toward CMPs of varying lengths with Lys either at the Xaa (green) or Yaa position (blue), as well as Lys in C-terminal telopeptides of collagen I and III (telo I and telo III). $(POG)_6$, lacking a Lys, served as a negative control; the experiment in the absence of a peptide ("-peptide") provided the background signal. Representative raw data are presented as mean ± standard deviation (SD) of n = 2-3 independent experiments, each performed in technical triplicate. Concentrations: *c*(LOXL2) = 50 nM = 4.3 µg/mL, *c*(peptides) = 4 mM, *c*(HRP) = 0.1 U/mL, *c*(Amplex Red) = 120 µM.

Table S3 Normalized LOXL2 activity toward Lys either at the Xaa or Yaa position in CMPs, as well as telo I and telo III, within 1 h.

telo I	telo III	$(POG)_6$	**18-X**	**24-X**	**18-Y**	**24-Y**
0.24 ± 0.03	1.00 ± 0.01	-0.04 ± 0.04	1.46 ± 0.08	0.37 ± 0.02	3.0 ± 0.2	0.85 ± 0.07

LOXL2 activity, displayed in Figure 2b and S4, was determined from the linear slope within 1 h (indicated fitted region in Figure S4). For enhanced comparison, activity was normalized to telo III. Representative data are presented as mean ± SD of n = 2-3 independent experiments, each performed in technical triplicate. $(POG)_6$, lacking a Lys, served as a negative control.

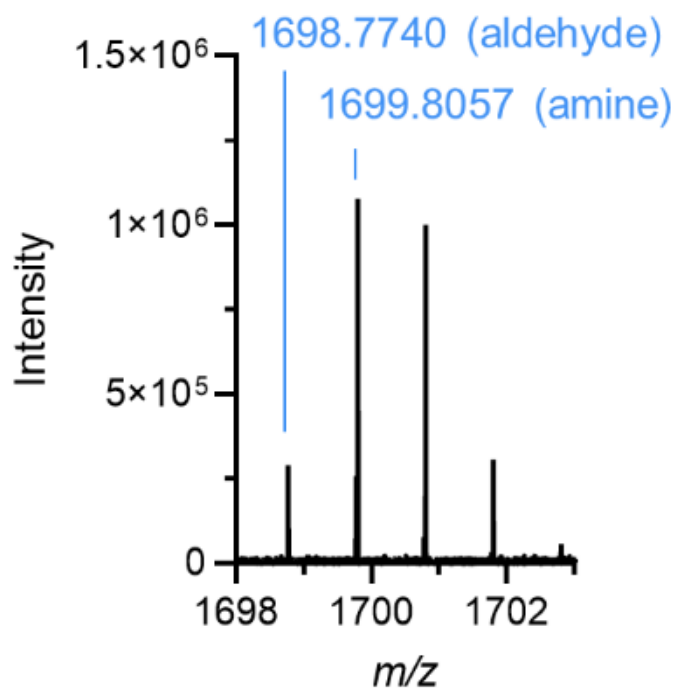

Figure S5 Following incubation of **18-Y** and LOXL2, high-resolution mass spectrometry (HRMS) detected the sodium adduct ($[M+Na]^+$) of the allysine product (*m/z* = 1698.7740 Da; *calc. m/z* = 1698.7734 Da), generated from **18-Y** (*m/z* = 1699.8057 Da; *calc. m/z* = 1699.8051 Da). Concentrations during incubation: *c*(LOXL2) = 1 µM = 86 µg/mL, *c*(peptide) = 0.1 mM.

Calculation S1: **18-Y** concentration that equals the single strand concentration in 4 mM **24-Y**

To distinguish between Lys oxidation in Pro-rich single-stranded versus triple-helical CMPs, we compared LOXL2 activity in samples of **18-Y** and **24-Y** with equal concentrations of single-stranded CMPs. The appropriate concentrations of **18-Y** and **24-Y** were calculated based on the triple helix/single strand ratio observed in the quantitative ^{13}C NMR spectra (Figures 3b and 3c). To calculate the thermodynamic equilibrium at lower CMP concentrations, Cardano's method was used to solve the cubic equation; abbreviations: single strand, SS; triple helix, TH; *c*, concentration.

Equilibrium: $3\,\text{SS} \rightleftharpoons \text{TH}$ (1)

Equilibrium constant $K = \frac{c(\text{TH})}{c(\text{SS})^3}$ (2)

Ratio at 4 mM, determined by ^{13}C NMR spectroscopy (Figure 3b): 64% SS 36% TH

Concentrations in equilibrium:

$c(\text{SS}) = 0.64 \times 4\text{ mM} = 2.56\text{ mM SS}$

$c(\text{TH}) = (0.36 \times 4\text{ mM}) / 3 = 0.48\text{ mM TH}$

→ Equation (2): Equilibrium constant $K = \frac{c(\text{TH})}{c(\text{SS})^3} = 28610\text{ M}^{-2}$

Cubic equation for calculating SS concentration:

$$c(\text{SS})^3 + \frac{c(\text{SS})}{3K} - \frac{total\ conc.}{3K} = 0 \quad (3)$$

with $\frac{1}{3K} = 1.16 \times 10^{-5}\text{ M}^2$

Solving cubic equation (3) according to Cardano's method:

$$c(SS) = \sqrt[3]{-\frac{1}{2}\left(-\frac{total\ conc.}{3K}\right) + \sqrt[2]{D}} + \sqrt[3]{-\frac{1}{2}\left(-\frac{total\ conc.}{3K}\right) - \sqrt[2]{D}^{\frac{1}{3}}} \quad (4)$$

using Discriminant $D = \frac{1}{2^2}\left(-\frac{total\ conc.}{3K}\right)^2 + \frac{1}{3^3}\left(\frac{1}{3K}\right)^3$ (5)

SS concentration in a 0.43 mM solution of **18-Y**:

$-\frac{total\ conc.}{3K} = -5.01 \times 10^{-9}\text{ M}^3$ → Equation (5): Discriminant $D = 6.48 \times 10^{-17}$

→ Equation (4): $c(\text{SS}) = 0.42\text{ mM}$

Calculated ratio at 0.43 mM: 98.5% SS 1.5% TH

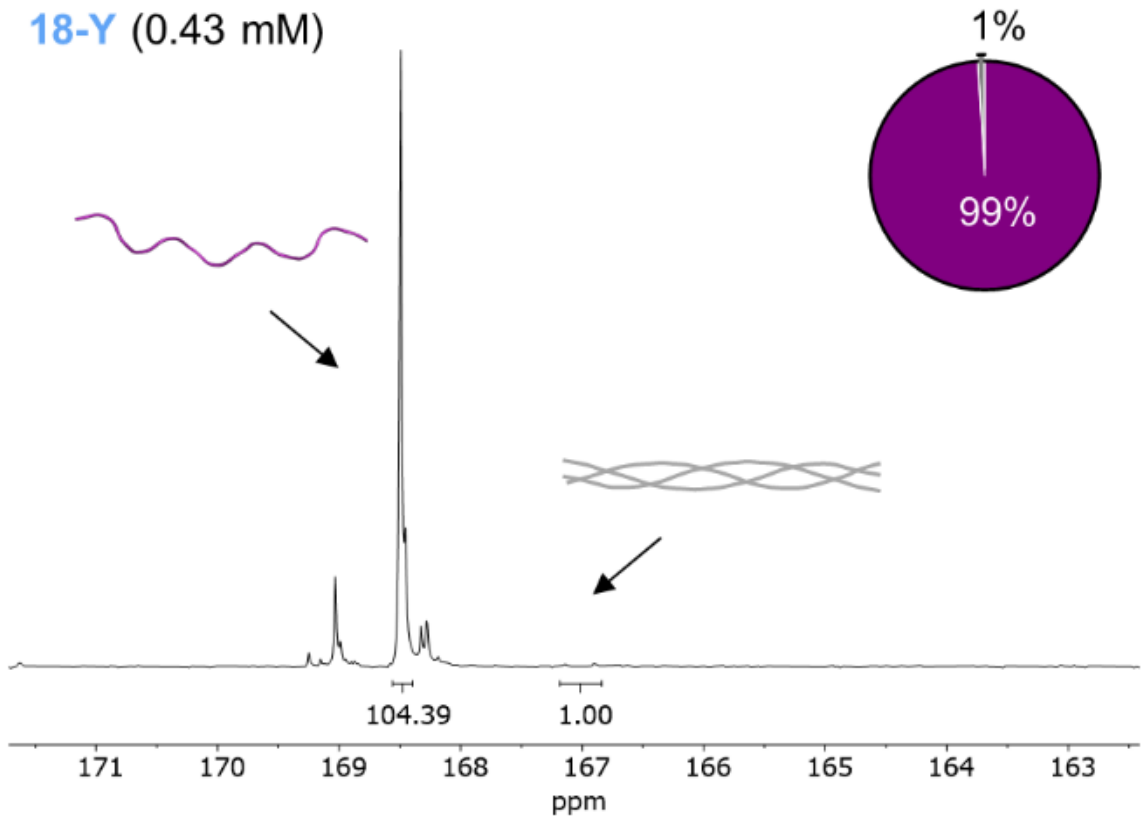

Figure S6 The quantitative ^{13}C NMR spectrum of ^{13}C-labeled **18-Y** (0.43 mM) shows the near-exclusive presence of single-stranded (δ = 168.5 ppm) to triple-helical (δ = 167.0 ppm) CMPs. The spectrum of **18-Y** was recorded in assay buffer (50 mM sodium borate containing 0.1% BSA, pH 8.5) at 29 °C after annealing overnight.

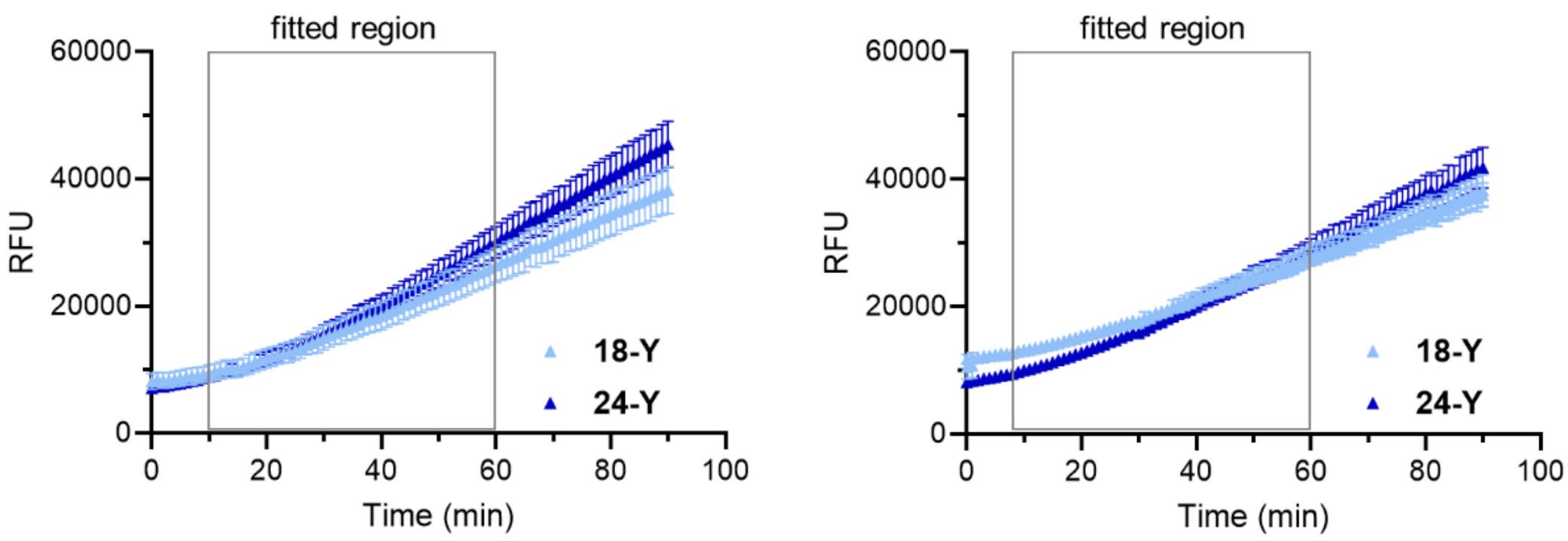

Figure S7 Kinetic measurements show LOXL2 activity in solutions of **18-Y** and **24-Y**, containing equimolar single-stranded CMPs. Raw data are presented as mean ± SD of *n* = 2 independent experiments, each performed in technical triplicate. Concentrations: *c*(LOXL2) = 50 nM = 4.3 µg/mL, *c*(**24-Y**) = 4 mM, *c*(**18-Y**) = 0.43 mM, *c*(HRP) = 0.1 U/mL, *c*(Amplex Red) = 120 µM.

Table S4 Normalized LOXL2 activity toward Lys in **18-Y** and **24-Y**, containing equimolar amounts of single-stranded CMPs, within 1 h.

**18-Y**	**24-Y**
1.00 ± 0.13	1.32 ± 0.16

LOXL2 activity, displayed in Figure 4 and S7, was determined from the linear slope within 1 h (indicated fitted region in Figure S7). For enhanced comparison, activity was normalized to **18-Y** and is presented as mean ± SD of *n* = 6 measurements.

2. Synthesis and analytical data of peptides and building blocks

2.1 General methods and materials

Materials and reagents were purchased from Abcam, Acros Organics, Abcr, Sigma Aldrich, Thermo Fisher Scientific, Bachem, Apollo Scientific, Fluorochem, Senn, and TCI. Chemicals purchased at the highest available purity were used without further purification. Milli-Q water with a resistivity of 18.2 MΩ.cm, prepared by a Sartorius Arium611VF water purification system, was used for peptide preparation and purification.

Solid phase peptide synthesis (SPPS) was performed on a Liberty Blue synthesizer (CEM, Matthews, NC, USA) on a 50 µmol scale, using the Fmoc/tBu strategy with a Rink amide ChemMatrix resin (Biotage, Uppsala, Sweden; loading 0.42 mmol/g, 100–200 mesh) or a 2-chlorotritylchloride resin (Abcr). Manual SPPS was carried out in a Biotage syringe with a Teflon frit (Biotage PP-Reactor 10 mL or 50 mL with PTFE frit). Peptides were dried by lyophilization on a Christ Alpha 2–4 LD plus lyophilizer (Kuhner AG).

Preparative reverse-phase HPLC was performed on a Waters HPLC consisting of a Waters 2555 Quaternary Gradient Module, a Waters 2489 UV/Vis Detector, and a Waters 2767 Sample Manager (Waters Corporation, Milford, MA, USA), equipped with a ReproSil Gold C18 column (250 × 10 mm, 10 µm particle size, 120 Å pore size). Milli-Q water containing 1% acetonitrile and 0.1% trifluoroacetic acid (TFA) (solvent A) and acetonitrile (solvent B) were used as eluents at a flow rate of 20 mL/min and an oven temperature of 50 °C. After purification, all collected fractions were analyzed by analytical HPLC or LC-MS. Only fractions with purity >97% were combined.

Analytical reverse-phase HPLC was performed using a Dionex UHPLC, Ultimate 3000 from Thermo Fisher Scientific (Waltham, MA, USA) equipped with a Jupiter C18 column (250 × 4.6 mm, 5 µm particle size, 300 Å pore size) from Phenomenex (column 1) or a Nucleodur C18 Gravity-SB (250 × 4.6 mm, 5 µm particle size, 110 Å pore size) from Macherey-Nagel (column 2). Acetonitrile (solvent A) and water containing 1% acetonitrile and 0.1% TFA (solvent B) were used as eluents at a flow rate of 1 mL/min and an oven temperature of 65 °C (unless specified otherwise).

High-resolution mass spectrometry (MS) was performed by the Molecular and Biomolecular Analysis (MoBiAs) service of the D-CHAB at ETH Zurich, using a Bruker Daltons maXis equipped with an ESI (electrospray ionization) source and a Q-TOF ion analyzer, or a Bruker Daltonics solariX equipped with a MALDI–ESI source and a Q-TOF ion analyzer. α-Cyano-4-hydroxycinnamic (CHCA) was used as MALDI–MS matrix.

Circular dichroism (CD) spectra were recorded on a Chirascan Plus spectrometer (Applied Photophysics Ltd, Leatherhead, UK) with a Nitropack nitrogen generator (Parker Balston) and a

temperature controller TC 125 (Quantum Northwest). The solutions were measured in a quartz cuvette with a path length of 1.0 mm (Hellma 110-QS, Hellma Analytics). The lyophilized CMPs were dissolved in PBS, pH 7.4 (gibco, 10010-015), or 50 mM sodium borate containing 0.1% BSA, pH 8.5, at a concentration of 200 µM. The solutions were subsequently annealed by heating to 80 °C for 15 min in a water bath, cooling to room temperature or 29 °C, and storage at 4 °C or 29 °C overnight prior to CD spectroscopic analysis and thermal denaturation.

LOXL2 activity was measured in 50 mM sodium borate containing 0.1% BSA at pH 8.5 by monitoring the formation of H_2O_2 through the Amplex Red assay.[1–3] The peptides were dissolved in 50 mM sodium borate containing 0.1% BSA, pH 8.5, providing solutions with a 3-fold increased concentration as in the subsequent oxidation assay. The CMP solutions were annealed as described for the CD measurements (*vide supra*) and stored at 29 °C overnight. The reaction mixtures (100 µL), containing horseradish peroxidase (HRP, 0.1 U/mL, Abcr), Amplex Red (120 µM, Abcam), recombinant LOXL2 (50 nM = 4.3 µg/mL, ACROBiosystems, UniProt Q9Y4K0; batches A1293b-210920F1-BULK (lot#1293B-219MF1-2437) and A1293b-250507F1-Bulk (lot#1293b-2557F1-2614); stored at -80 °C), and substrate (0.43 mM or 4 mM) were prepared in a 96-well plate with a round bottom to yield the concentrations indicated in brackets. Aliquots of the mixture were transferred (30 µL) into a black 384-well plate with a flat, transparent bottom (Greiner) in triplicate to give three technical replicates. The relative fluorescence units (RFU) were measured at 29 °C every minute for at least 1 h using excitation at 550 nm and emission at 595 nm on a Tecan Instrument Spark 10M Multiplate Reader (Serial Nr.: 1609002590). The oxidation rates were calculated from the linear slope via simple linear regression using GraphPad Prism 9 (GraphPad Software, San Diego, CA, USA). Relative activities were normalized to a standard (*L*-lysine), present in all experiments; the background (absence of a peptide; -peptide) was subtracted, and the data were normalized to telo III (Figure 2b) or **18-Y** (Figure 4) for enhanced comparison.

Mass of the aldehyde-containing CMP product was measured by HRMS (*vide supra*), following incubation of the CMP (0.1 mM) with recombinant LOXL2 (1 µM = 86 µg/mL) in 50 mM sodium borate containing 0.1% BSA (pH 8.5; assay buffer) at 29 °C overnight.

^{13}C nuclear magnetic resonance (NMR) spectra were measured by the NMR service of the Laboratory of Organic Chemistry at the ETH Zurich on a Bruker AV III 600 (600/150MHz). The lyophilized ^{13}C-labeled CMPs were dissolved in 50 mM sodium borate containing 0.1% BSA (pH 8.5; assay buffer) at a 3-3.75-fold higher concentration than used in the measurement. The solutions were annealed, as described for the CD measurements (*vide supra*), followed by storage at 29 °C overnight. The solutions were then diluted with assay buffer and D_2O to the desired concentration (4 mM or 0.43 mM). Spectra of the solutions, containing 12% D_2O, were recorded at 29 °C, and NMR data were analyzed with MestReNova.

2.2 Synthesis of Fmoc-^{13}C-1-Gly-OH

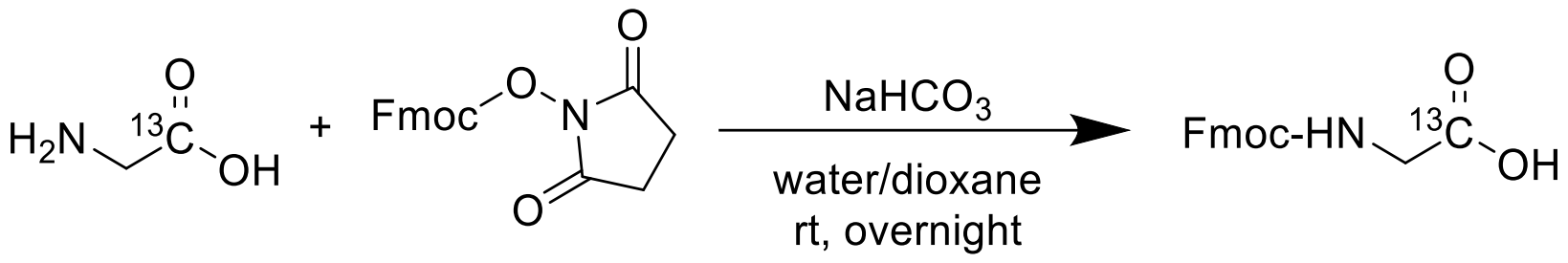

An aqueous solution (2.2 mL, 1.5 M, pH 8-9) of $NaHCO_3$ (276 mg, 3.3 mmol, 2.5 equiv) was slowly added to ^{13}C-1-Gly (100.00 mg, 1.31 mmol), which was accompanied by the evolution of gas in the colorless solution. A solution of Fmoc-succinimide (444 mg, 1.3 mmol, 1.0 equiv) in dioxane (1.1 mL, 1.2 M) was slowly added, and the white, cloudy reaction mixture was stirred at rt overnight. The organic solvent was removed, and a solution of saturated aqueous $NaHCO_3$ (3 mL) was added to reach pH 8-9. The white, milky mixture was washed with Et_2O (3 x 10 mL) and acidified with 3 M HCl to pH 1. The white suspension was extracted with EtOAc (4 x 20 mL), dried over Na_2SO_4, and the solvent was removed under vacuo. The Fmoc-protected ^{13}C-1-Gly was obtained as a white solid (192.3 mg, 0.645 mmol, 49%).

^{1}H NMR (400 MHz, MeOD) δ 7.84 – 7.76 (m, 2H), 7.71 – 7.63 (m, 2H), 7.43 – 7.33 (m, 2H), 7.31 (td, J = 7.5, 1.2 Hz, 2H), 4.34 (d, J = 7.2 Hz, 2H), 4.23 (t, J = 7.1 Hz, 1H), 3.84 (d, J = 5.6 Hz, 2H).
^{13}C NMR (101 MHz, MeOD) δ 173.6, 159.1, 145.3, 142.6, 128.8, 128.2, 126.3, 120.9, 68.2, 43.4, 42.8.
HRMS (ESI): calc. *m/z* for $C_{16}H_{15}NNaO_4{}^{13}C$ $[M+Na]^+$ = 321.0927, found 321.0925.

2.3 General strategy for the synthesis of peptides

Protocol A – Swelling of Rink amide resin

Before peptide synthesis, the resin (loading 0.42 mmol/g) was swollen in a fritted syringe in CH_2Cl_2 for 20 min while shaking. The solvent was drained from the resin, and the resin was washed with DMF (3×).

Protocol B – Swelling of 2-chlorotritylchloride resin

Before peptide synthesis, the resin (loading 1.6 mmol/g) was dried in a fritted syringe under high vacuum for 1.5 h, set under N_2 atmosphere, and swollen in anhydrous CH_2Cl_2 for 40 min while shaking. Then the solvent was drained from the resin.

Protocol C – Microwave-assisted automated peptide synthesis

Microwave-assisted automated peptide synthesis was performed on a Liberty Blue synthesizer (CEM, Matthews, NC, USA) on a 50 µmol or 100 µmol scale. A sequence of deprotection–wash–coupling–wash steps was employed using 20% piperidine in DMF for Fmoc deprotection and Fmoc-AA-OH in DMF (AA = Gly, Pro or Hyp(tBu); 6 equiv, 0.2 M stock), HATU in DMF (6 equiv, 0.5 M stock), and $^{i}Pr_2NEt$ in NMP (12 equiv, 2 M stock) for amino acid couplings, and DMF for washing. The general sequence of steps employed is shown in the table below. Coupling was performed under microwave heating at 75 °C,

and coupling times were gradually increased from 5 min up to 13 min as the peptide length increased. The resin-bound peptide was obtained with an N-terminal Fmoc-protecting group.

No	Step	Reagent	Total Vol. (mL)	Time (min)
**1**	wash	DMF	4	-†
**2**	deprotection	20% piperidine	3	1
**3**	deprotection	20% piperidine	3	4
**4**	deprotection	20% piperidine	3	4
**5**	wash	DMF	8	-†
**6**	wash	DMF	4	-†
**7**	wash*	DMF	4	-†
**8**	wash	DMF	4	-†
**9**	wash	DMF	4	-†
**10**	wash	DMF	4	-†
**11**	wash	DMF	4	-†
**12**	coupling	Fmoc-AA-OH, HATU, DIPEA	4	5-13
**13**	wash*	DMF	4	-†
**14**	wash*	DMF	8	-†
**15**	wash	DMF	4	-†
**16**	wash	DMF	4	-†
**17**	wash	DMF	4	-†
**18**	wash	DMF	4	-†
**19**	wash	DMF	4	-†

*The "wash through manifold" option was used.

†The addition of wash solvent was followed by N_2 bubbling through the suspension for 4 s and draining.

Protocol D – Manual coupling of Fmoc-Hyp(tBu)-OH

Fmoc-Hyp(tBu)-OH (1.25 equiv) was dried under high vacuum for 3.5 h, dissolved in anhydrous CH_2Cl_2 (15 mL) and diisopropylethylamine (^{i}Pr$_2$NEt, Hünig's base) (2.5 equiv) under N_2 atmosphere, and gently vortexed under N_2 atmosphere. The solution was added to the resin and agitated for 4 h. MeOH (0.8 mL) was added, and the mixture was shaken for 10 min. The resin was drained, washed with anhydrous DMF (15 mL), drained, and MeOH (20 mL) was added. The mixture was shaken for 1 min, followed by washing with DMF for 1 min (5×).

Protocol E – Manual coupling of Fmoc-Gly-OH, Fmoc-Pro-OH, and Fmoc-Gly(^{13}C)-OH

The resin-bound peptide was washed with DMF for 1 min (5×). Fmoc-AA-OH (2.1 equiv) and HATU (2.0 equiv) were dissolved in DMF (1.5 mL), followed by the addition of ^{i}Pr$_2$NEt (4.0 equiv). After 1 min of

preactivation, the solution was added to the resin-bound peptide. The mixture was agitated for 1 h (Fmoc-Pro-OH), 1.5 h (Fmoc-Gly-OH), or 2 h (Fmoc-Gly(^{13}C)-OH), followed by washing with DMF for 1 min (5×).

Protocol F – Manual coupling of Fmoc-GPO(tBu)-OH

The resin-bound peptide was washed with DMF for 1 min (5×). Fmoc-GPO(tBu)-OH (1.5 equiv) and HATU (1.45 equiv or 3.0 equiv) were dissolved in DMF (1.5 mL), followed by the addition of $^{i}Pr_2NEt$ (3.0 equiv or 6.0 equiv). After 1 min of preactivation, the solution was added to the resin-bound peptide. The mixture was agitated for 3 h, followed by washing with DMF for 1 min (5×). For the synthesis of ^{13}C-labeled **24-Y**, 3.1 equiv of Fmoc-GPO(tBu)-OH was used.

Protocol G – Manual Fmoc deprotection

The resin-bound peptide was agitated with 20% piperidine in DMF (2.5 mL) for 3 min (3×), followed by washing with DMF for 1 min (5×).

Protocol H – N-terminal acetyl capping

The resin-bound peptide was washed with CH_2Cl_2 for 1 min (3×). Acetic anhydride (250 µL) was dissolved in CH_2Cl_2 (2.0 mL), followed by the addition of $^{i}Pr_2NEt$ (250 µL). The resulting solution was added to the resin-bound peptide and agitated for 15 min, followed by washing with CH_2Cl_2 for 1 min (5×).

Protocol I – Cleavage off the Rink amide resin

The resin was treated with a mixture of TFA/H_2O/triisopropyl silane (TIS) (95:2.5:2.5; 5.0 mL) for 60 min and filtered. This procedure was repeated with the same cleavage solution (2.5 mL) for 30 min, and the combined filtrates were concentrated under a stream of N_2. The peptide was precipitated by the addition of cold Et_2O (6 mL). After centrifugation and decantation of the supernatant, the off-white solid was air-dried, dissolved in a mixture of water and acetonitrile (1:1; 10 mL), and lyophilized to obtain a white foam.

Protocol J – Cleavage off 2-chlorotrityl chloride resin

The resin was washed with CH_2Cl_2 for 1 min (5x), treated with 40% 1,1,1,3,3,3-hexafluoroisopropanol (HFIP) in CH_2Cl_2 (5 mL) for 1.5 h, filtered into a round-bottom flask, washed with CH_2Cl_2 for 1 min (2×), and filtered (2×). This procedure was repeated with the same cleavage solution (4 mL) for 1 h, and the combined filtrates were concentrated under reduced pressure (30 °C). The residual was dissolved in water (75 mL), a saturated aqueous solution of $NaHCO_3$ (25 mL) was added, and washed with Et_2O (3 × 50 mL). The aqueous phase was acidified to pH 1 using a 3 M HCl solution (30 mL) and extracted with ethyl acetate (3 × 65 mL). The organic phases were combined, dried over $MgSO_4$, filtered into a round-bottom flask, and concentrated under reduced pressure (30 °C). The substance was dissolved in a mixture of water and acetonitrile (1:1, 10 mL) and transferred into a Falcon tube. The round-bottom

flask was rinsed with a mixture of water and acetonitrile (1:1, 5 mL), water (5 mL), and acetonitrile (5 mL), and the solution was lyophilized to obtain a white foam.

2.4 Synthesis and analytical data of peptides

Fmoc-GPO(tBu)-OH

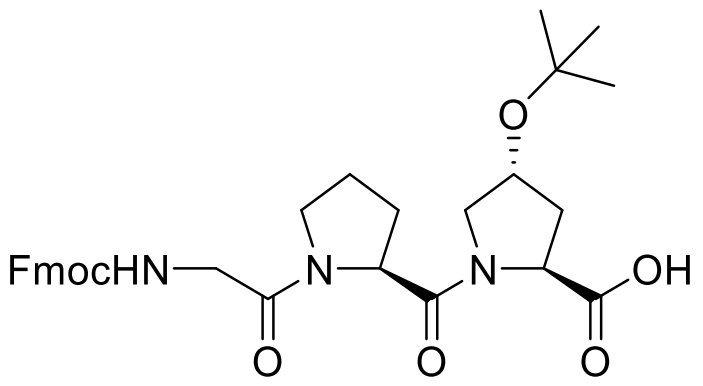

The synthesis was manually performed on a 6.4 mmol scale. The resin was swollen according to protocol B, Fmoc-Hyp(*t*Bu)-OH was coupled according to protocol D, N-terminal Fmoc was deprotected according to protocol G, Fmoc-Pro-OH was coupled according to protocol E, N-terminal Fmoc was deprotected according to protocol G, Fmoc-Gly-OH was coupled according to protocol E, and the peptide was cleaved from the resin according to protocol J.

HRMS (MALDI/ESI+): calc. *m/z* for $C_{31}H_{37}N_3NaO_7$ $[M+Na]^+$ = 586.2524, found: 586.2522.

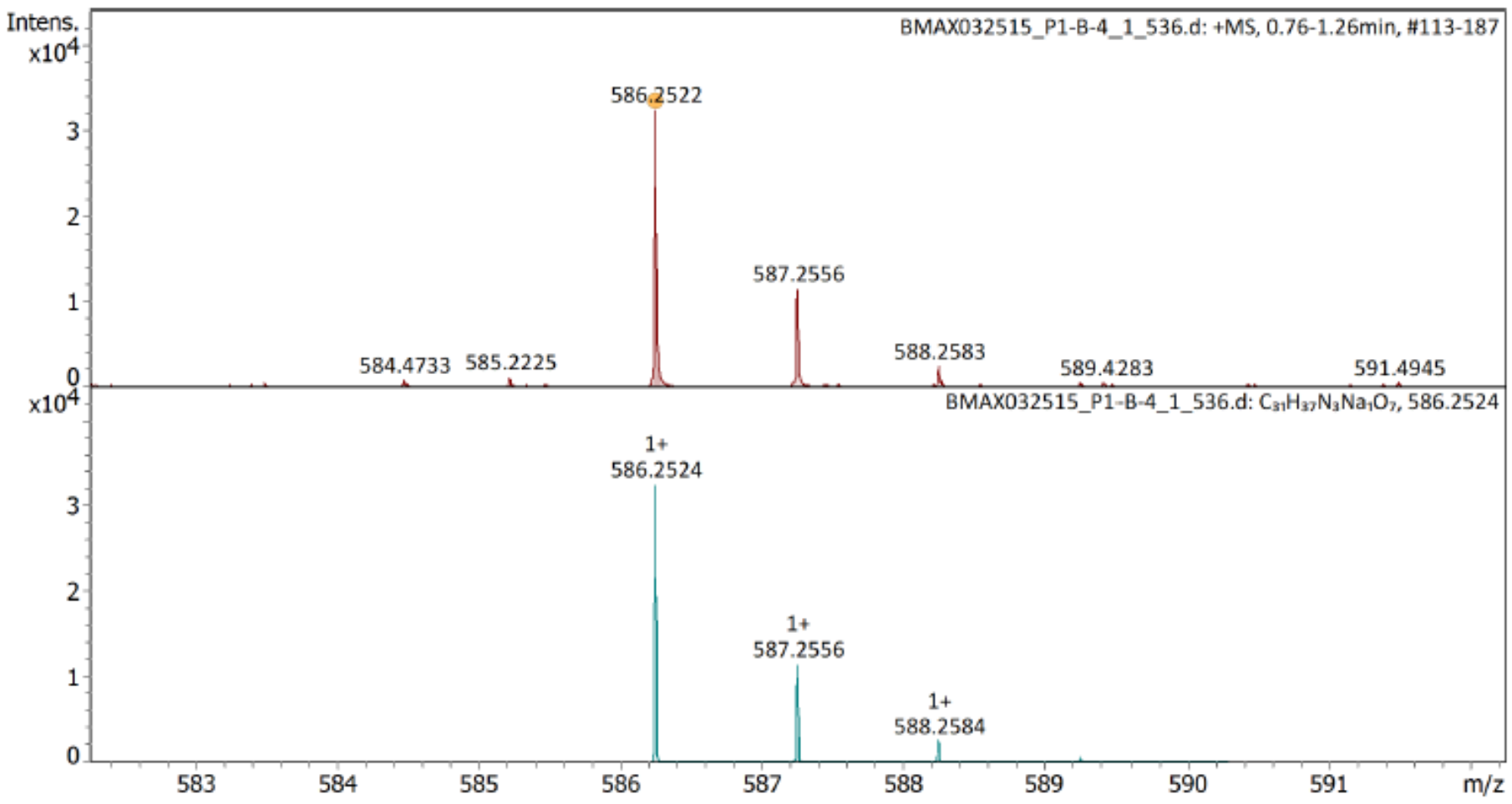

Ac-(GPO)$_2$GKP(GPO)$_3$-NH$_2$ (**18-X**)

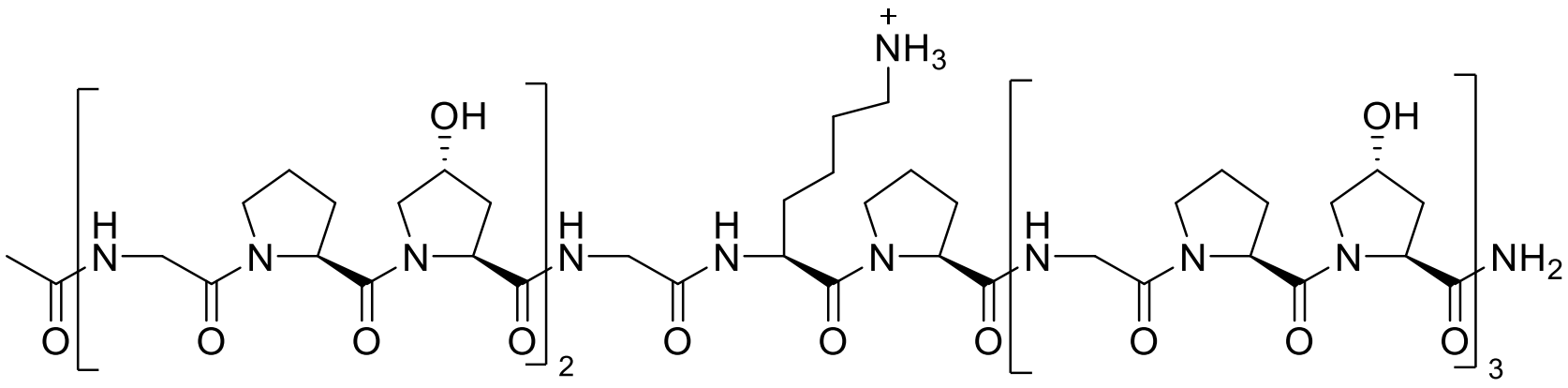

The resin was swollen according to protocol A, the peptide was synthesized by microwave-assisted automated synthesis according to protocol C, the N-terminal Fmoc was deprotected according to protocol G, the peptide was acetyl capped according to protocol H, and finally cleaved from the resin according to protocol I. The crude product was purified by preparative RP HPLC using a gradient of 96–90% solvent A over 30 min.

Purity (analytical HPLC, column 1, 50 °C): >97 %

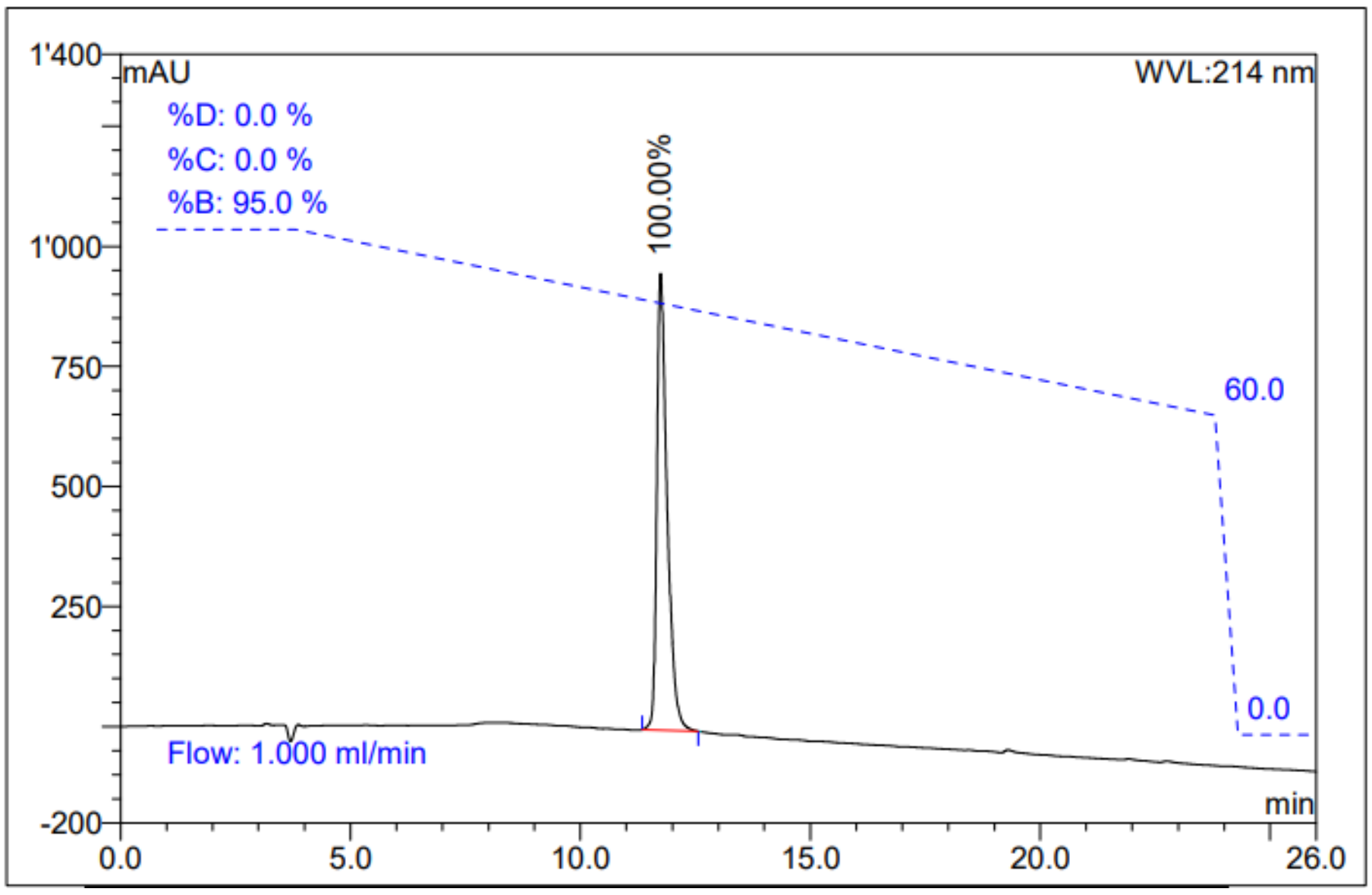

HRMS (MALDI/ESI+): calc. *m/z* for $C_{75}H_{113}N_{20}O_{24}$ $[M+H]^+$ = 1677.8231, found: 1677.8232.

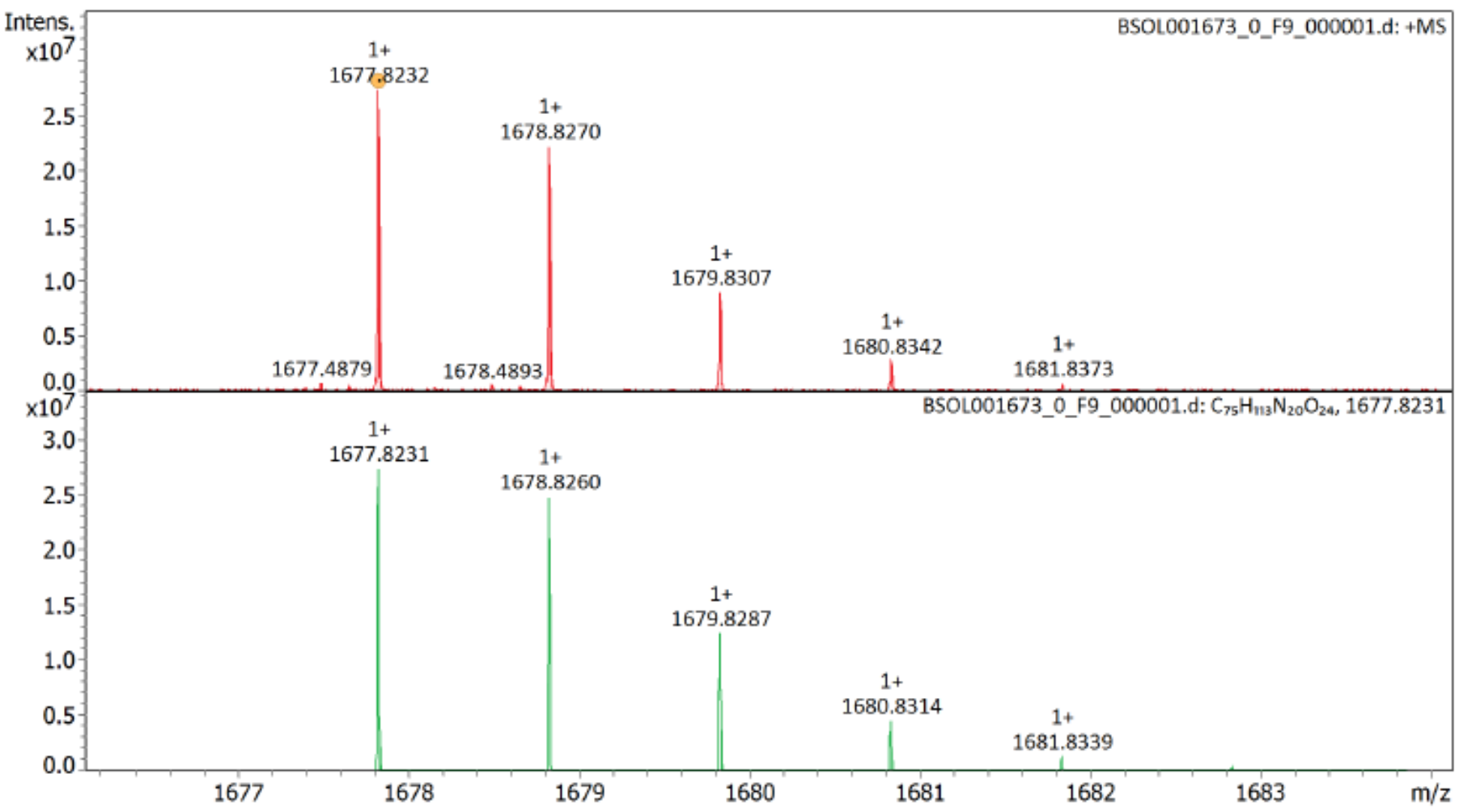

Ac-(GPO)$_3$GKP(GPO)$_3$-NH$_2$ (**21-X**)

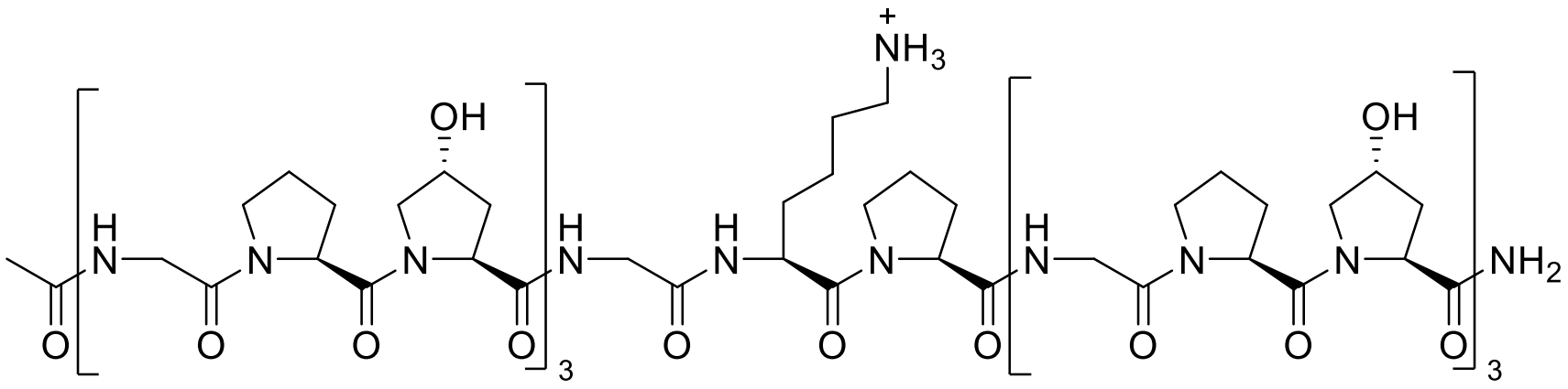

The resin was swollen according to protocol A, and the peptide was synthesized by microwave-assisted automated synthesis according to protocol C up to a length of 18 amino acids. After washing with DMF for 1 min (5×), Fmoc was deprotected according to protocol G, and Fmoc-GPO(tBu)-OH was manually coupled according to protocol F. N-terminal Fmoc was deprotected according to protocol G, the peptide was acetyl capped according to protocol H, and finally cleaved from the resin according to protocol I. The crude product was purified by preparative RP HPLC using a gradient of 97-89% solvent A over 30 min.

Purity (analytical HPLC, column 1, 65 °C): >97 %

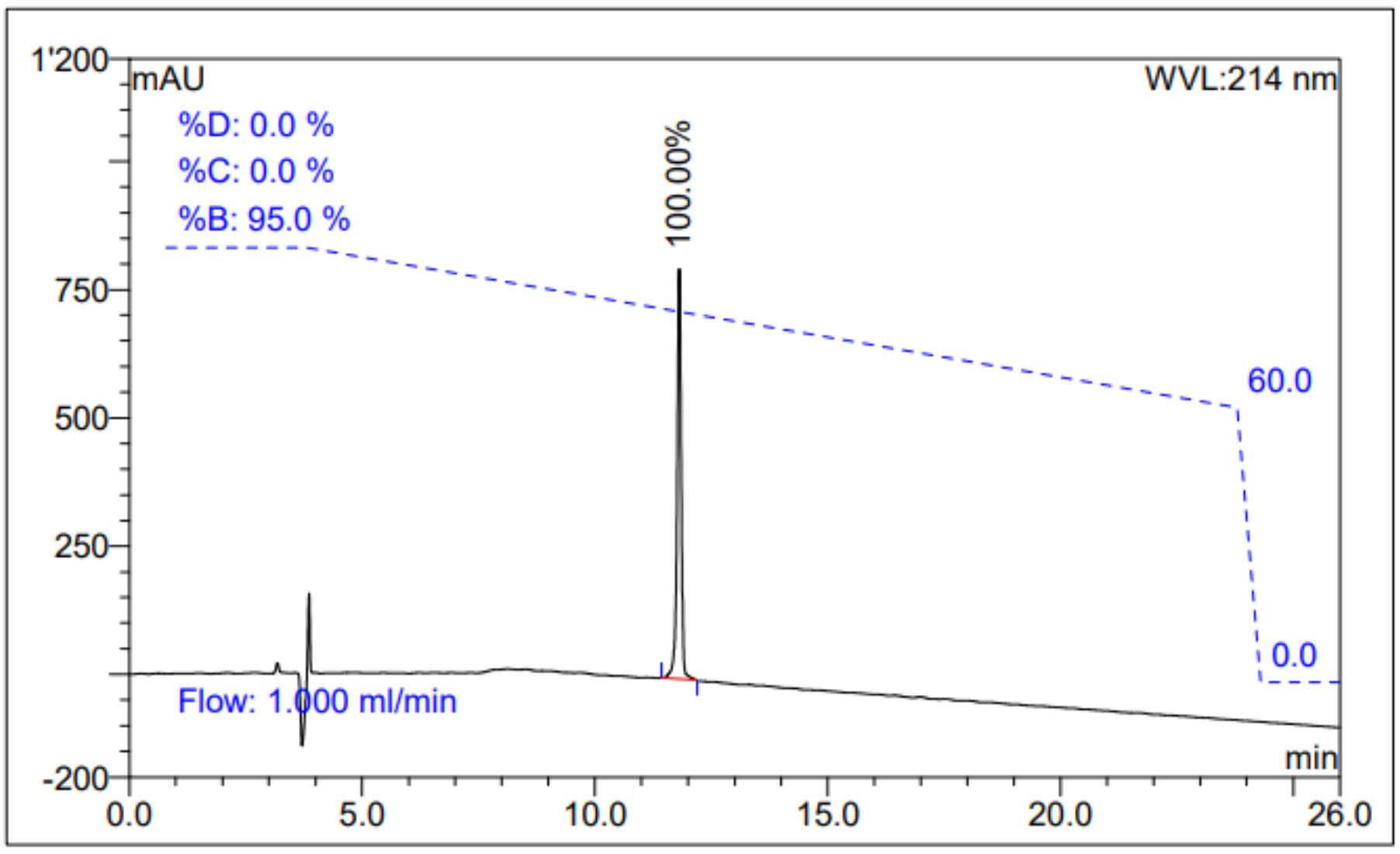

HRMS (MALDI/ESI+): calc. *m/z* for $C_{87}H_{130}N_{23}O_{28}$ [M+H]$^+$ = 1944.9450, found: 1944.9445.

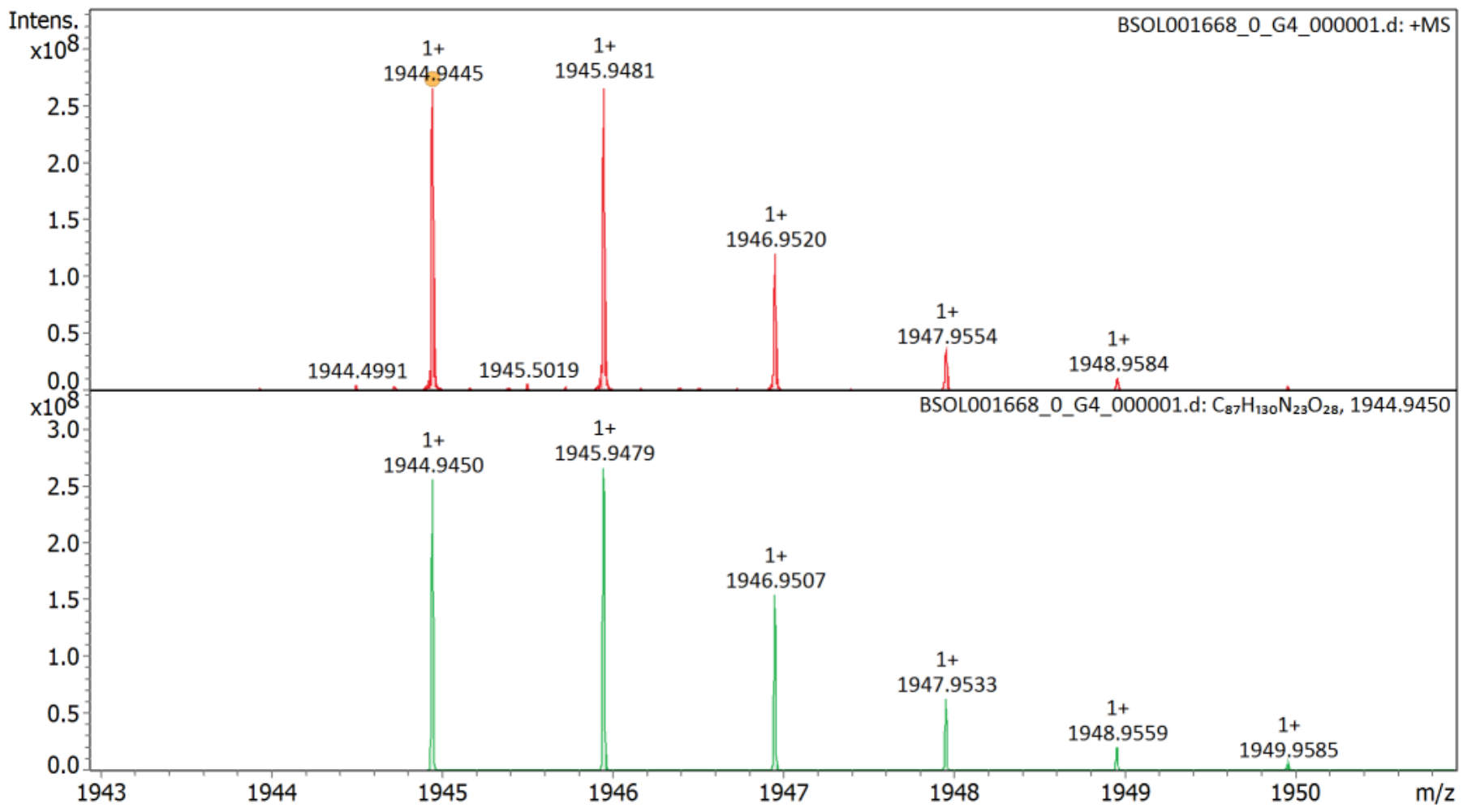

Ac-(GPO)4GKP(GPO)3-NH2 (**24-X**)

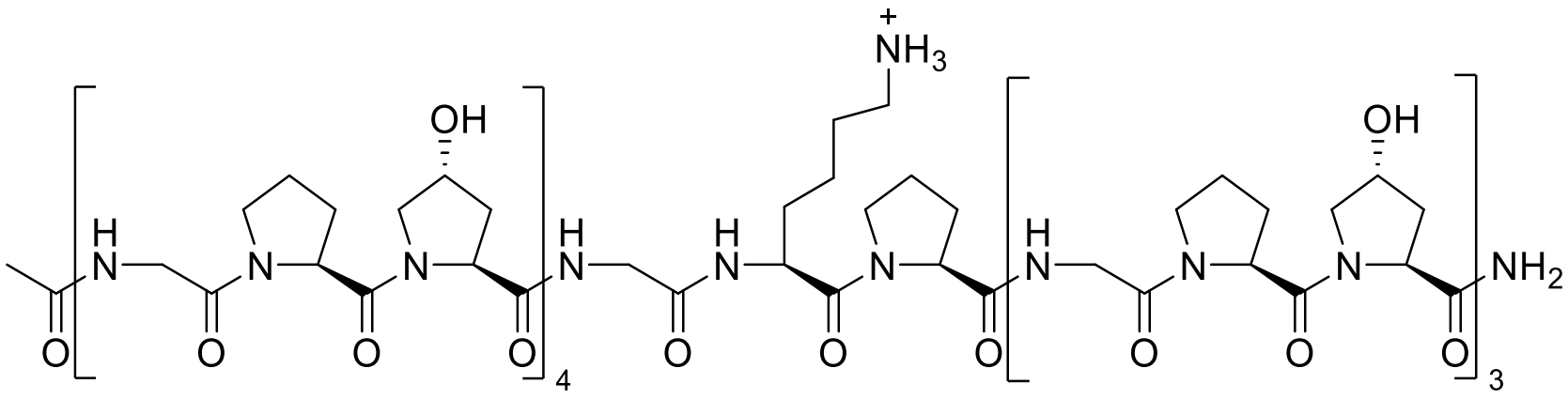

The resin was swollen according to protocol A, and the peptide was synthesized by microwave-assisted automated synthesis according to protocol C up to a length of 18 amino acids. After washing with DMF for 1 min (5×), Fmoc was deprotected according to protocol G, and Fmoc-GPO(tBu)-OH was manually coupled according to protocol F. Fmoc deprotection and manual coupling of Fmoc-GlyProHyp(tBu)-OH were performed once more. N-terminal Fmoc was deprotected according to protocol G, the peptide was acetyl capped according to protocol H, and finally cleaved from the resin according to protocol I. The crude product was purified by preparative RP HPLC using a gradient of 95-88% solvent A over 30 min.

Purity (analytical HPLC, column 1, 65 °C): 97%

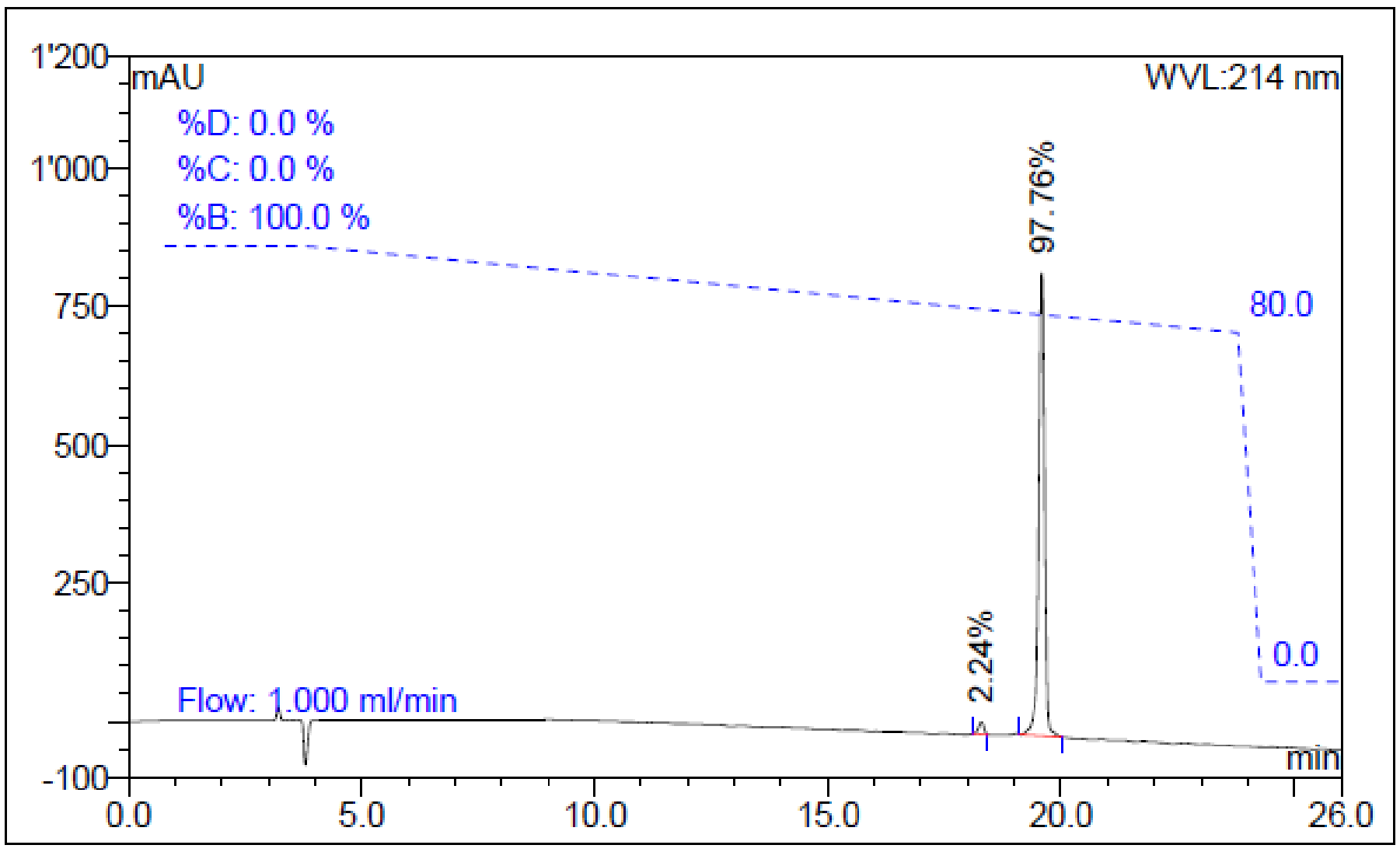

HRMS (MALDI/ESI+): calc. *m/z* for $C_{99}H_{147}N_{26}O_{32}$ $[M+H]^+$ = 2212.0669, found: 2212.0667.

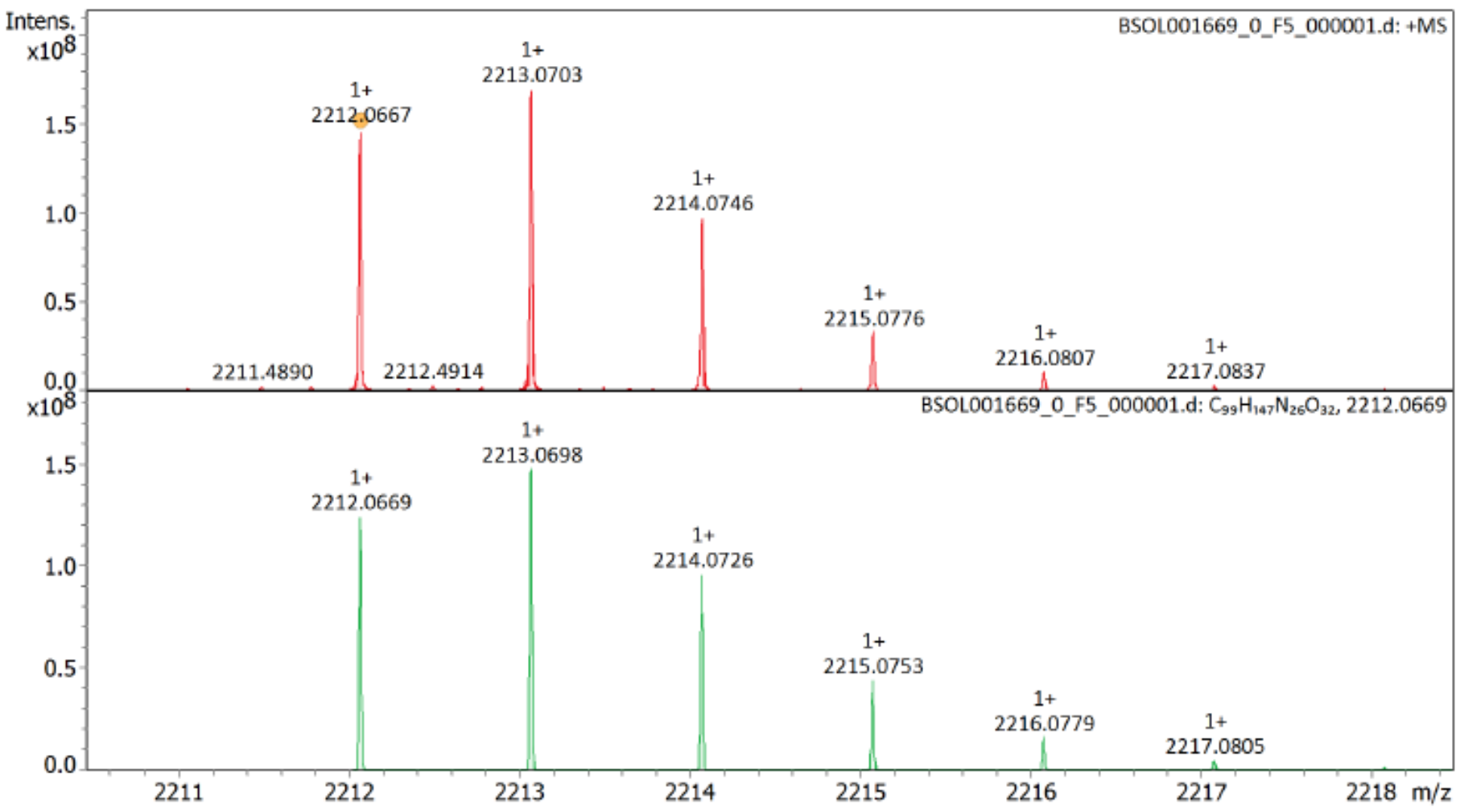

Ac-(GPO)$_2$GPK(GPO)$_3$-NH$_2$ (**18-Y**)

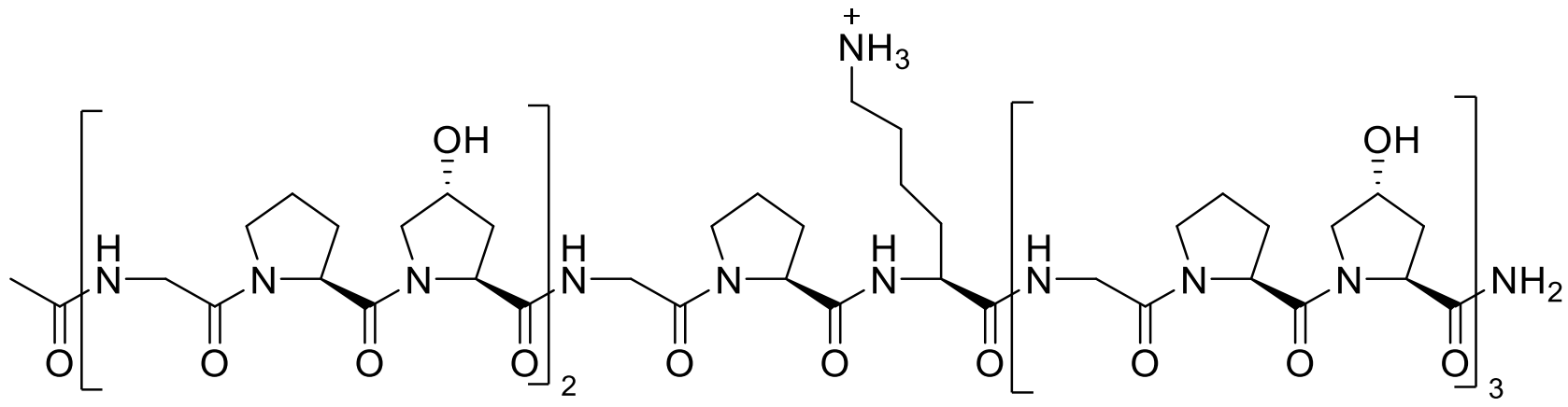

The resin was swollen according to protocol A, the peptide was synthesized by microwave-assisted automated synthesis according to protocol C, the N-terminal Fmoc was deprotected according to protocol G, the peptide was acetyl capped according to protocol H, and finally cleaved from the resin according to protocol I. The crude product was purified by preparative RP HPLC using a gradient of 95–90% solvent A over 30 min.

Purity (analytical HPLC, column 1, 65 °C): >97%

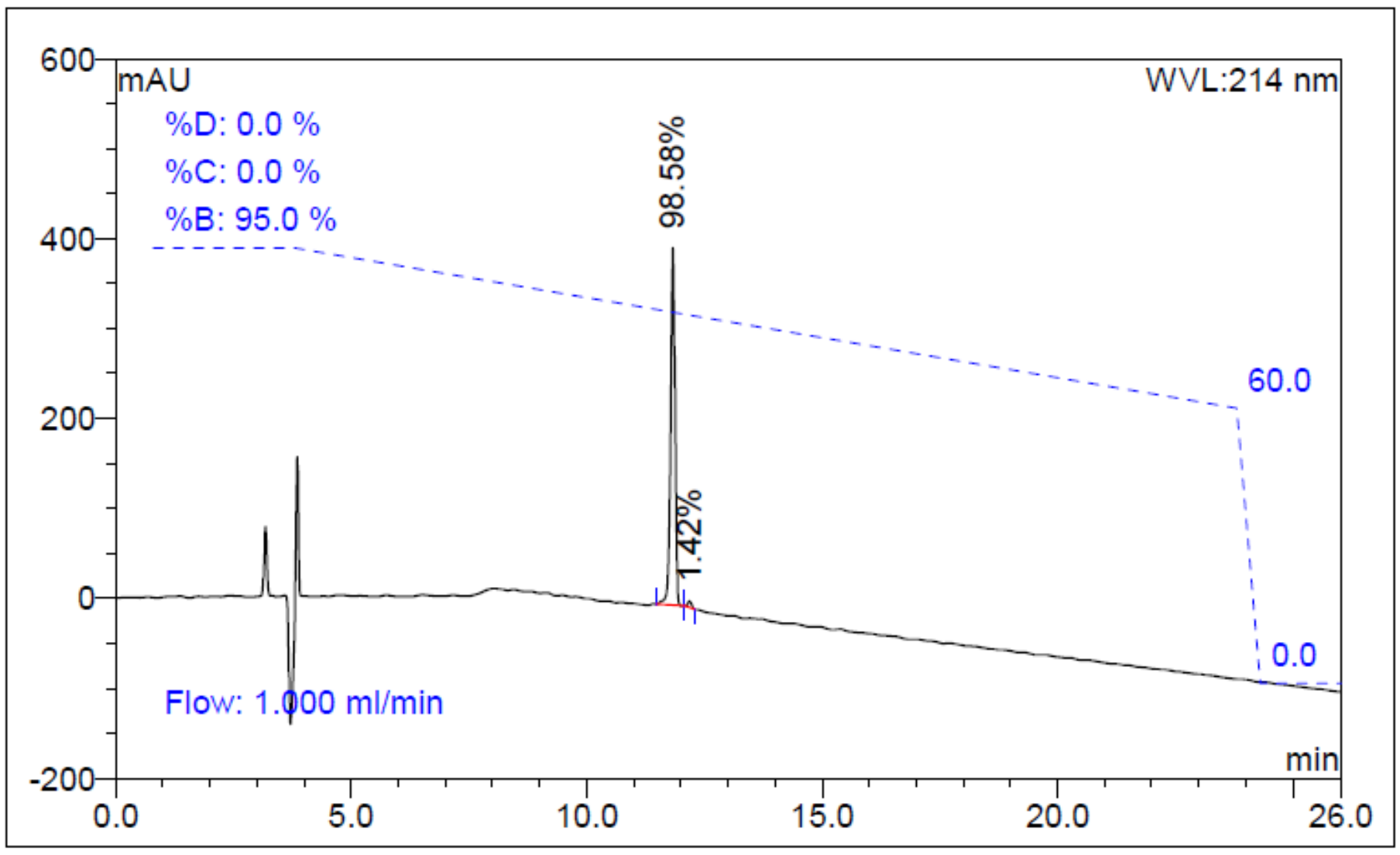

HRMS (MALDI/ESI+): calc. *m/z* for $C_{75}H_{113}N_{20}O_{24}$ $[M+H]^+$ = 1677.8231, found: 1677.8229.

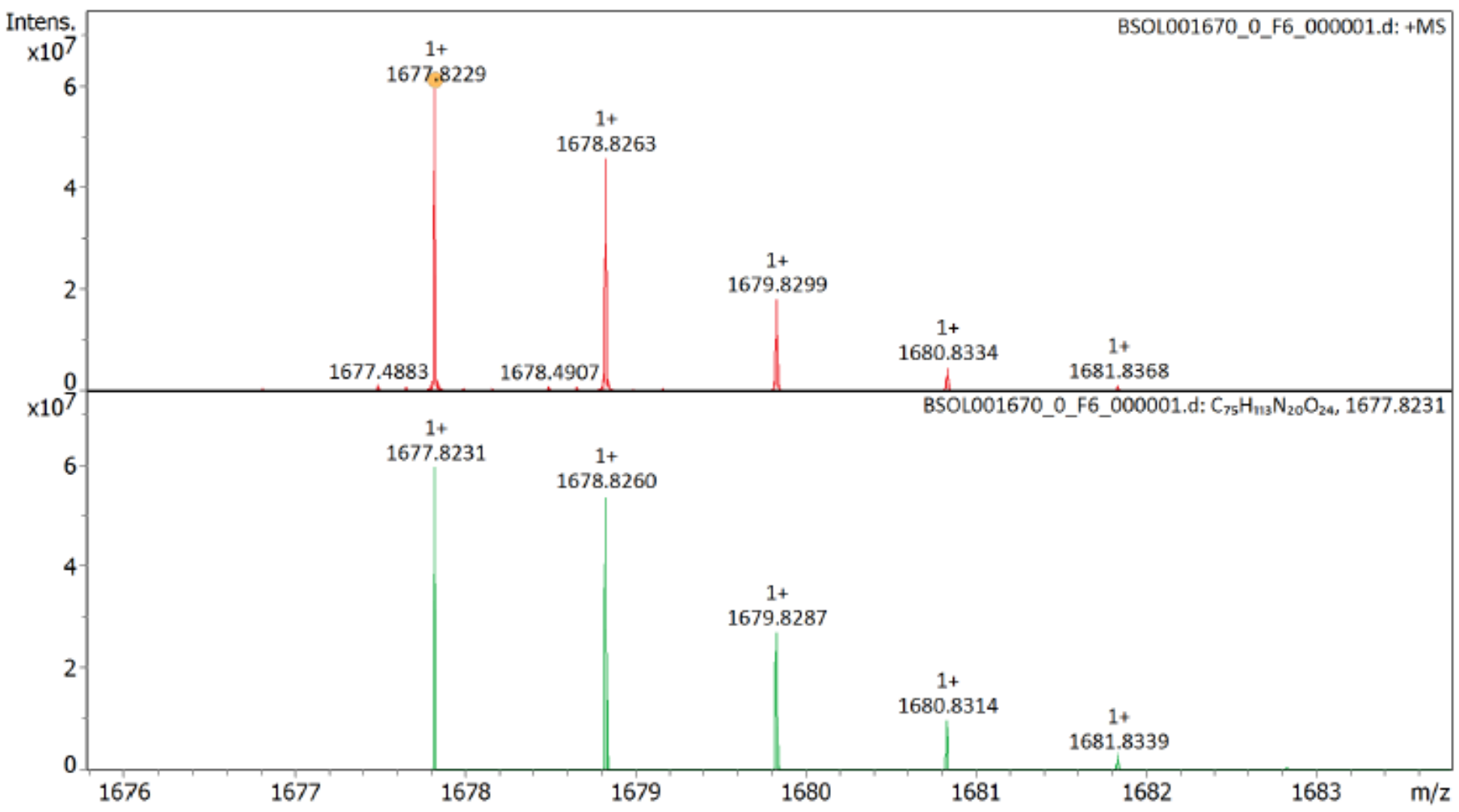

Ac-(GPO)$_3$GPK(GPO)$_3$-NH$_2$ (**21-Y**)

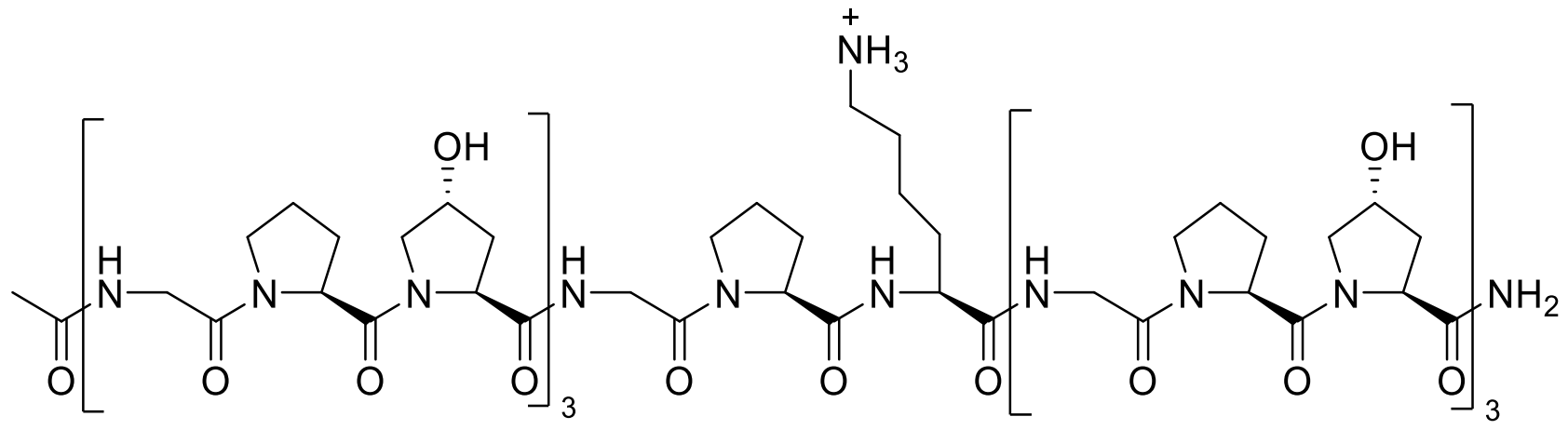

The resin was swollen according to protocol A, and the peptide was synthesized by microwave-assisted automated synthesis according to protocol C up to a length of 18 amino acids. After washing with DMF for 1 min (5×), Fmoc was deprotected according to protocol G, and Fmoc-GPO(tBu)-OH was manually coupled according to protocol F. N-terminal Fmoc was deprotected according to protocol G, the peptide was acetyl capped according to protocol H, and finally cleaved from the resin according to protocol I. The crude product was purified by preparative RP HPLC using a gradient of 96-88% solvent A over 30 min.

Purity (analytical HPLC, column 1, 65 °C): >97%

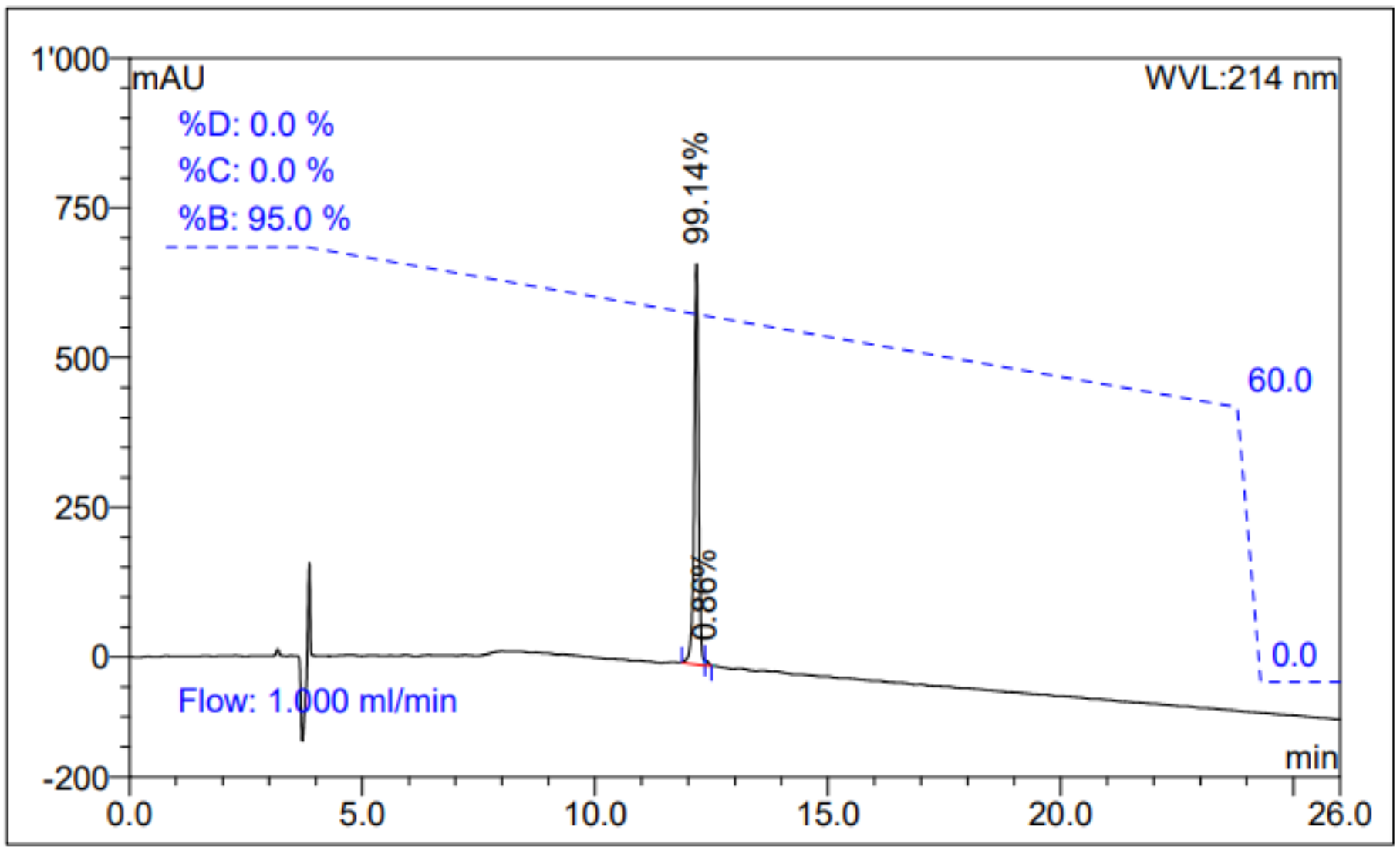

HRMS (MALDI/ESI+): calc. *m/z* for $C_{87}H_{130}N_{23}O_{28}$ [M+H]$^+$ = 1944.9450, found: 1944.9445.

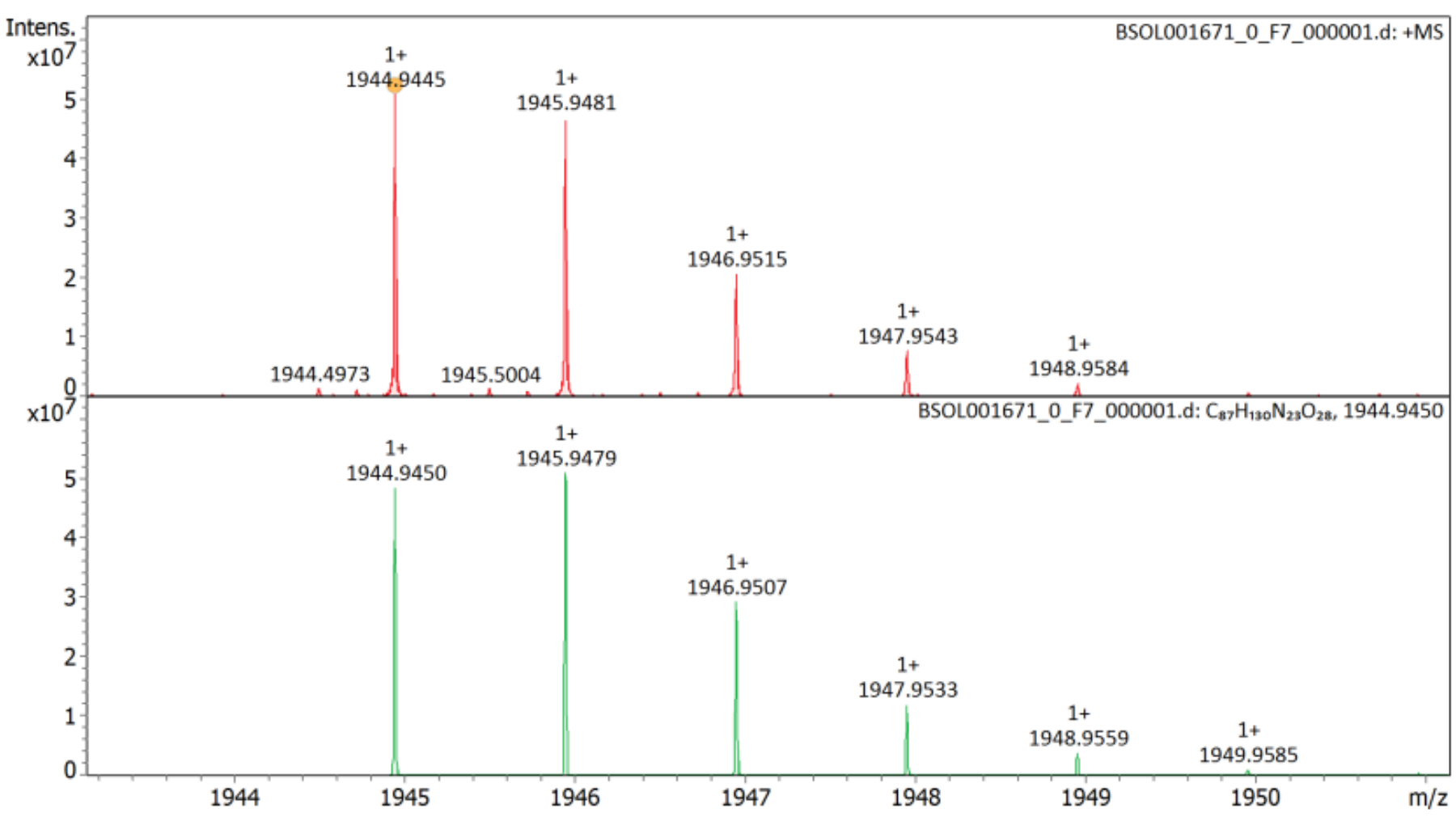

Ac-(GPO)$_4$GPK(GPO)$_3$-NH$_2$ (**24-Y**)

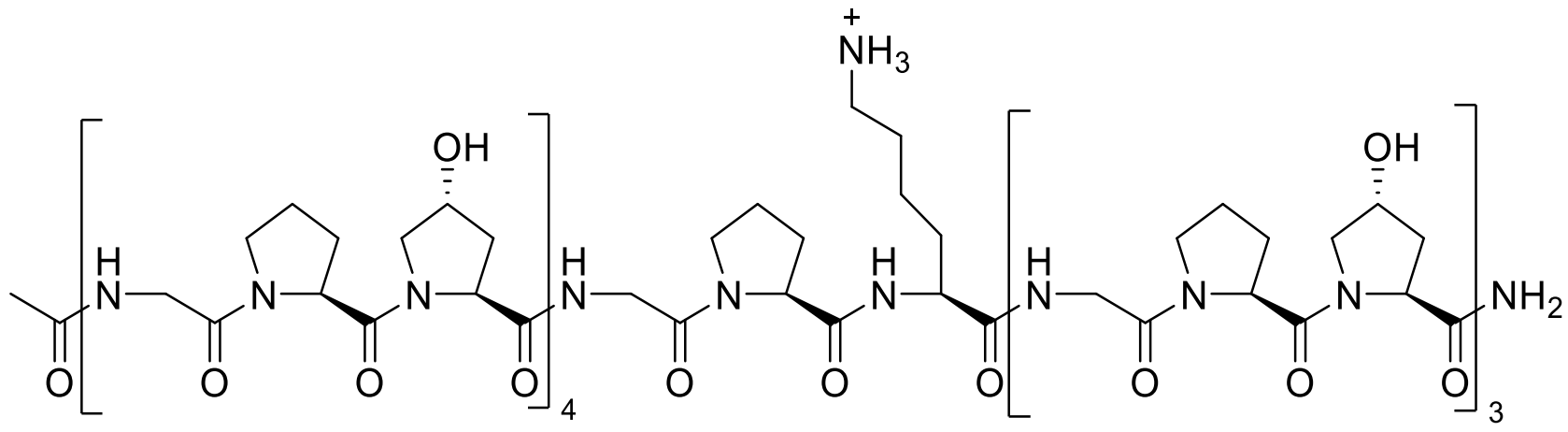

The resin was swollen according to protocol A, and the peptide was synthesized by microwave-assisted automated synthesis according to protocol C up to a length of 18 amino acids. After washing with DMF for 1 min (5×), Fmoc was deprotected according to protocol G, and Fmoc-GPO(tBu)-OH was manually coupled according to protocol F. Fmoc deprotection and manual coupling of Fmoc-GlyProHyp(tBu)-OH were performed once more. N-terminal Fmoc was deprotected according to protocol G, the peptide was acetyl capped according to protocol H, and finally cleaved from the resin according to protocol I. The crude product was purified by preparative RP HPLC using a gradient of 95–88% solvent A over 30 min.

Purity (analytical HPLC, column 1, 65 °C): >97%

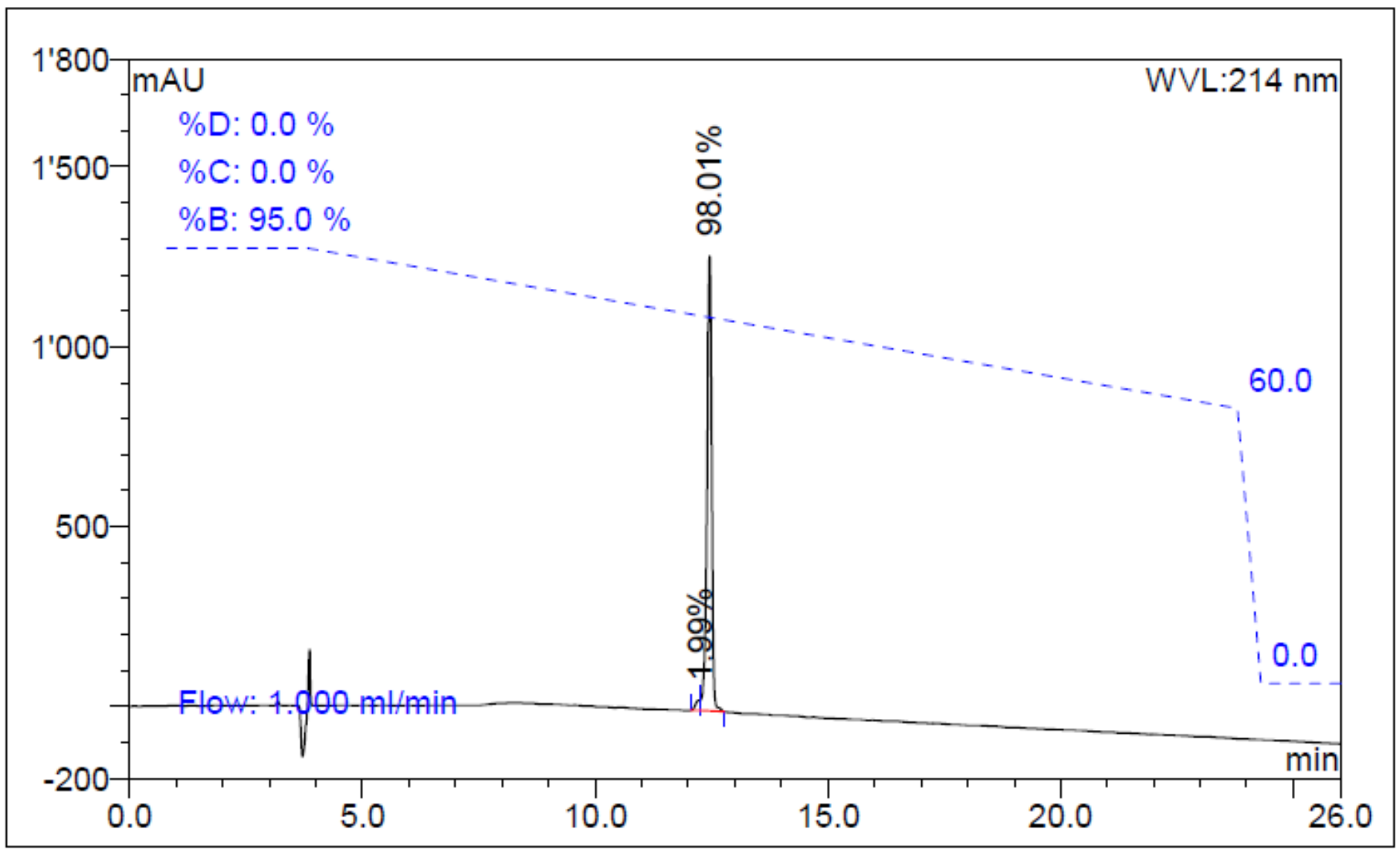

HRMS (MALDI/ESI+): calc. *m/z* for $C_{99}H_{147}N_{26}O_{32}$ $[M+H]^+$ = 2212.0669, found: 2212.0661.

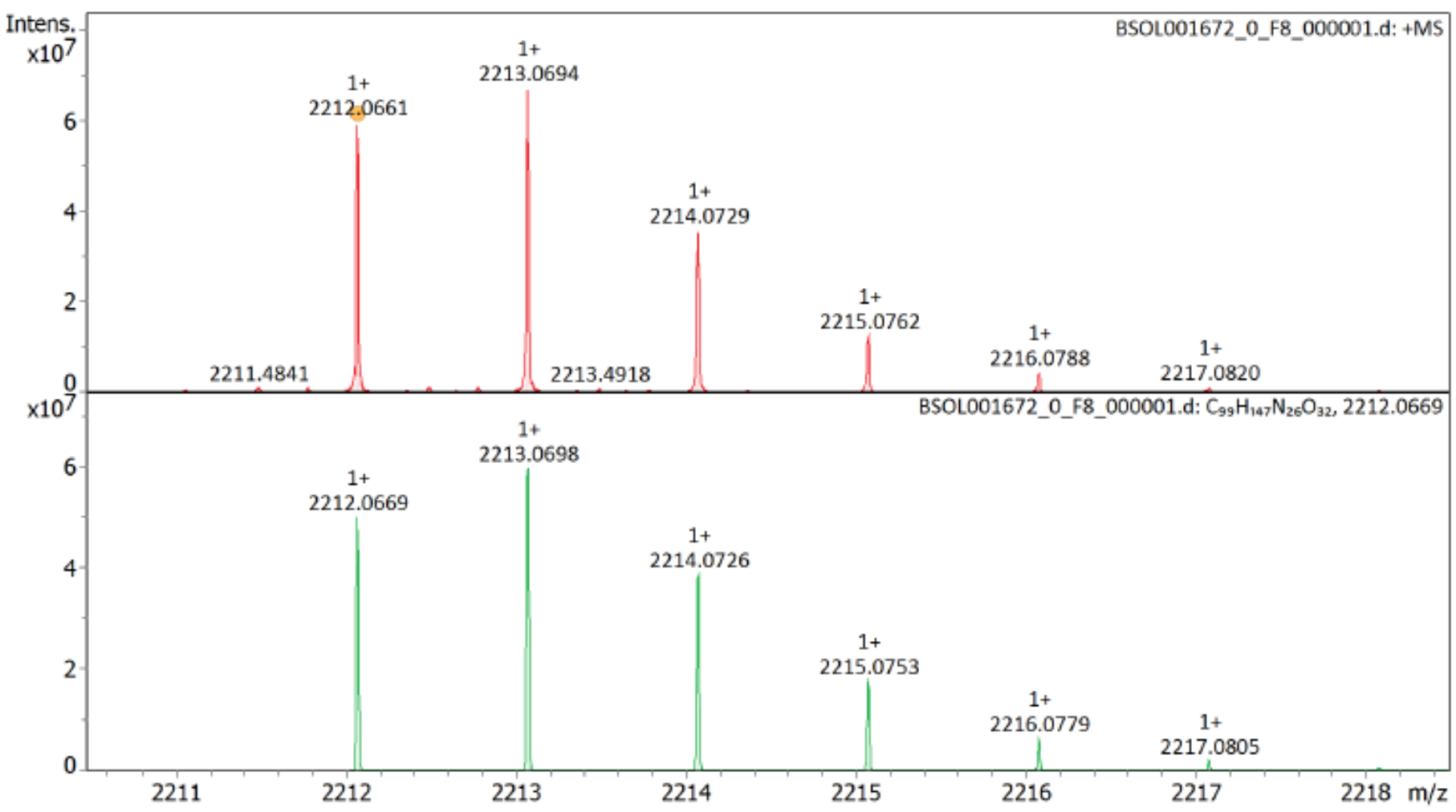

Ac-(GPO)$_2$GPKGPO^{13}<u>G</u>POGPO-NH$_2$ (**18-Y**; ^{13}C-label on Gly-13)

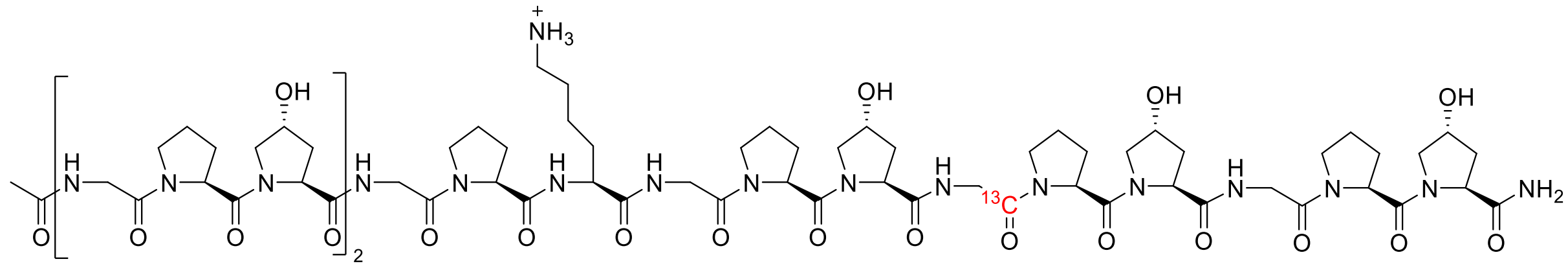

The resin was swollen according to protocol A, and the peptide was synthesized by microwave-assisted automated synthesis according to protocol C up to a length of four amino acids. After washing with DMF for 1 min (5×), Fmoc was deprotected according to protocol G, and Fmoc-^{13}C-Gly-OH was manually coupled according to protocol E. The remaining sequence was coupled by microwave-assisted automated synthesis according to protocol C, N-terminal Fmoc was deprotected according to protocol G, the peptide was acetyl capped according to protocol H, and finally cleaved from the resin according to protocol I. The crude product was purified by preparative RP HPLC using a gradient of 95–90% solvent A over 30 min.

Purity (analytical HPLC, column 2, 65 °C): >97%

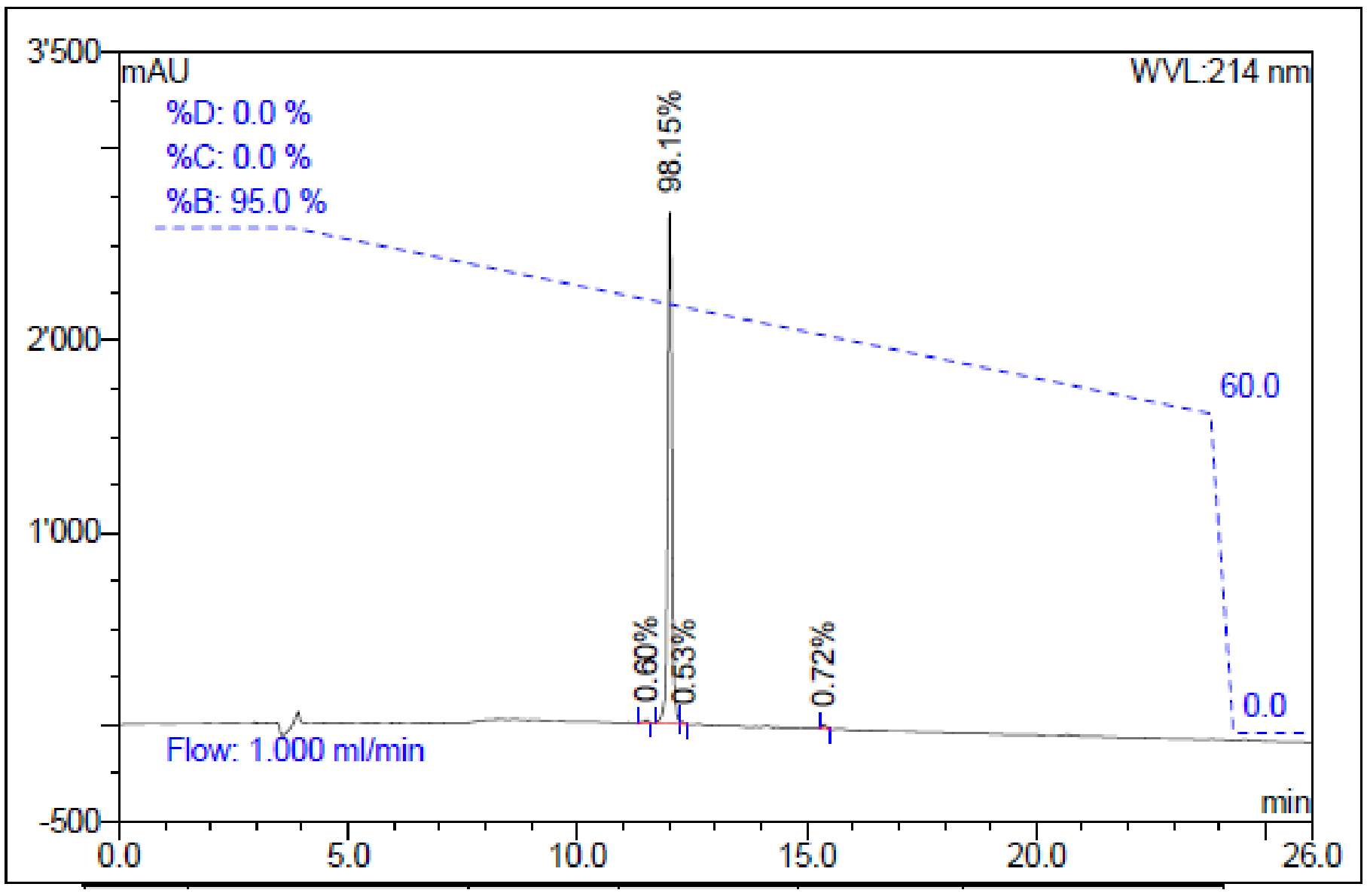

HRMS (ESI+): calc. *m/z* for $C_{74}H_{114}N_{20}O_{24}{}^{13}C$ $[M+2H]^{2+}$ = 839.9169, found: 839.9188.

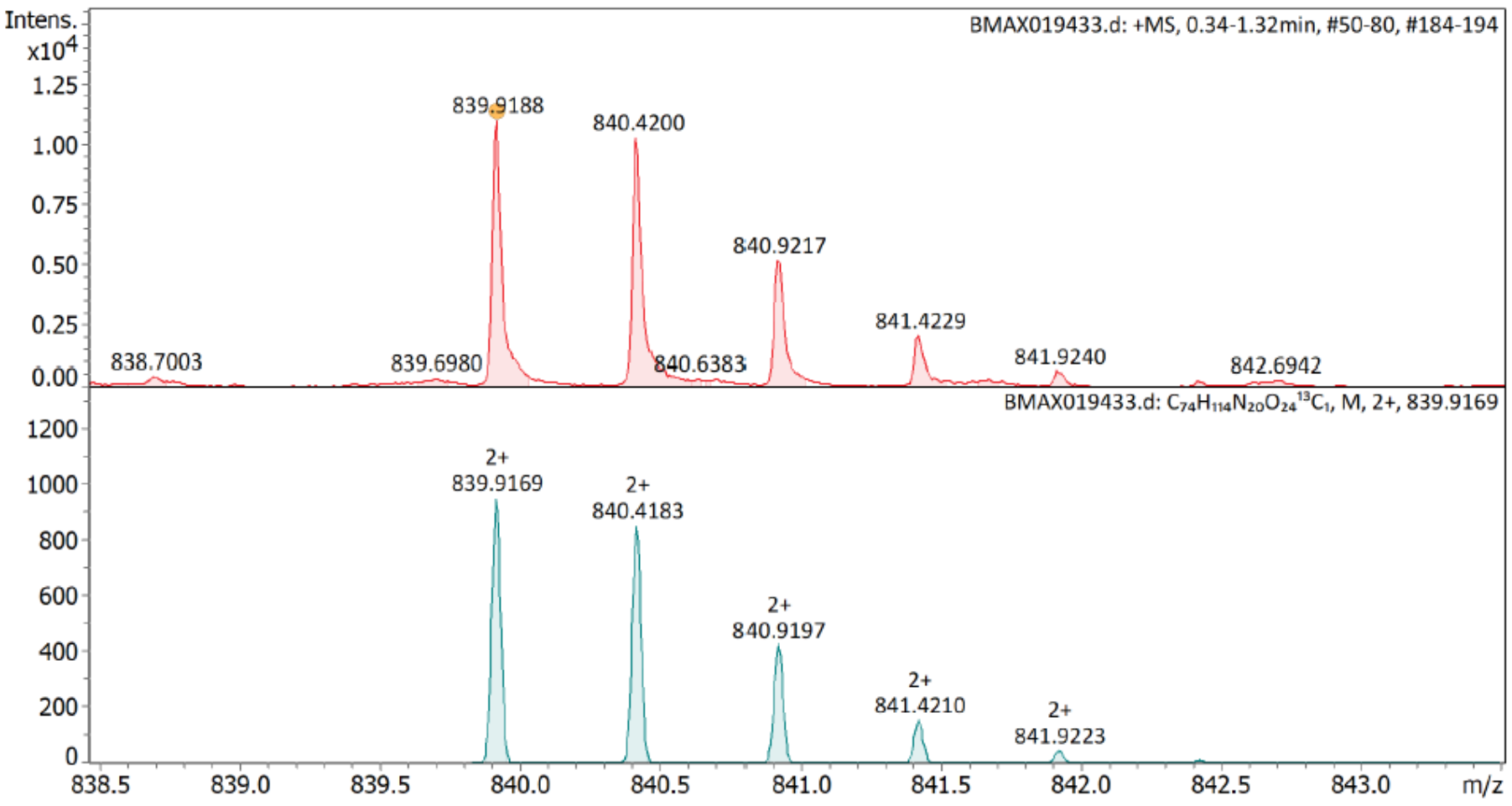

Ac-(GPO)$_4$GPKGPO^{13}<u>G</u>POGPO-NH$_2$ (**24-Y**; ^{13}C-label on Gly-19)

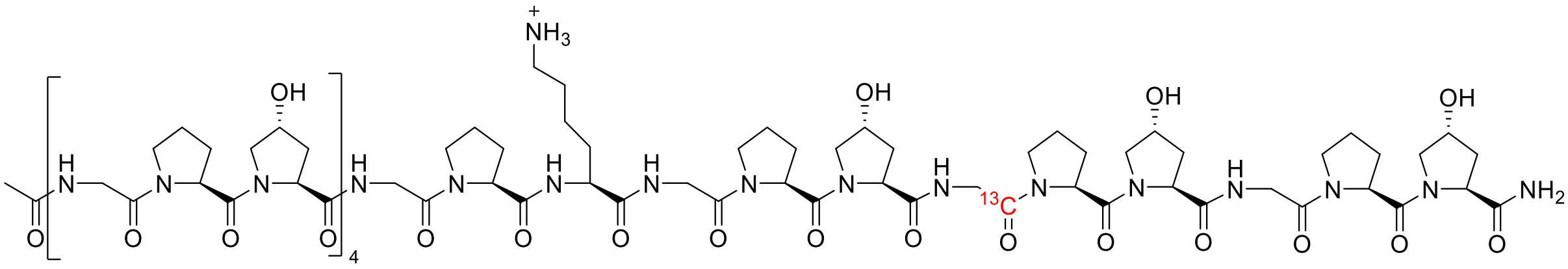

The resin was swollen according to protocol A, and the peptide was synthesized by microwave-assisted automated synthesis according to protocol C up to a length of four amino acids. After washing with DMF for 1 min (5×), Fmoc was deprotected according to protocol G, and Fmoc-^{13}C-Gly-OH was manually coupled according to protocol E. The remaining sequence was coupled by microwave-assisted automated synthesis according to protocol C up to a length of 18 amino acids. After washing with DMF for 1 min (5×), Fmoc was deprotected according to protocol G, and Fmoc-GPO(tBu)-OH was manually coupled according to protocol F. Fmoc deprotection and manual coupling of Fmoc-GPO(tBu)-OH were performed once more. N-terminal Fmoc was deprotected according to protocol G, the peptide was acetyl capped according to protocol H, and finally cleaved from the resin according to protocol I. The crude product was purified by preparative RP HPLC using a gradient of 95–87% solvent A over 30 min.

Purity (analytical HPLC, column 2, 65 °C): >97%

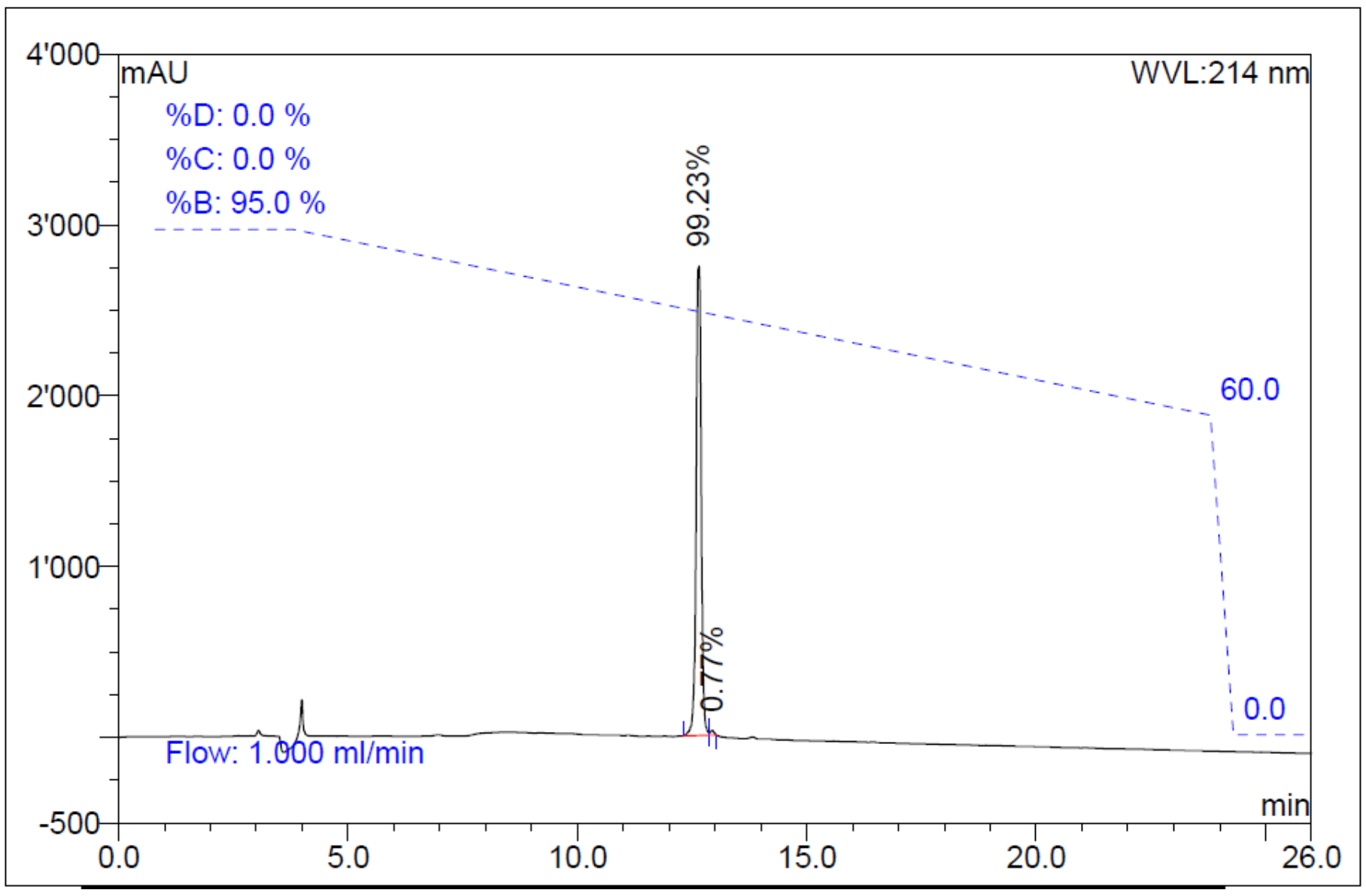

HRMS (ESI+): calc. *m/z* for $C_{98}H_{146}N_{26}NaO_{32}{}^{13}C$ $[M+2H]^{2+}$ = 1129.0207, found: 1129.0214.

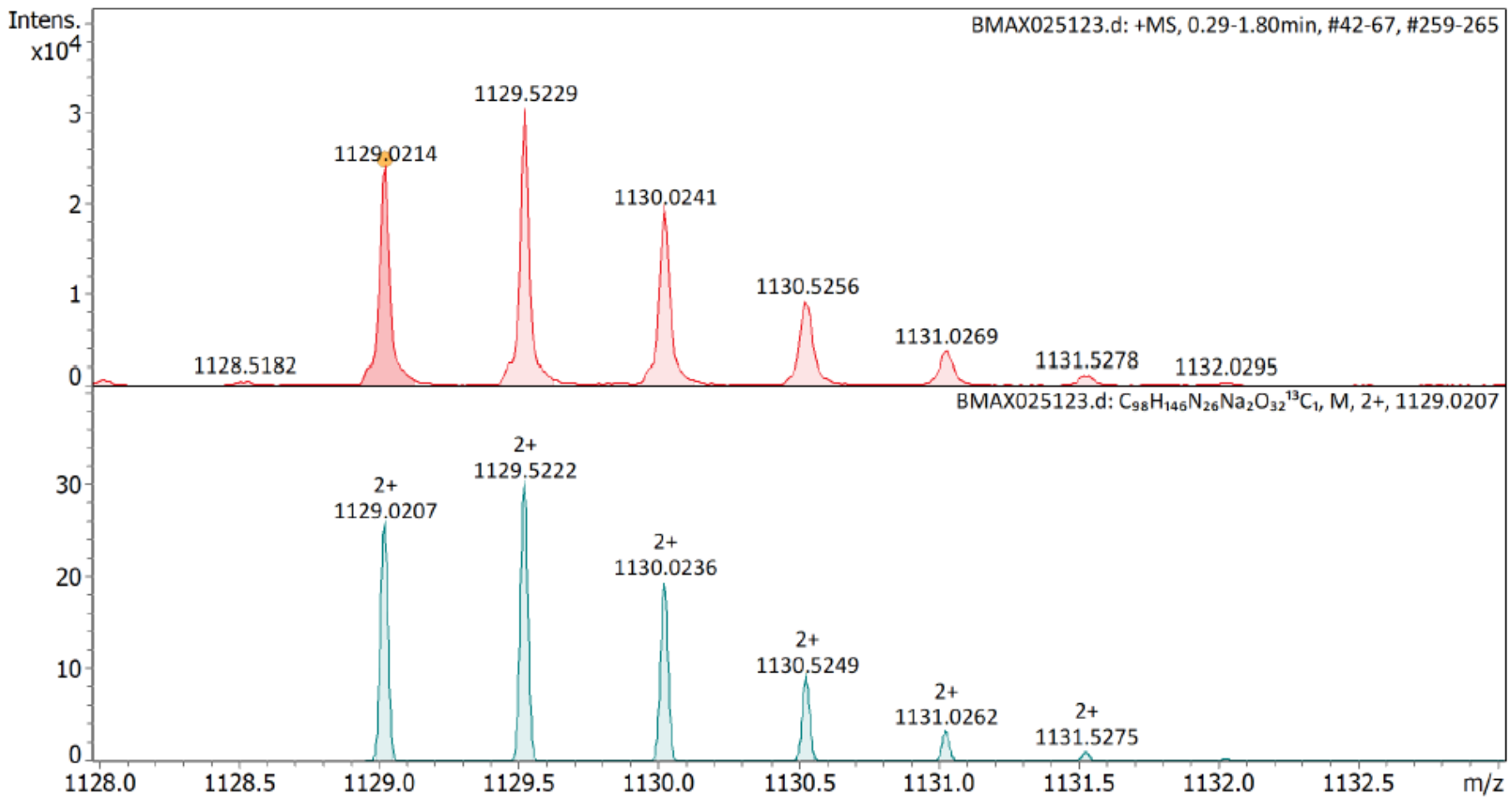

Ac-(POG)$_6$-NH$_2$ ((POG)$_6$)

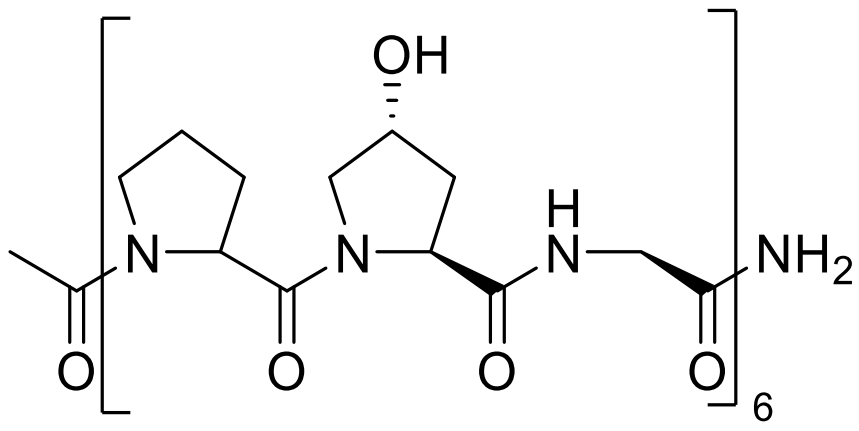

The resin was swollen according to protocol A, the peptide was synthesized by microwave-assisted automated synthesis according to protocol C, N-terminal Fmoc was deprotected according to protocol G, the peptide was acetyl capped according to protocol H, and finally cleaved from the resin according to protocol I. The crude product was purified by preparative RP HPLC using a gradient of 95–88% solvent A over 30 min.

Purity (analytical HPLC, column 1, 65 °C): >97%

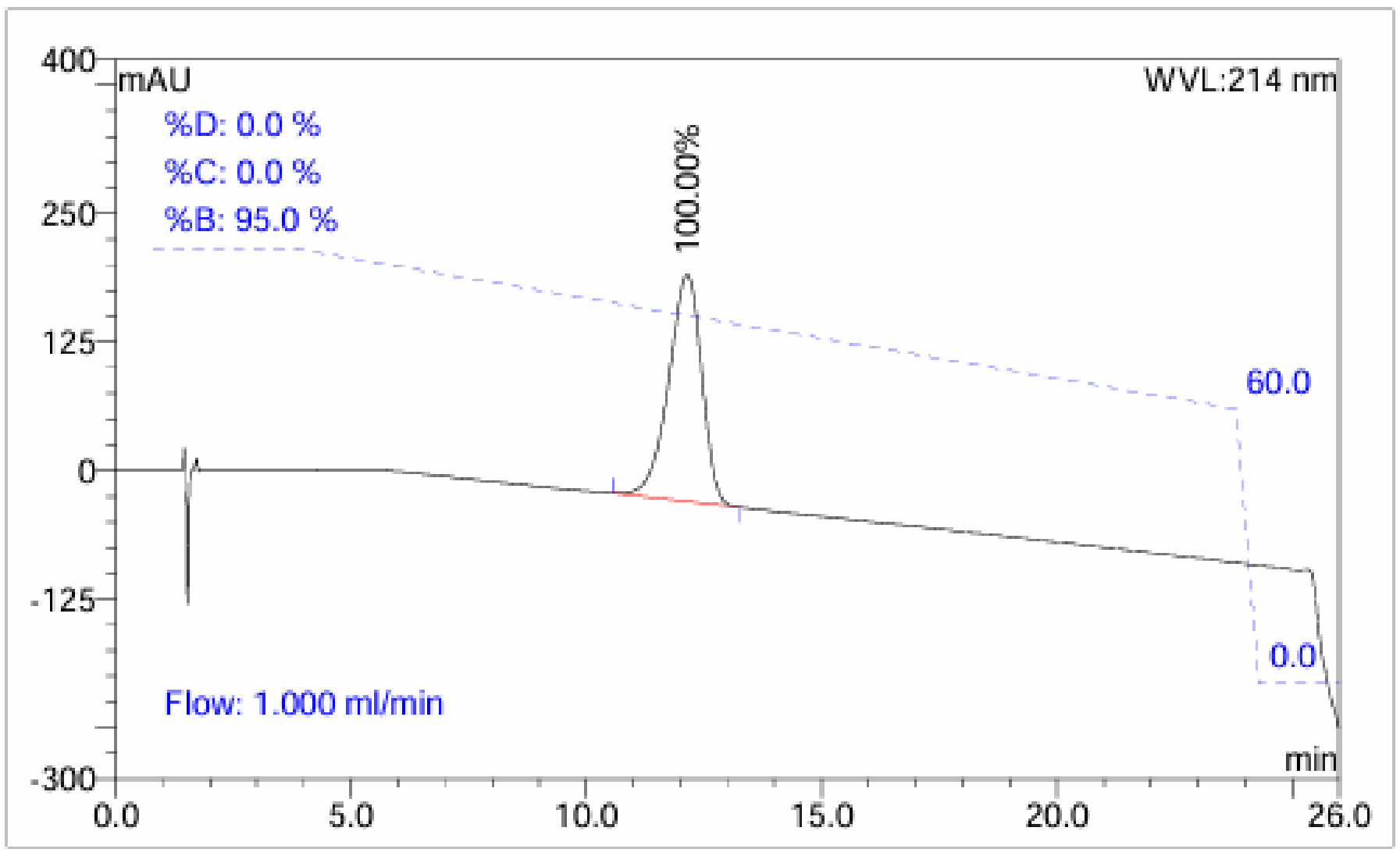

HRMS (MALDI/ESI): calc. *m/z* for $C_{74}H_{108}N_{19}O_{25}$ $[M+H]^+$ = 1662.7758, found: 1662.7776.

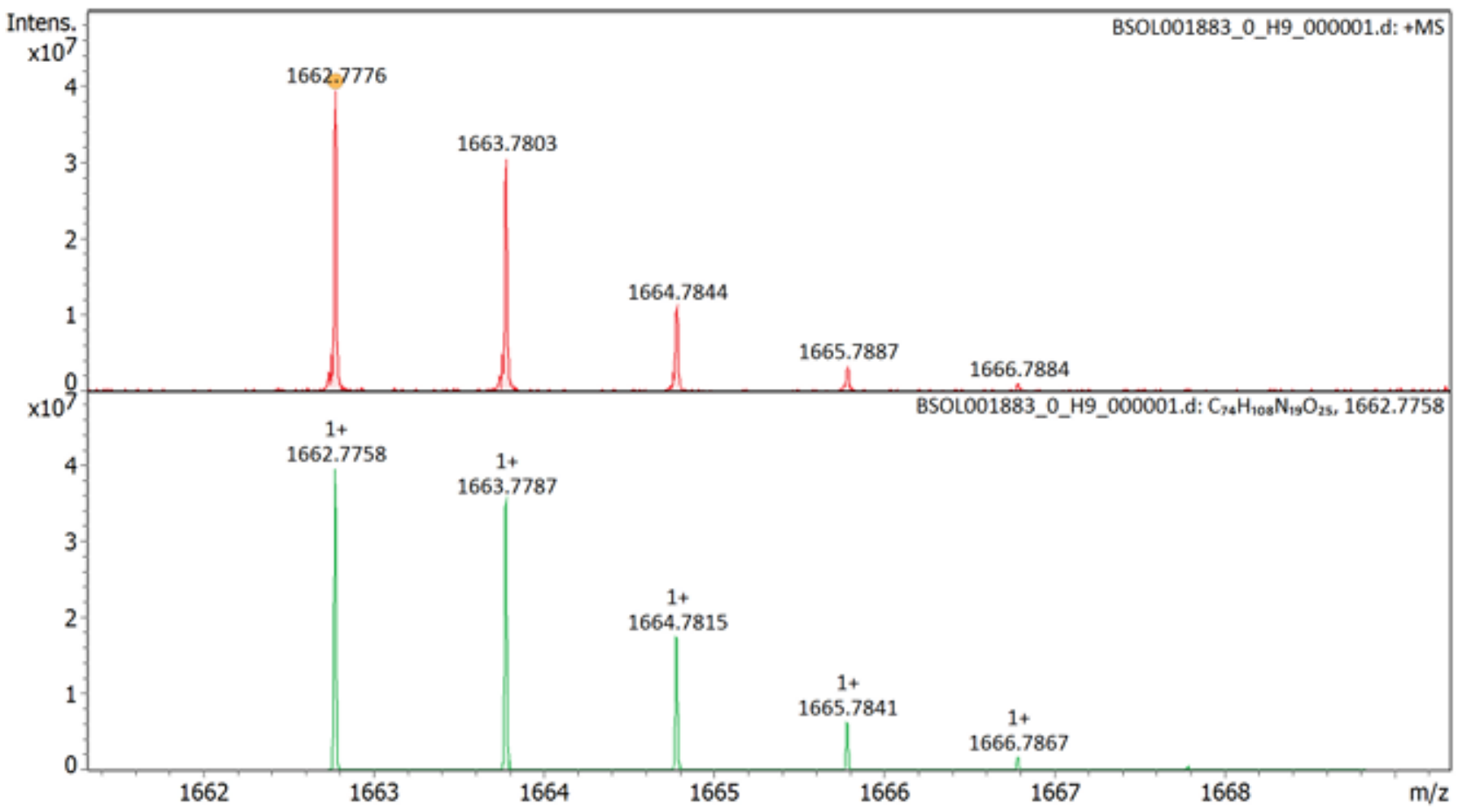

3. CD spectroscopy

3.1 General procedure of CD measurements

The lyophilized CMPs were dissolved either in PBS, pH 7.4 (Gibco, 10010-015), or 50 mM sodium borate containing 0.1% BSA, pH 8.5 (assay buffer) to a concentration of 200 µM. The solutions were subsequently annealed by heating to 80 °C for 15 min in a water bath, cooling to room temperature or 29 °C, and storage at 4 °C or 29 °C overnight prior to CD spectroscopic analysis and thermal denaturation. Notably, the instrument was not calibrated, and the absolute θ_{225} values cannot be compared between different CMPs.

3.2 CD spectra in PBS, pH 7.4

CD spectra were recorded using a spectral bandwidth of 1 nm at 7 °C with a time constant of 5 s and a step resolution of 1 nm.

Ac-$(POG)_6$-NH_2
$(POG)_6$

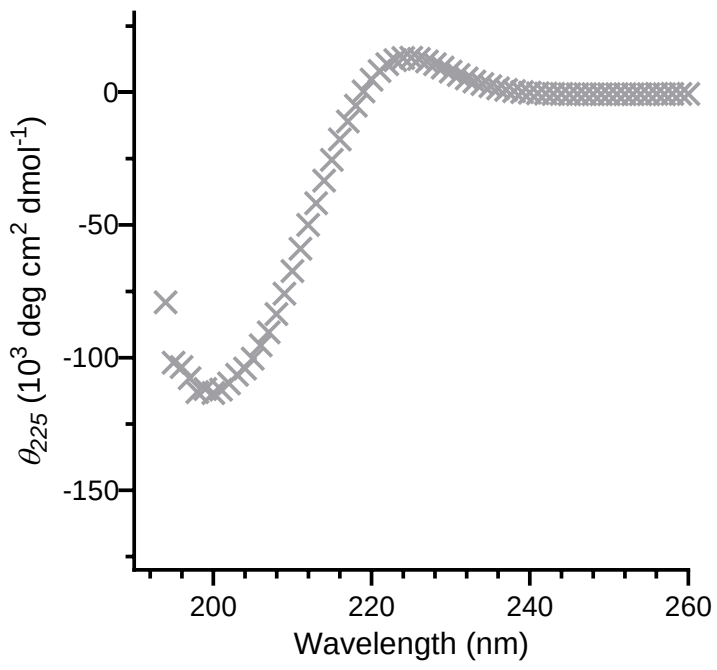

Ac-$(GPO)_2$GKP$(GPO)_3$-NH_2
(18-X)

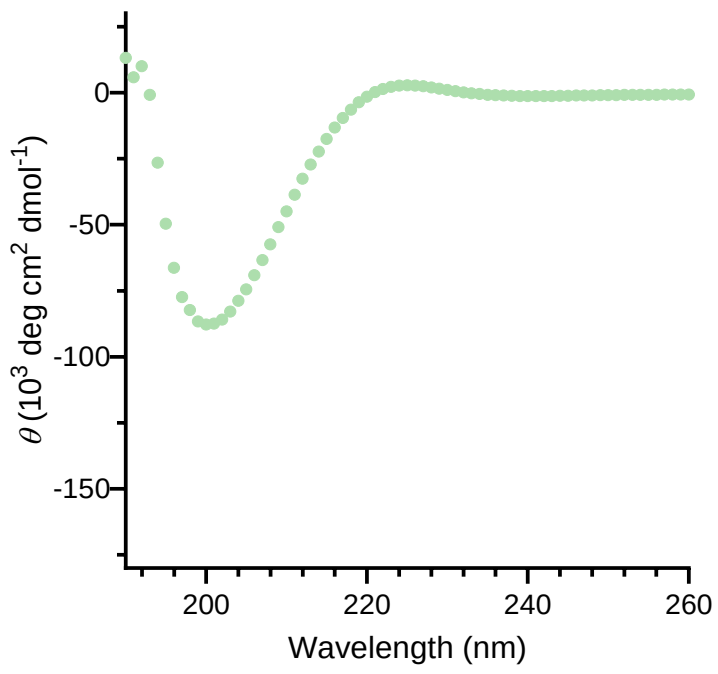

Ac-$(GPO)_3$GKP$(GPO)_3$-NH_2
(21-X)

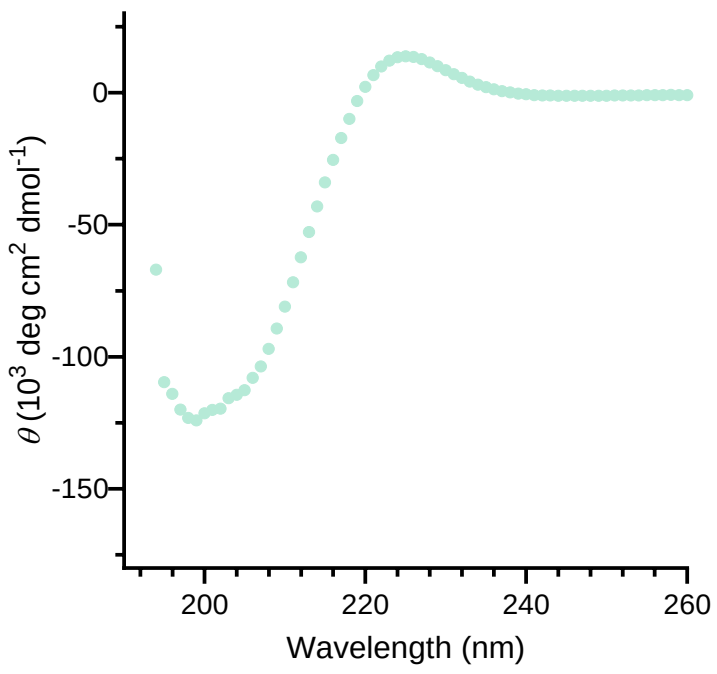

Ac-$(GPO)_4$GKP$(GPO)_3$-NH_2
(24-X)

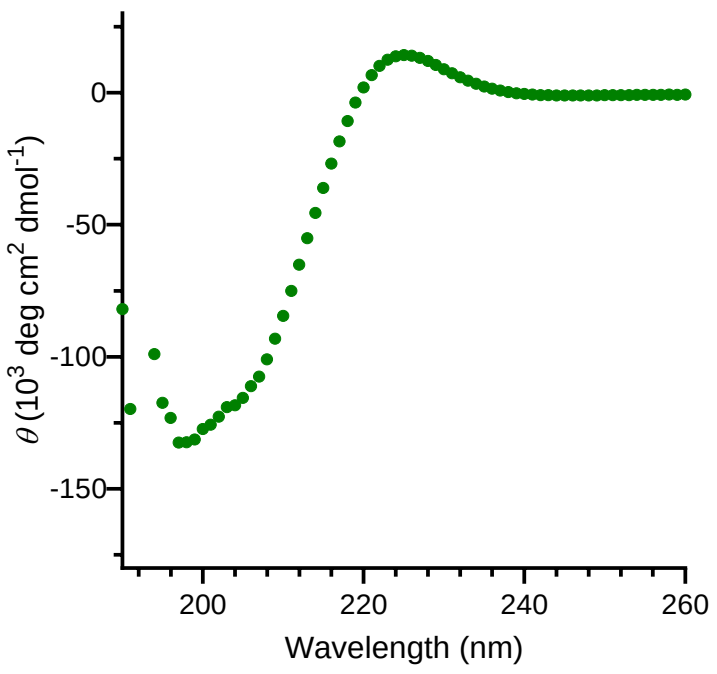

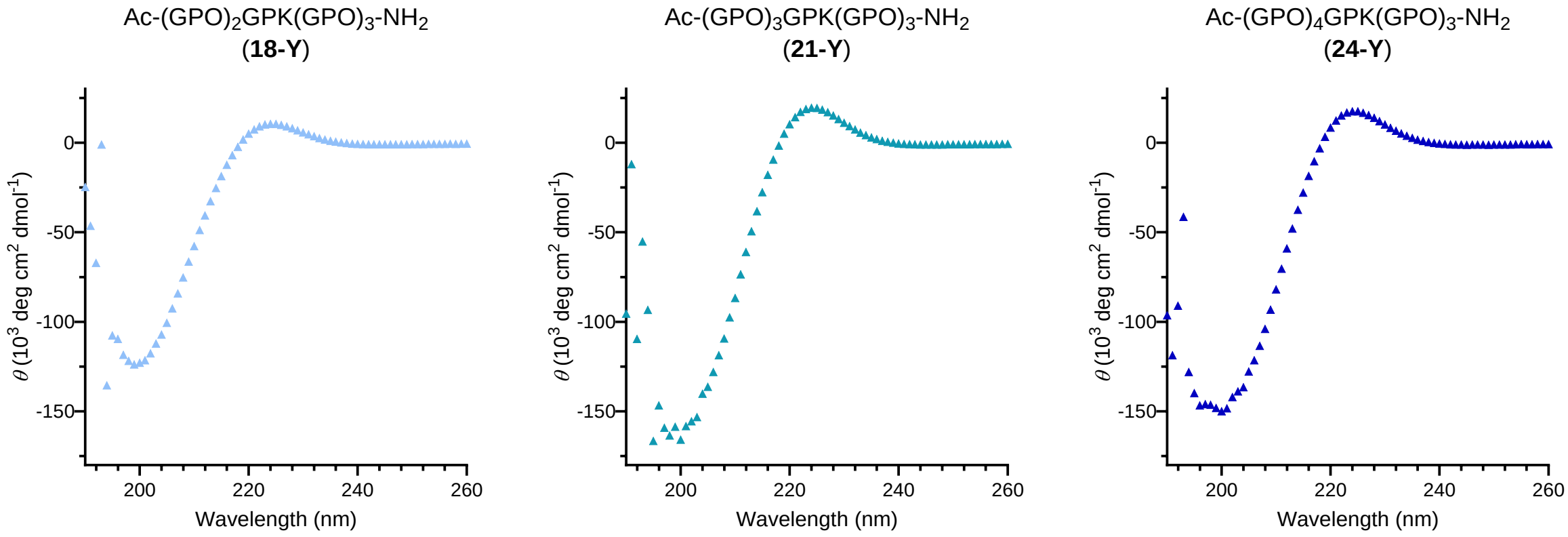

Figure S8 Representative CD spectra of the CMPs (200 µM) in PBS, pH 7.4; n = 2 independent experiments.

3.3 CD spectra in 50 mM sodium borate containing 0.1% BSA, pH 8.5

CD spectra were recorded using a spectral bandwidth of 1 nm at 29 °C with a time constant of 5 s and a step resolution of 1 nm; n = 2. Notably, BSA contributes to the observed CD spectra.

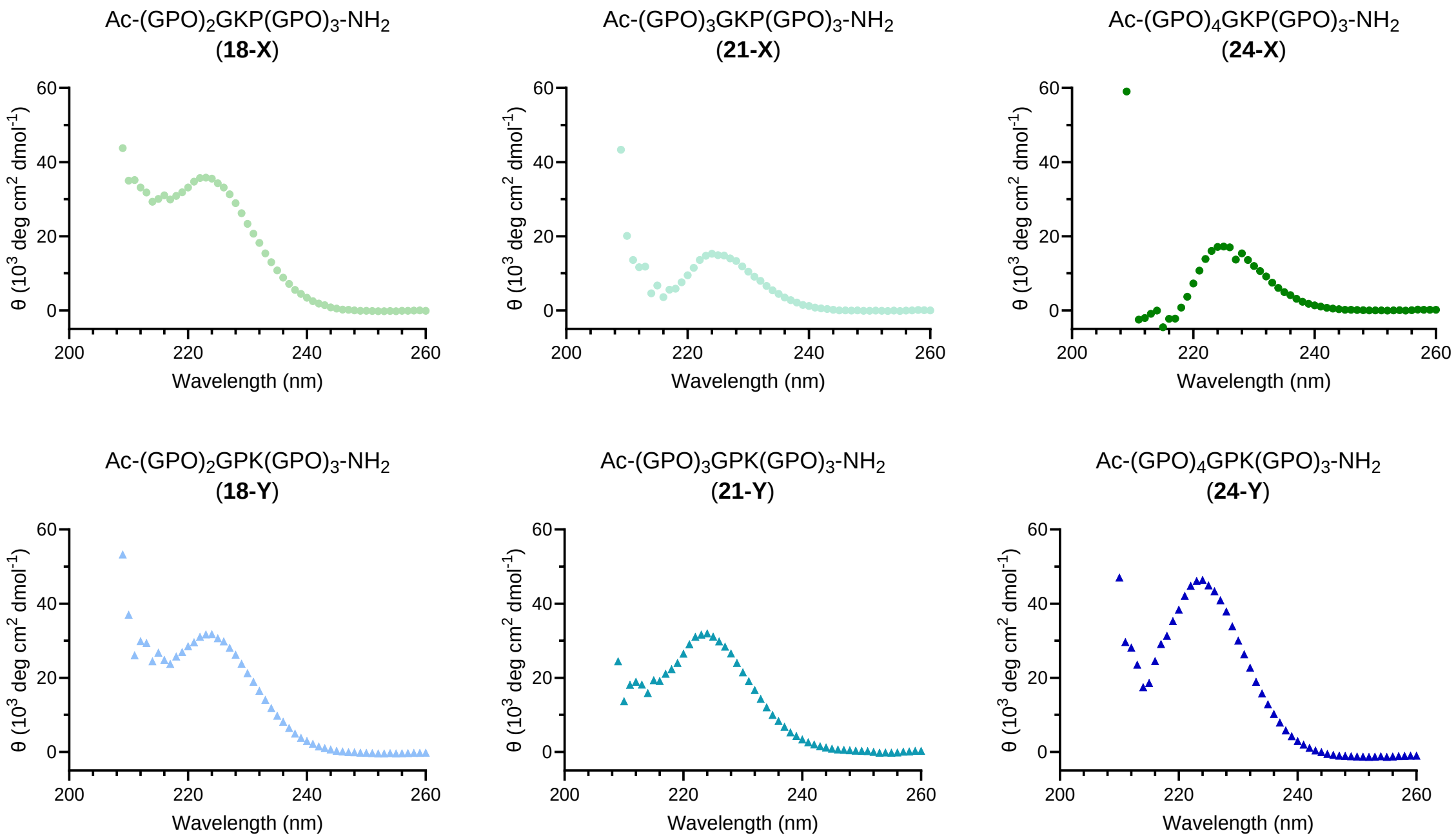

Figure S9 Representative CD spectra of the CMPs (200 µM) in assay buffer, pH 8.5, after background subtraction of the assay buffer; n = 2 independent experiments.

3.4 General procedure of thermal denaturation studies

The unfolding of the triple helices was monitored at 225 nm with a spectral bandwidth of 1 nm and a time constant of 5 s (2 × 5 s measurement + 2 × 2 s shutter delay = 14 s per data point). The temperature was increased in steps of 1 °C from 7 °C to 80 °C and from 29 °C to 90 °C for the experiments in PBS, pH 7.4, and 50 mM sodium borate containing 0.1% BSA, pH 8.5, respectively. Each data point was recorded after an equilibration time of 100 s (overall heating rate = 1 °C/114 s); n = 2 independent experiments. The recorded data were fitted to an all-or-none transition in which three single strands combine to a triple helix, as previously reported by Engel et al.[4,5] The fitting was performed using GraphPad Prism 9 (GraphPad Software, San Diego, CA, USA).

3.5 Thermal denaturation studies in PBS, pH 7.4

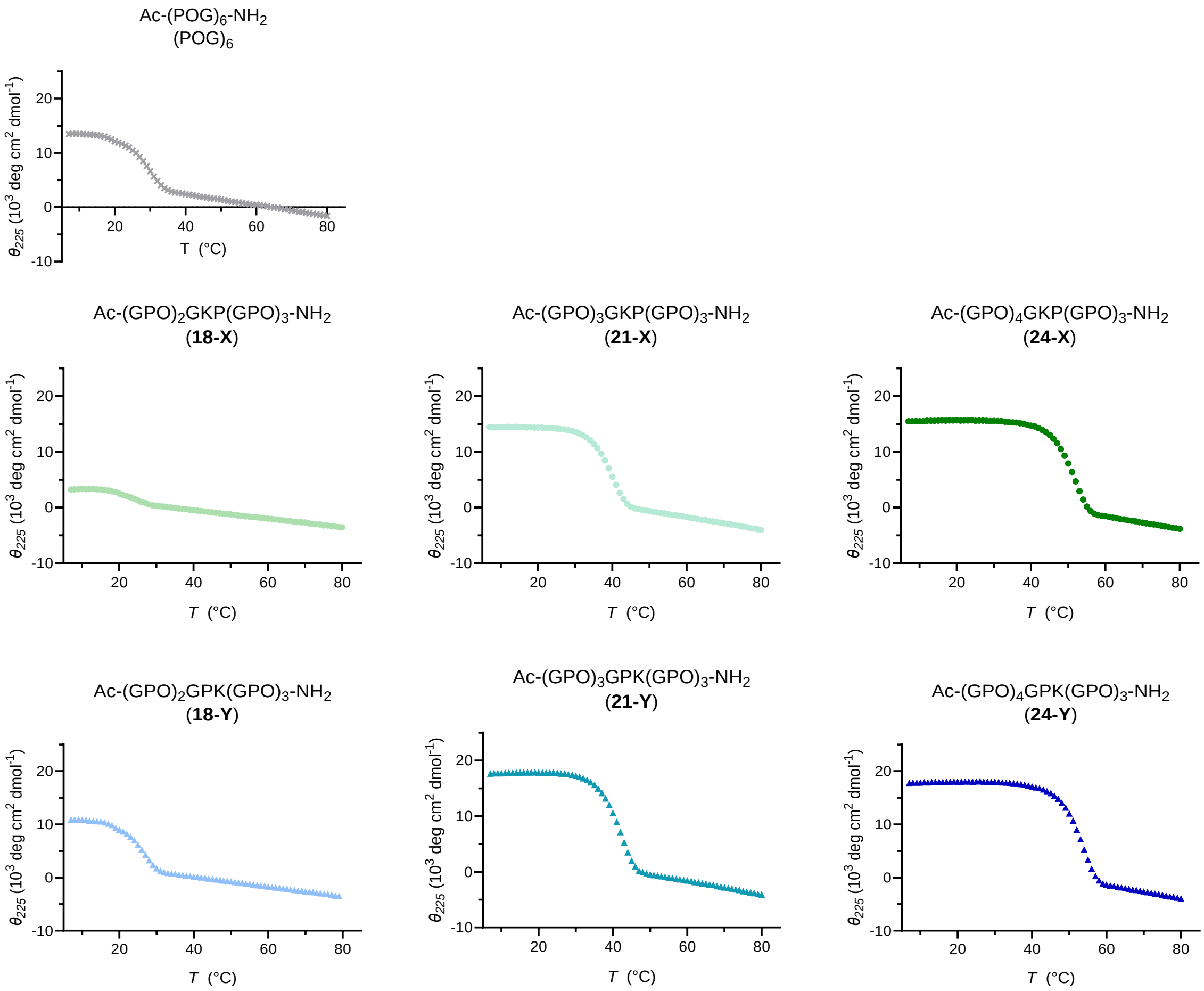

Figure S10 Thermal denaturation curves of triple helices derived from CMPs (200 µM) with Lys either at the Xaa or Yaa position in PBS, pH 7.4; n = 2 independent experiments.

3.6 Thermal denaturation studies in 50 mM sodium borate containing 0.1% BSA, pH 8.5

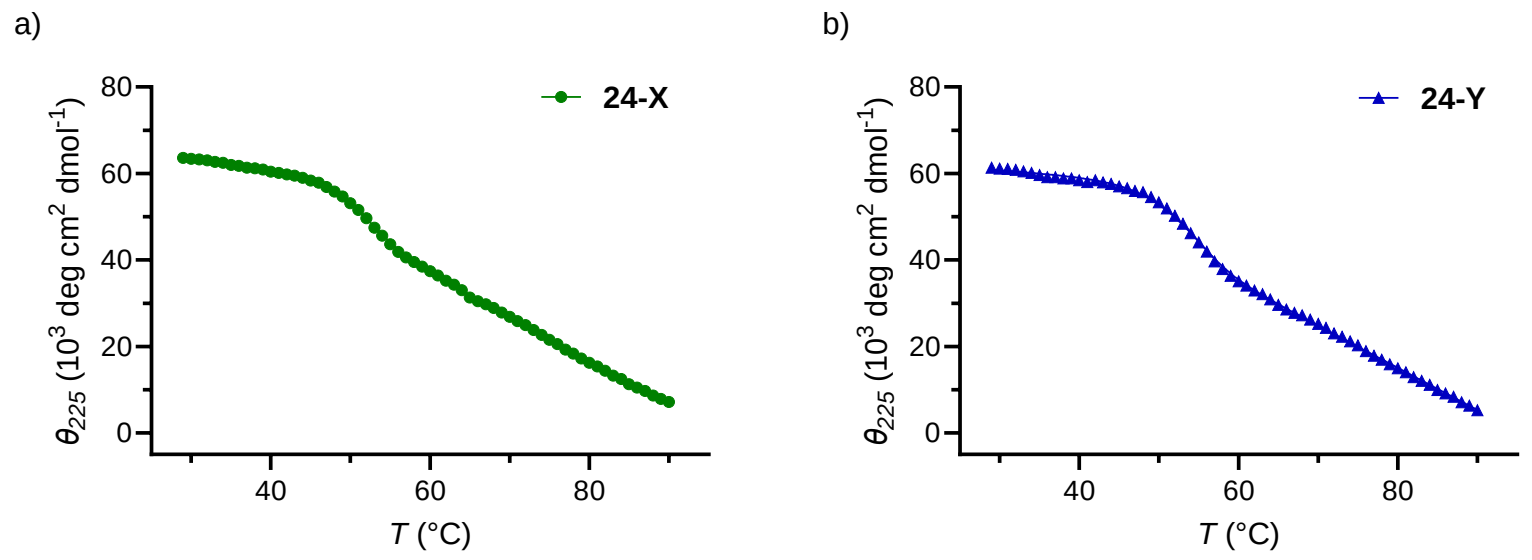

Figure S11 Thermal denaturation curves of **24-X** and **24-Y** triple helices (200 µM) with Lys either at the Xaa or Yaa position in assay buffer, pH 8.5; n = 2 independent experiments.

4. NMR spectroscopy

4.1 General procedure of NMR measurements

Quantitative ^{13}C nuclear magnetic resonance (NMR) spectra of ^{13}C-labeled CMPs were measured by the NMR service of the Laboratory of Organic Chemistry at the ETHZ on a Bruker AV III 600 (600/150MHz). Solutions (15 mM) of the ^{13}C-labeled CMPs in 50 mM sodium borate containing 0.1% BSA (pH 8.5) were annealed, as described for CD measurements and LOXL2 activity studies (*vide supra*), and stored at 29 °C overnight. Then, the solutions were diluted with assay buffer and D_2O, and spectra of the solutions (4 mM), containing 12% D_2O, were recorded at 29 °C. NMR data were analyzed using MestReNova.

4.2 Fmoc-^{13}C-1-Gly

^1H NMR (400 MHz, MeOD)

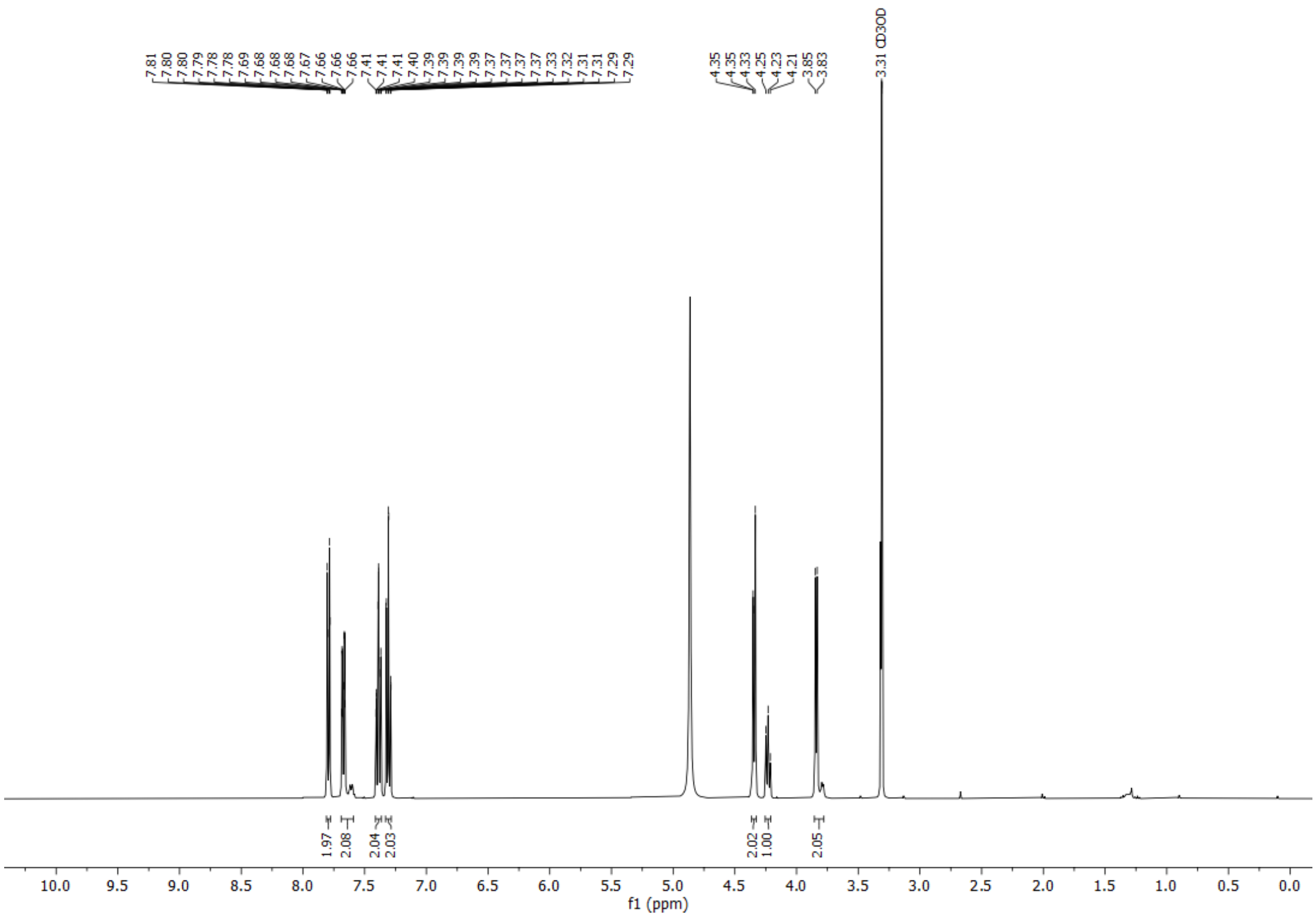

^{13}C NMR (101 MHz, MeOD)

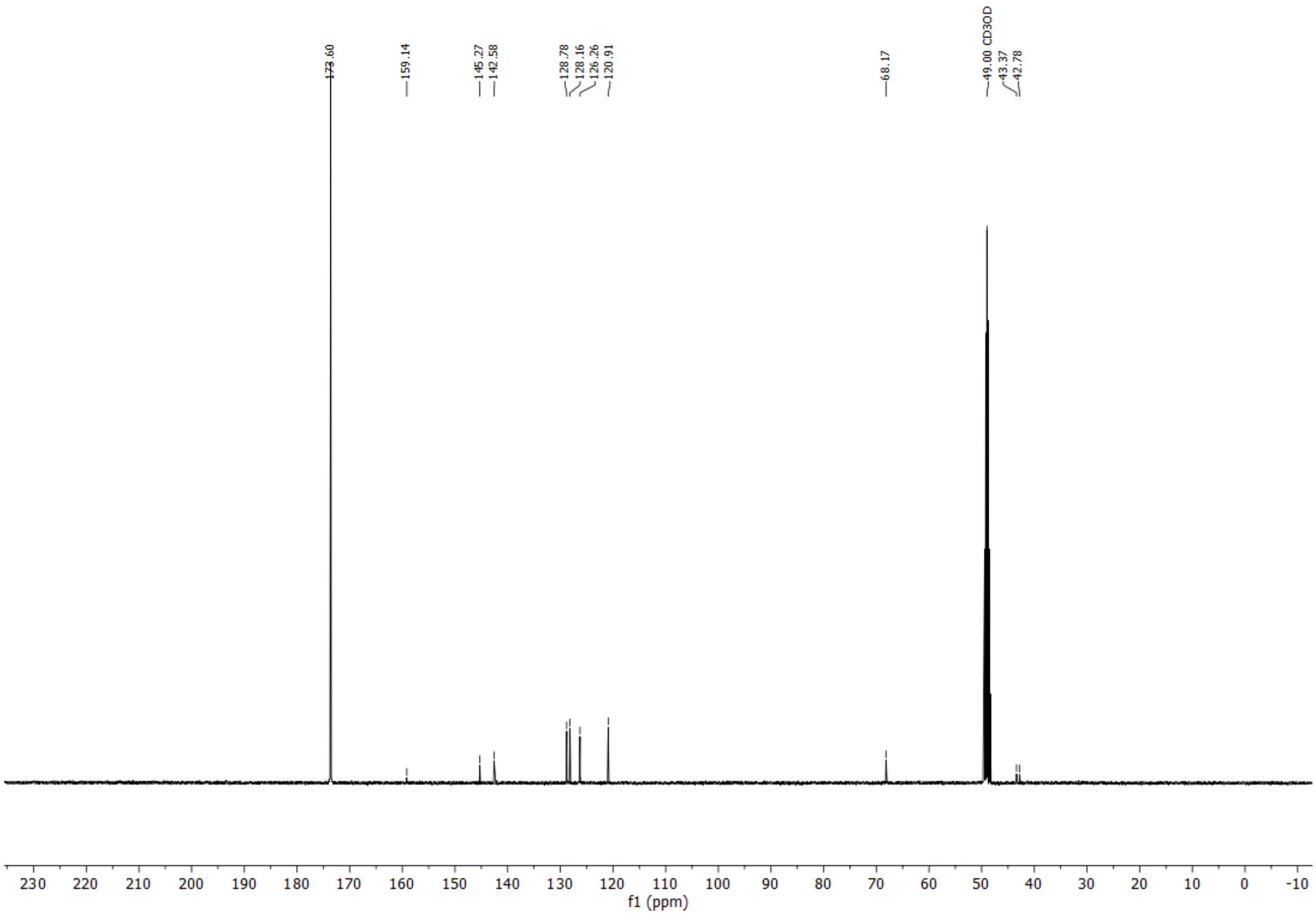

4.3 Ac-(GPO)$_2$GPKGPO^{13}GPOGPO-NH$_2$ (**18-Y**; ^{13}C-label on Gly-13)

18-Y (4 mM)

^{13}C NMR (151 MHz, H_2O+D_2O) δ 168.49, 167.00 (d, *J* = 37.4 Hz).

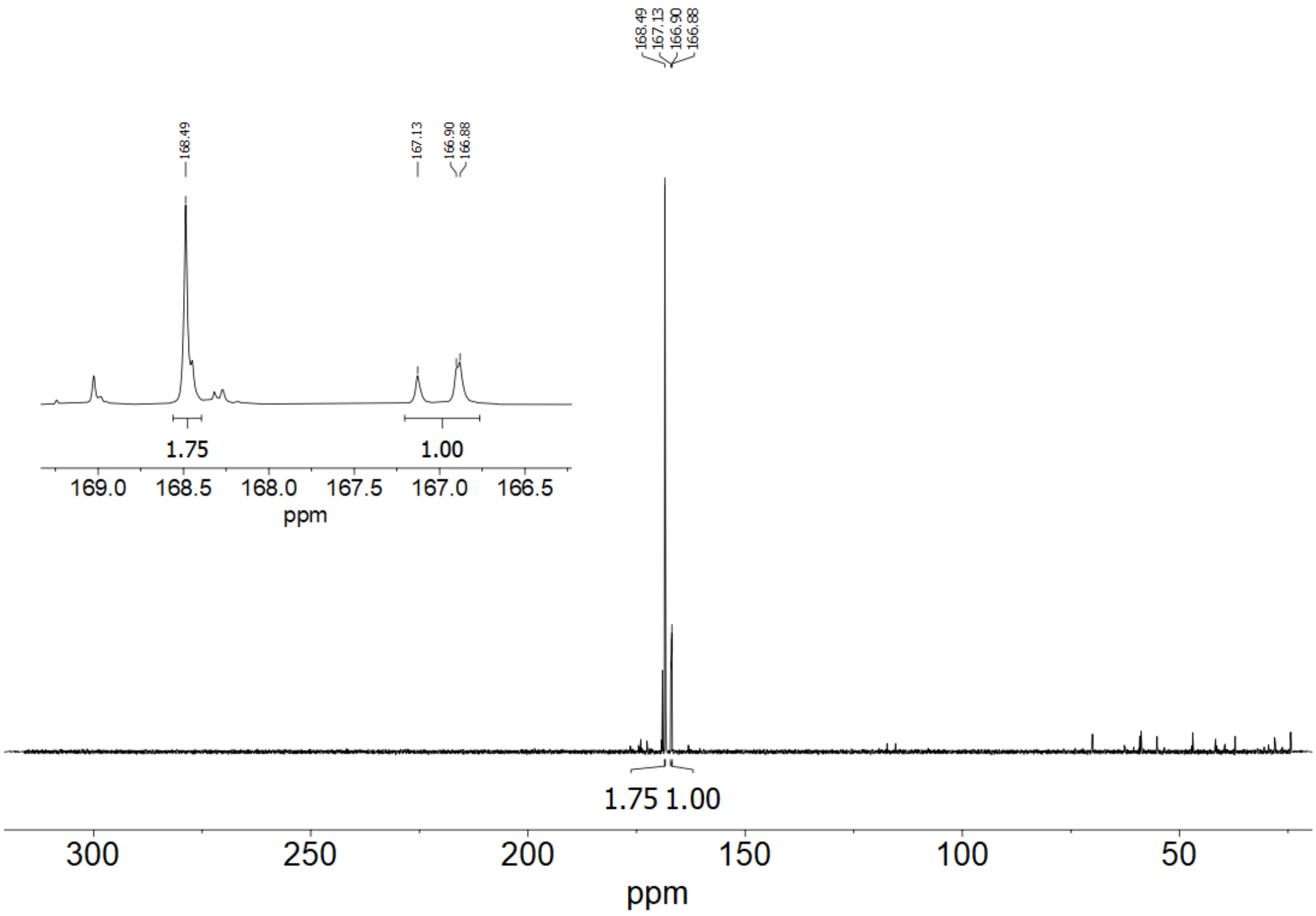

The triple helix/single strand equilibrium was assessed over time. After storage at 4 °C for one week, the same sample was re-annealed and analysed as described before (*vide supra*). Reassuringly, a comparable triple helix/single strand ratio was obtained.

^{13}C NMR (151 MHz, H_2O+D_2O) δ 168.49, 167.00 (d, *J* = 37.5 Hz).

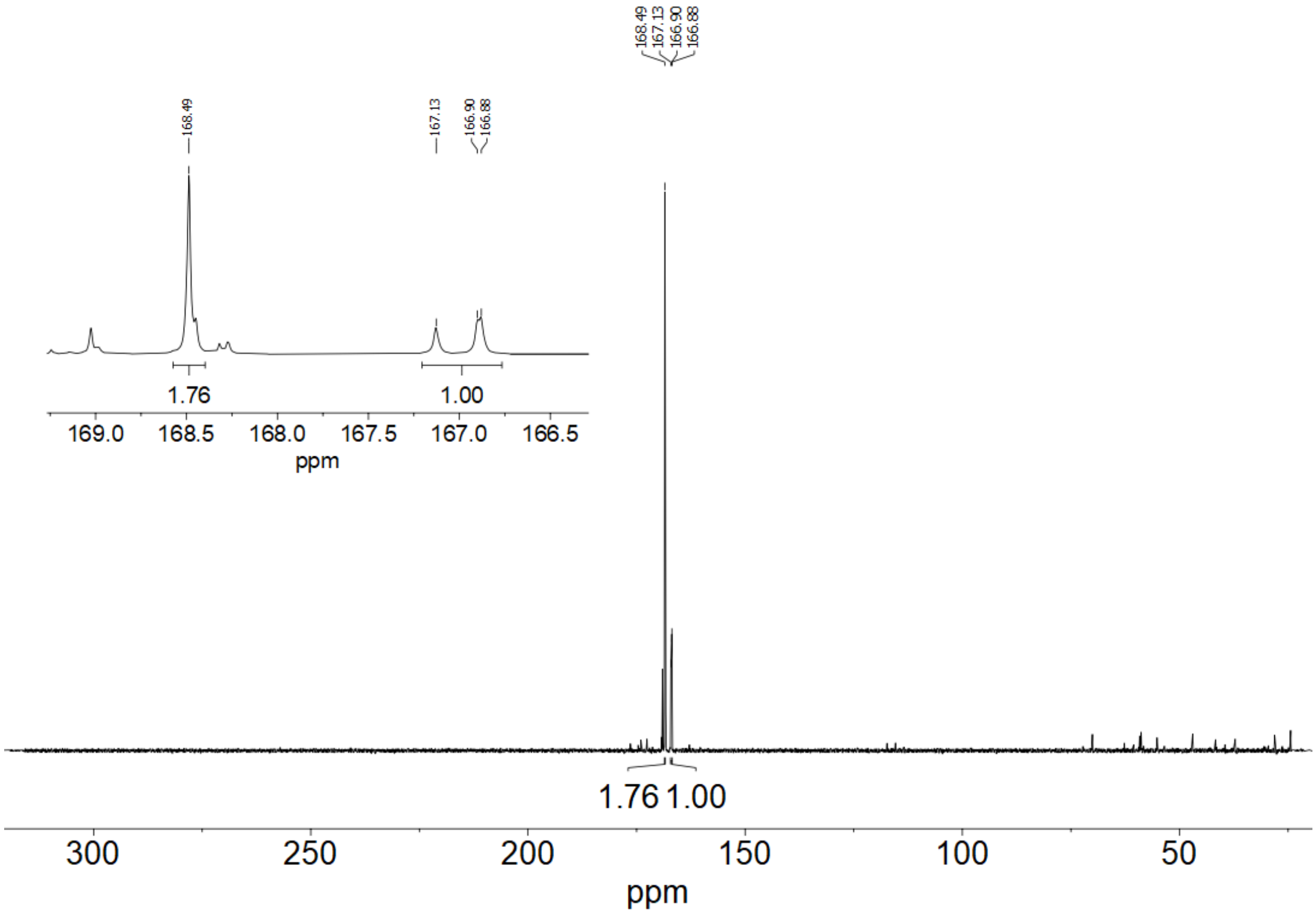

18-Y (0.43 mM)

^{13}C NMR (151 MHz, D_2O) δ 168.49, 167.02 (d, *J* = 34.8 Hz).

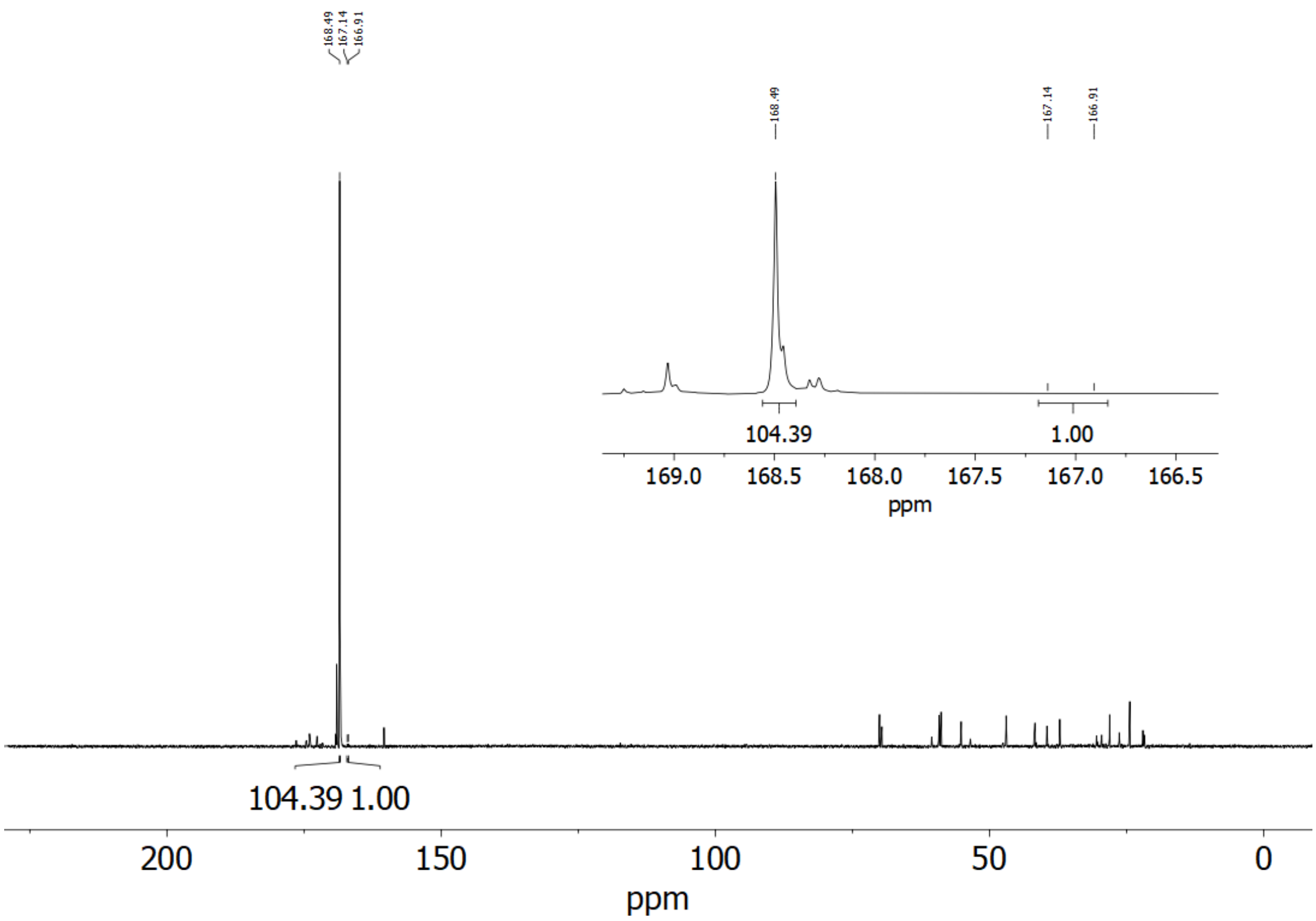

4.4 Ac-(GPO)$_4$GPKGPO^{13}<u>G</u>POGPO-NH$_2$ (**24-Y**; ^{13}C-label on Gly-19)

^{13}C NMR (151 MHz, H_2O+D_2O) δ 168.49, 167.01 (d, *J* = 37.4 Hz).

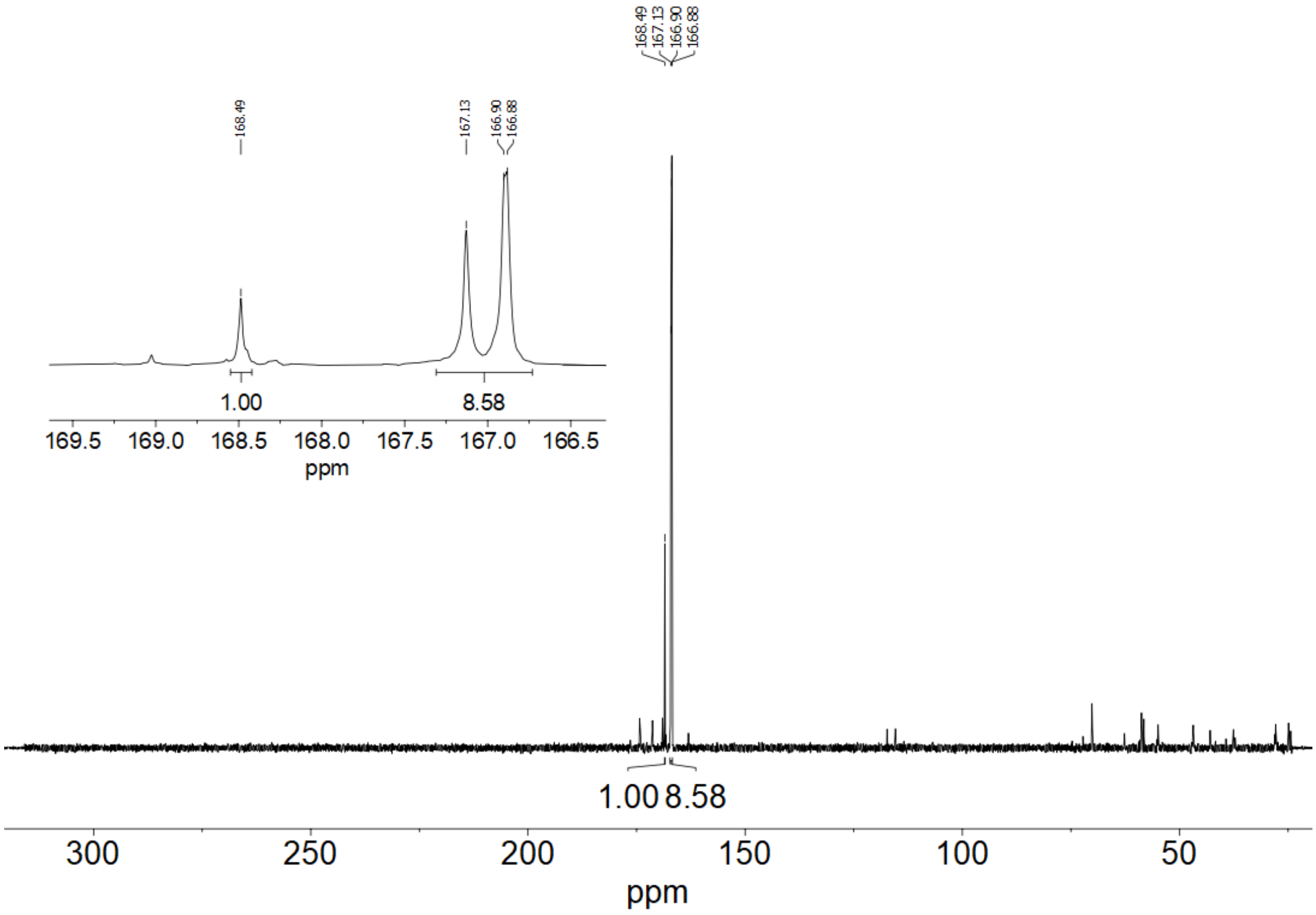

5. HRMS spectrum of the aldehyde product of **18-Y**

HRMS (MALDI/ESI+): calc. *m/z* for $C_{75}H_{110}N_{20}NaO_{25}$ $[M+Na]^+$ = 1698.7734, found: 1698.7740.

HRMS (MALDI/ESI+): calc. *m/z* for $C_{75}H_{113}N_{20}NaO_{24}$ $[M+Na]^+$ = 1699.8051, found: 1699.8057.

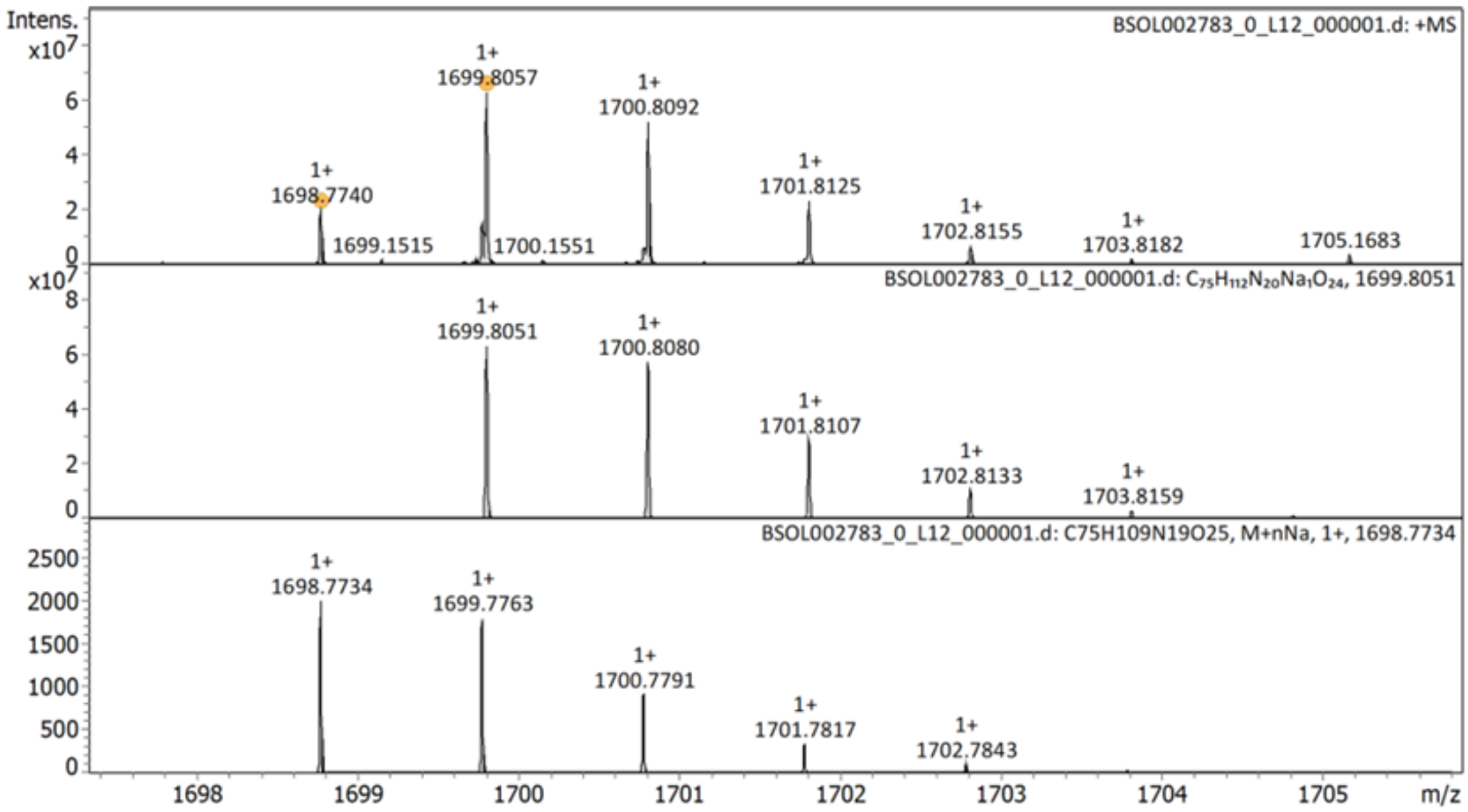

6. References

[21] P. C. Trackman, C. G. Zoski, H. M. Kagan, "Development of a peroxidase-coupled fluorometric assay for lysyl oxidase" *Anal. Biochem.* **1981**, *113*, 336–342.

[39] A. H. Palamakumbura, P. C. Trackman, "A Fluorometric Assay for Detection of Lysyl Oxidase Enzyme Activity in Biological Samples" *Anal. Biochem.* **2002**, *300*, 245–251.

[40] L. M. Poller, H. Wennemers, "Lysyl Oxidase LOXL2 Selectively Oxidizes Primary, α-Unbranched Amines and Prefers Cationic Substrates" *Biochemistry* **2026**, *65*, 779–785.

[45] J. Engel, H. Chen, D. J. Prockop, H. Klump, "The triple helix ⇌ coil conversion of collagen-like polytripeptides in aqueous and nonaqueous solvents. Comparison of the thermodynamic parameters and the binding of water to (L-Pro-L-Pro-Gly)$_n$ and (L-Pro-L-Hyp-Gly)$_n$" *Biopolymers* **1977**, *16*, 601–622.

[46] S. Frank, R. A. Kammerer, D. Mechling, T. Schulthess, R. Landwehr, J. Bann, Y. Guo, A. Lustig, H. P. Bächinger, J. Engel, "Stabilization of short collagen-like triple helices by protein engineering" *J. Mol. Biol.* **2001**, *308*, 1081–1089.
